# Reduction of organic azides by indyl-anions. Isolation and reactivity studies of indium–nitrogen multiple bonds†
†Electronic supplementary information (ESI) available: Full experimental details, copies of NMR spectra, details of X-ray experiments and additional figures, *xyz*-coordinates from DFT calculations. CCDC 1863650–1863661. For ESI and crystallographic data in CIF or other electronic format see DOI: 10.1039/c8sc04078h


**DOI:** 10.1039/c8sc04078h

**Published:** 2018-11-12

**Authors:** Mathew D. Anker, Matthias Lein, Martyn P. Coles

**Affiliations:** a School of Chemical and Physical Sciences , Victoria University of Wellington , P. O. Box 600 , Wellington , New Zealand . Email: martyn.coles@vuw.ac.nz

## Abstract

The synthesis of a new potassium–indyl complex, K[In(NON^Ar^)] (NON^Ar^ = [O(SiMe_2_NAr)_2_]^2–^, Ar = 2,6-^i^Pr_2_C_6_H_3_) and its reactivity with organic azides RN_3_ is reported. When R = 2,6-bis(diphenylmethyl)-4-^*t*^Bu-phenyl, a dianionic alkyl-amide ligand is formed *via* C–H activation across a transient In–N_imide_ bond. Reducing the size of the R-group to 2,4,6-trimethylphenyl (mesityl, Mes) enables oxidation of the indium and elimination of dinitrogen to afford the imide species, K[In(NON^Ar^)(NMes)]. The anion contains a short In–N_imide_ bond, shown computationally to contain appreciable multiple bond character. Reaction of isolated imides with an additional equivalent of azide (R = Mes, SiMe_3_) generates tetrazenido-indium compounds K[In(NON^Ar^){κ-*N*,*N*′-N_4_(Mes)(R)-1,4}], shown by X-ray crystallography to contain planar InN_4_ heterocycles in the anion.

## Abstract 2

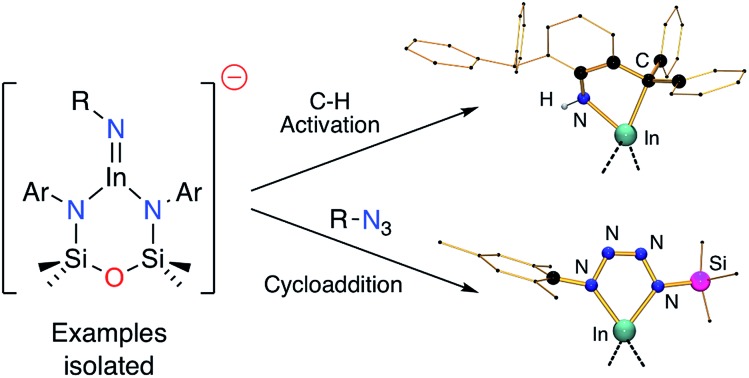
Stepwise reaction of an indyl-anion with organic azides initially forms the indium imide, which undergoes (2 + 3)-cycloaddition to generate the indium tetrazenide.

## Introduction

Low valent compounds of the group 13 elements aluminium, gallium and indium exhibit a wide range of chemical reactivity.^1^ When present in the +1 oxidation-state,^2^ the electron-configuration of the metallic element implies the presence of a lone-pair of electrons in an *n*s orbital, prompting comparisons with neutral group 14 carbenoid species.^3^ Consequently, a rich area of coordination chemistry has developed, particularly focussed on the Ga(i) compounds.^4^ In addition to the ability of these compounds to behave as ligands, the lighter homologues are potent reducing agents, readily giving up two electrons to attain a more stable +3 oxidation-state. This reactivity has been harnessed in a wide range of chemical reactions,^5^ many of which are unique to this class of compound.^6^

The most common members of this class of compound are represented by the general formula M(X), where the charge on the metal is balanced by a mono-anionic ligand, [X]^–^. These ligands are typically bulky, a requirement to limit (or prevent) aggregation and protect the metal from unwanted redox chemistry. This concept is best illustrated in the context of this work by the series M(BDI^Ar^) (BDI = β-diketiminate, [HC(CMeNAr)]^–^, Ar = 2,6-^i^Pr_2_C_6_H_3_), for which mono-metallic Al,5*f* Ga5*d* and In5*a* compounds are known.

**Fig. 1:**
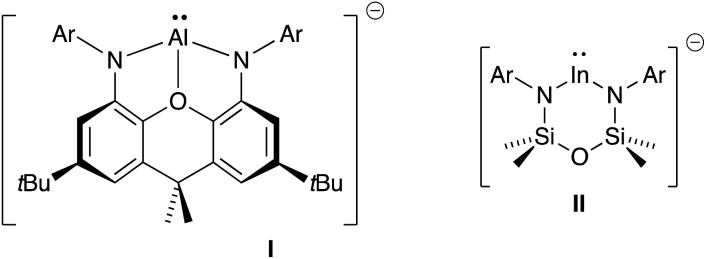
The first reported aluminyl- and indyl-anions (Ar = 2,6-^i^Pr_2_C_6_H_3_).

A recent development in the chemistry of mono-valent aluminium and indium is to employ a dianionic ligand [X_2_]^2–^ to support M(I) metal centres, generating an overall negative charge on the metal-containing species, [M(X_2_)]^–^. Whilst this class of compound has been well studied for gallium,4a,4b,^7^ the corresponding aluminyl-8 and indyl-9 anions have only been recently isolated (Fig. 1), and hence the chemistry of these compounds is in its infancy.^10^ Initial studies of [Al(X_2_)]^–^ and [In(X_2_)]^–^ systems indicate significant lone-pair character at the metal (from DFT calculations), with preliminary reactivity consistent with an Al(i) or In(i) nucleophile. We report in this contribution an investigation of the reducing potential of a new potassium indyl compound towards organic azides. This class of substrate was selected to target synthetically challenging indium imide species.

**Fig. 2:**
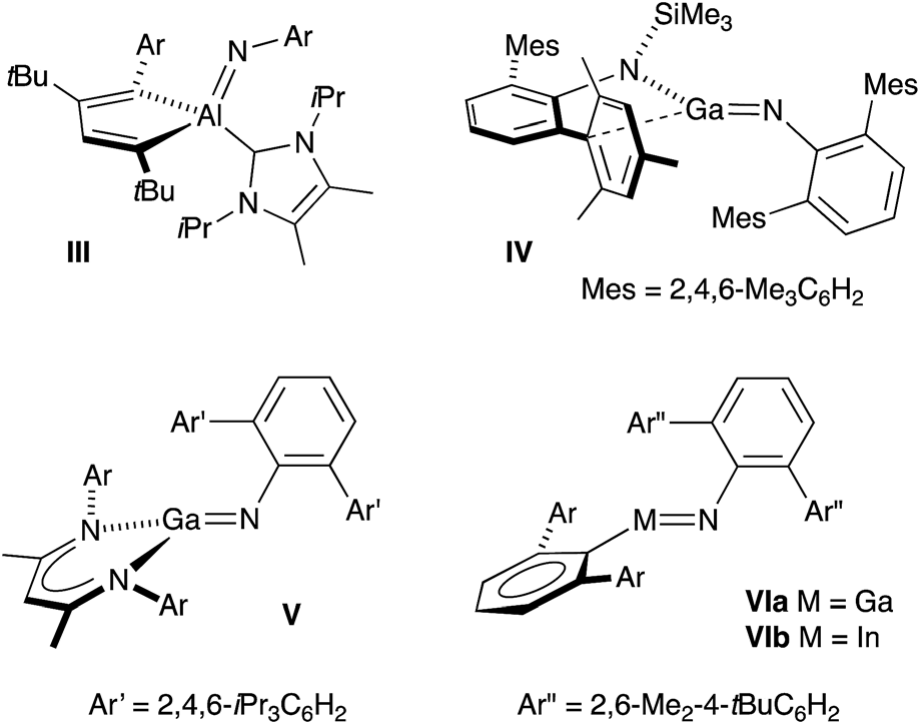
Structurally characterized monomeric compounds of Al, Ga and In containing unsupported M–N_imide_ bonds.

Monomeric group 13 metal imides [M(X)(NR)]_*n*_ (X = ancillary ligand; M = Al, Ga or In; R = organic fragment; *n* = 1) are of academic and practical interest in several research areas. They formally contain metal–nitrogen multiple-bonds,^11^ and are implicated as intermediates in the formation of electronically important AlN, GaN and InN materials.^12^ Isolation of these compounds remains, however, synthetically challenging and only five examples have been crystallographically characterized since the first structural report in 2001 (**III–VI**, Fig. 2).^13^ Furthermore, these unusual compounds are restricted to a single example of an indium imide (**VIb**).13*d* Structural characterization showed that the In–N bond distance in **VIb** (1.928(3) Å) was significantly shorter than the range observed for monomeric In amides (2.05–2.09 Å), and that the C–In–N–C core adopted a *trans*-bent geometry. These data were consistent with In–N multiple bond character.

The isolation of **III–VI** was achieved through kinetic stabilization of the M–N_imide_ bonds using sterically demanding ligands that prevent formation of ring- and cage-structures containing *μ*_2_- and *μ*_3_-NR ligands.^14^ A major limitation of this approach is that the bulk required to protect the imide bond from intermolecular aggregation renders it inaccessible to potential substrates, preventing any coherent study of its reactivity. It is encouraging to note, however, that in the absence of external substrates, intramolecular activation of ligand substituents can occur. This strongly suggests that once formed, the M–N_imide_ functional group is highly reactive.^15^

**Scheme 1:**
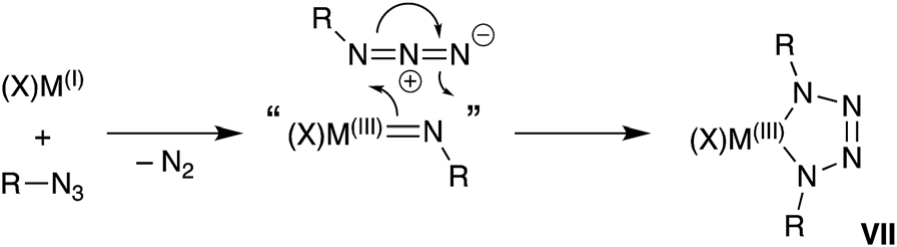
Synthesis of group 13 metal-imides from organic azides and proposed conversion to metallotetrazenes (**VII**).

A general synthetic methodology to group 13 imides is the reduction of organic azides by a monovalent M(I) metal complexes (Scheme 1).13b–d,^16^ These reactions proceed with elimination of N_2_ and oxidation of the metal M(III), which occurs with a concurrent increase in the coordination number of the metal. It is of note that, if insufficient steric protection is provided during synthesis, *in situ* addition of unreacted azide in the reaction mixture to the transient ‘M=NR’ bonds can occur (Scheme 1).^17^ For Al and Ga, this has enabled the isolation of metallotetrazenes **VII** (also referred to as metal-containing tetrazoles),^17^,^18^ which are rationalized as the product of a (2 + 3)-cycloaddition. This chemistry has not been extended to indium.

In this contribution we report a new potassium indyl salt and its controlled (stepwise) reactivity with organic azides. The initial products are characterised as a new class of anionic indium(iii) imide, shown crystallographically and computationally to contain In–N_imide_ multiple bonds. Reaction of isolated examples with additional azide proceeds *via* a (2 + 3)-cycloaddition pathway to generate tetrazenido-indium salts, containing the first structurally characterized examples of the InN_4_-heterocycle.

## Results and discussion

### Synthesis of a new potassium indyl salt

The NON^Ar^-ligand (NON^Ar^ = [O(SiMe_2_NAr)_2_]^2–^; Ar = 2,6-^i^Pr_2_C_6_H_3_) stabilizes anionic indyl species as the indyllithium complex In(NON^Ar^)(Li{THF}_3_), or in the ion-separated salt [K(crypt-222)][In(NON^Ar^)] (crypt-222 = [2.2.2]-cryptand).^9^ Reactivity studies of these species have been hampered by their inherent instability, prompting us to examine an alternative source of the indyl anion. A modified procedure was therefore developed that avoids lithium reagents, and does not require the use of expensive crypt-222 reagents to stabilize the salt.

The dipotassium salt, K_2_[(NON^Ar^)(THF)_*n*_] (**1**), is readily obtained from the reaction of KH with the ligand pre-cursor (NON^Ar^)H_2_.^19^ NMR analysis of the freshly prepared salt is consistent with incorporation of a single molecule of THF (*n* = 1), and the reagent can be used without further purification. However, crystallization from THF affords the Tris–THF adduct (*n* = 3) which forms a dimer [**1**_{THF}_3_]_2_ in the solid-state (Fig. S3†). The potassium salt can also be isolated as the bis-diethylether adduct K_2_[(NON^Ar^)(Et_2_O)_2_] which forms a polymer in the solid-state (Fig. S4†), or as the 18-crown-6 (18-*c*-6) adduct that crystallizes as the dimer [K_2_{(NON^Ar^)(18-*c*-6)}]_2_ (Fig. S5†).

**Fig. 3:**
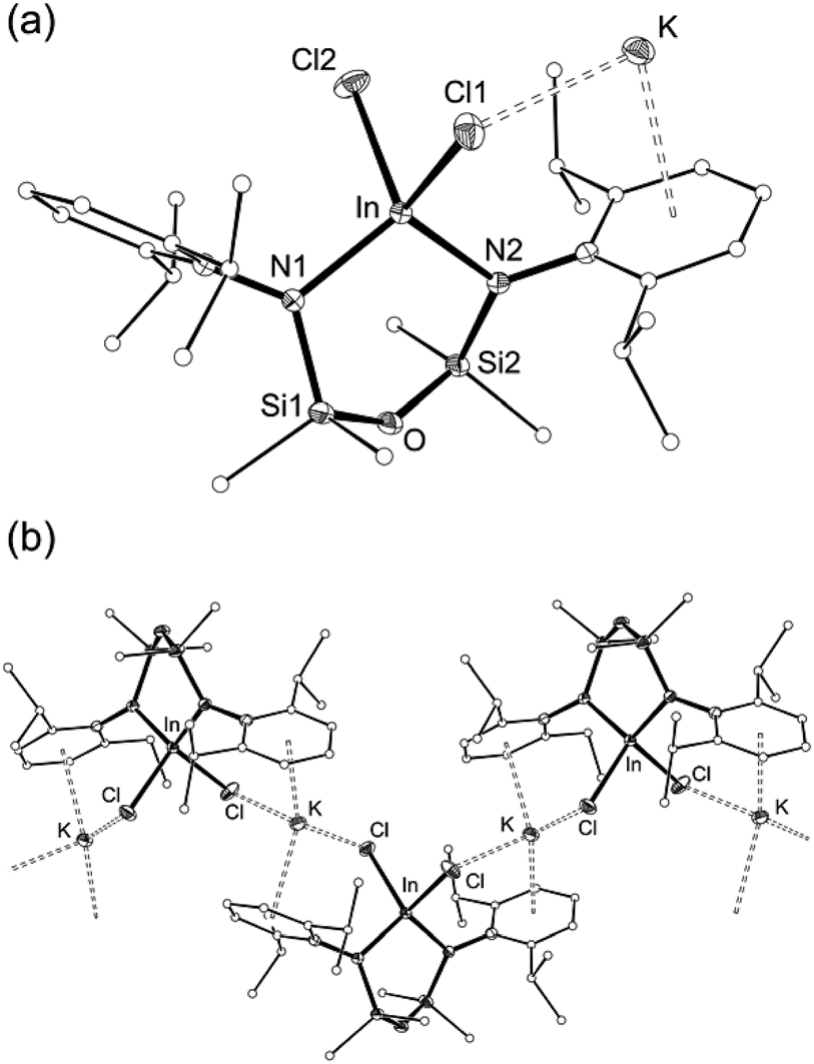
(a) Displacement ellipsoid plot (30% probability, carbon atoms reduced, hydrogen atoms omitted) of K[In(NON^Ar^)Cl_2_] (**2**). (b) Segment of polymeric chain (···[**2**]_3_···), showing intermolecular K···Cl and K···C interactions.

The reaction of **1** with InCl_3_ affords a new indium-containing complex, **2**. The NMR spectra show a symmetrical environment for the ligand backbone with a single peak for the SiMe_2_ groups. Although this is consistent with the three-coordinate species ‘In(NON^Ar^)Cl’, elemental analysis was inconsistent with this formula and the compounds was therefore analysed by single-crystal X-ray diffraction (Fig. 3 and Table 1).

**Table 1:** Selected bond length (Å) and angles (°) for K[In(NON^Ar^)Cl_2_] (**2**) and [K{In(NON^Ar^)}]_2_ ([**3**]_2_)

Compound	**2**	[**3**]_2_
In^*a*^–N1	2.0820(17)	2.222(3)
In^*a*^–N2	2.0826(17)	2.193(2)
In2–N3	—	2.240(3)
In2–N4	—	2.182(3)
In–Cl1	2.3977(5)	—
In–Cl2	2.4010(6)	—
N1–In^*a*^–N2	107.57(7)	98.43(9)
N3–In2–N4	—	98.24(9)

^*a*^ **2** = In, [**3**]_2_ = In1.

The asymmetric unit of **2** contains the four-coordinate indium anion [In(NON^Ar^)Cl_2_]^–^ (Fig. 3a). The charge is balanced by a potassium atom that forms π–aryl interactions with an Ar-groups of the diamide ligand, and has a close-contact with a chloride ligand. The crystal structure shows a 1-D polymer [**2**]_*n*_ with additional interactions between the K-atom and aryl-/chloride groups from neighbouring molecules (Fig. 3b).

Reduction of **2** with two equivalents of potassium yields the new indyl compound K[In(NON^Ar^)] (**3**) as a hexane soluble, yellow solid. The ^1^H NMR spectrum of **3** displays a single resonance for the SiMe_2_ substituents, indicative of a symmetrical environment for the NON^Ar^-ligand. There are no resonances attributable to In–H hydride ligands (Fig. S10†).^20^

**Fig. 4:**
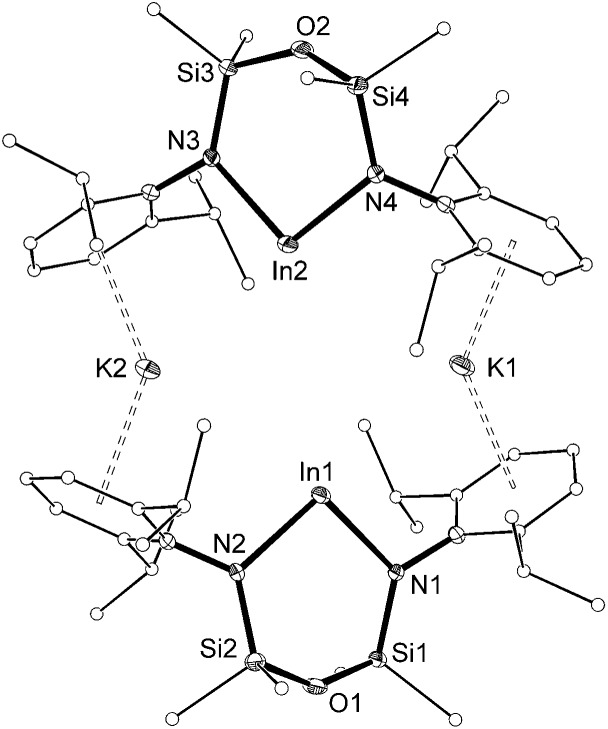
Displacement ellipsoid plot (30% probability, carbon atoms reduced, hydrogen atoms omitted) of [K{In(NON^Ar^)}]_2_ ([**3**]_2_).

The molecular structure of **3** (Fig. 4) is reminiscent of the recently reported aluminyl anion.^8^ The asymmetric unit consists of two [In(NON^Ar^)]^–^ anions linked by potassium cations that are involved in π–aryl interactions to flanking Ar groups (C···K distances 3.109(4)–3.346(3) Å). The In···In separation is 4.710(3) Å, with In–N bond lengths (2.182(3)–2.240(3) Å) consistent with anionic In(i) metal centres.^9^ There are no bonding interactions between the indium and the oxygen-atom of the backbone (In···O = 3.557(2) and 3.577(2) Å), consistent with a strictly two-coordinate indyl anion.

### Reactivity of the indyl anion with organic azides

**Scheme 2:**
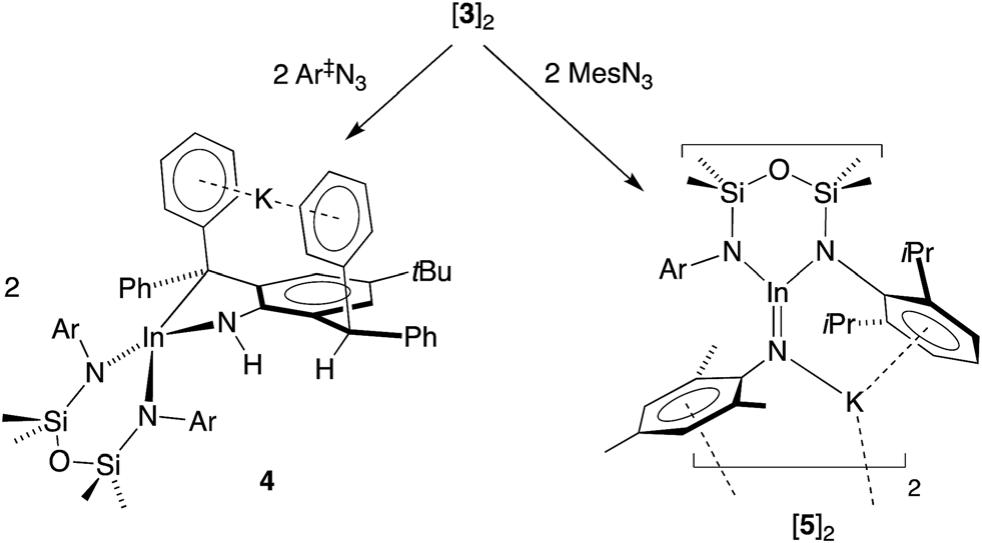
Structurally characterized monomeric complexes of Al, Ga and In containing unsupported M–N_imide_ bonds.

Our initial attempt to isolate an imide from **3** was made using an equimolar amount of the sterically demanding 2,6-bis(diphenylmethyl)-4-^*t*^Bu-phenyl azide (Ar^‡^N_3_, Scheme 2). The reagents were combined at –78 °C, allowed to warm to room temperature and stir for 1 h. The ^1^H NMR spectrum of colourless crystals **4** obtained on workup showed a loss symmetry for the NON-backbone (*δ*_H_ 0.58 and 0.45, 6H, SiMe_2_) and a reduction in the intensity of the CHPh_2_ resonance (*δ*_H_ 5.56, 1H). A new peak at 3.24 ppm (with no corresponding carbon resonance in HSQC experiments, Fig. S15†) is assigned to an NH functionality.

**Fig. 5:**
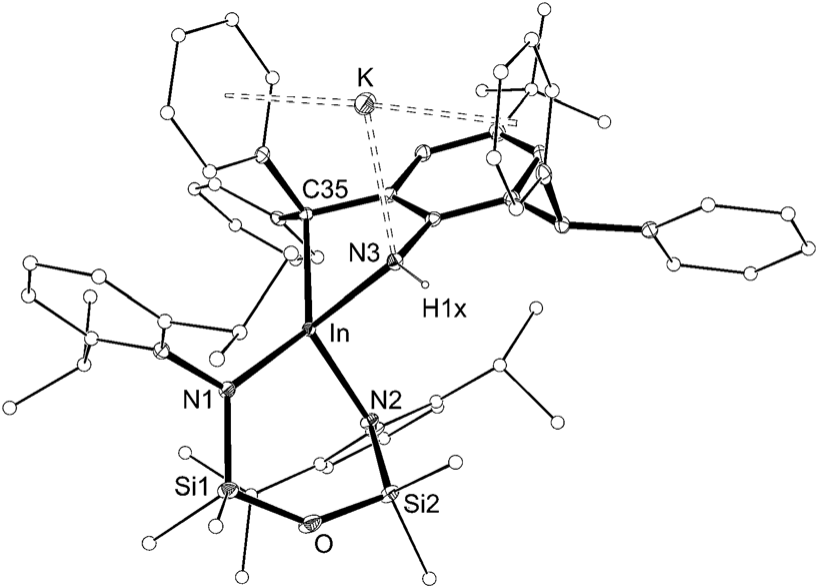
Displacement ellipsoid plot (30% probability, carbon atoms reduced, hydrogen atoms omitted) of K[In(NON^Ar^)(κ^2^-C,N–N(H){C_6_H_2_(CPh_2_)(CHPh_2_)-^*t*^Bu-2,6,4})] (**4**).

The structure of **4** was determined by X-ray diffraction and shows a κ^2^-C,N–N(H){C_6_H_2_(CPh_2_)(CHPh_2_)-^*t*^Bu-2,6,4} ligand chelating to a four-coordinate, anionic indium(iii) centre (Fig. 5 and Table 2). The potassium counterion is located between two aryl-substituents of the alkyl-amido ligand (C···K distances 3.148(2)–3.581(2) Å). The In–N3 distance (2.1855(14) Å) is consistent with a single bond to an amide nitrogen, and the location of electron density assigned to H1*x* in the difference map further supports this conclusion. Similar intramolecular activation has been observed at Al^21^ and Sn^22^ amido derivatives of the Ar^‡^ group, although in these instances the mechanism leading to the products are not known. We propose that formation of **4** occurs *via* intramolecular addition of a methine CHPh_2_ across the reactive ‘In=NAr^‡^’ bond of a transient indium imide.15*b*

**Table 2:** Selected bond length (Å) and angles (°) for K[In(NON^Ar^)(κ^2^-C,N–N(H){C_6_H_2_(CPh_2_)(CHPh_2_)-^*t*^Bu-2,6,4})] (**4**)

In–N1	2.1674(14)	In–N2	2.1676(14)
In–N3	2.1855(14)	In–C35	2.2875(16)
N1–In–N2	102.24(5)	N1–In–N3	112.64(5)
N2–In–N3	106.25(5)	N1–In–C35	132.97(6)
N2–In–C35	119.53(6)	N3–In–C35	77.12(6)

To mitigate complications from ligand activation, the reaction was repeated with the sterically less intrusive 2,4,6-trimethylphenyl azide (mesityl azide, MesN_3_) under the conditions described above (Scheme 2). Concentration of the resulting solution and storage at –30 °C gave deep orange crystals (**5**). The ^1^H NMR spectrum is consistent with a symmetrical NON-backbone (*δ*_H_ 0.33, 12H, SiMe_2_) and a freely rotating Mes group (*δ*_H_ 1.57, 6H, 2,6-C_6_H_2_Me_2_).

**Fig. 6:**
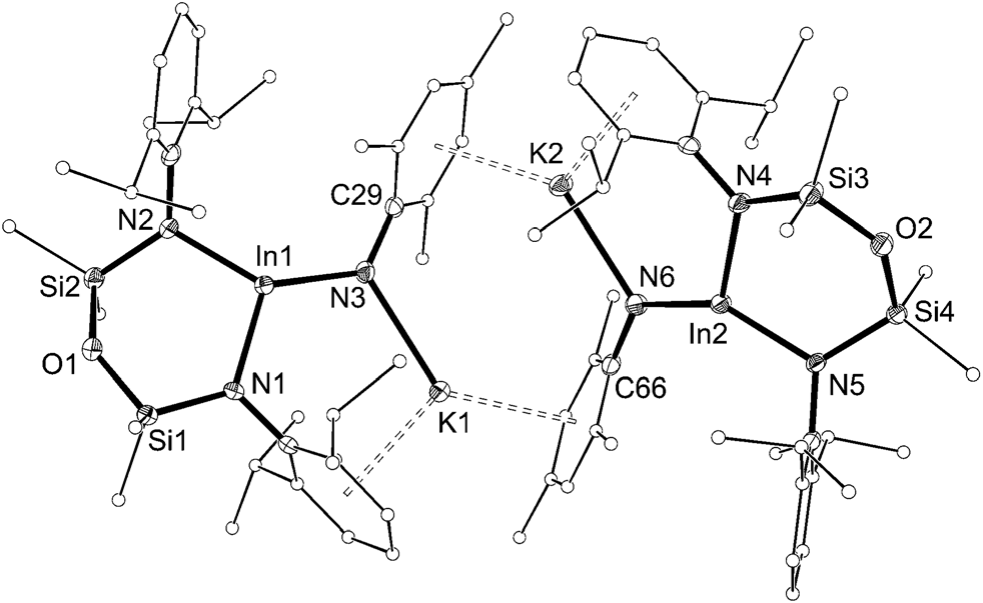
Displacement ellipsoid plot (30% probability, carbon atoms reduced, hydrogen atoms omitted) of [K{In(NON^Ar^)(NMes)}]_2_ ([**5**]_2_).

The solid-state structure of **5** confirmed the formation of an imido–indium complex (Fig. 6 and Table 3). The compound crystallizes as the non-symmetry related dimer [**5**]_2_, with the potassium counter-ions involved in π–aryl interactions with Ar and Mes substituents (C···K distances 3.102(3)–3.296(3) Å). The indium centres are distorted trigonal planar (Σ_angles_ 358.7°), with In–N_imide_ bond lengths of 1.986(2) and 1.999(2) Å to N3 and N6, respectively. These represent an average shortening of 3.6% compared with the In–N bonds in the three coordinate amide In(NHMes*)_3_ (Mes* = 2,4,6-^*t*^Bu_3_C_6_H_2_),^23^ although are longer than the neutral imide complex **VIb** (1.928(3) Å)13*d* in which the metal is two-coordinate. The imido-nitrogen atoms are bent, with In–N–C angles of 123.9(2)° and 123.8(2)° at N3 and N6, respectively.

**Table 3:** Selected bond length (Å) and angles (°) for [K{In(NON^Ar^)(NMes)}]_2_ ([**5**]_2_) and [K(crypt-222)][In(NON^Ar^)(NMes)] (**6**)

Compound	[**5**]_2_	**6**
In^*a*^–N1	2.090(2)	2.1026(16)
In^*a*^–N2	2.077(2)	2.1152(16)
In^*a*^–N3	1.986(2)	1.9907(18)
In2–N4	2.097(2)	—
In2–N5	2.082(2)	—
In2–N6	1.999(2)	—
N1–In^*a*^–N2	104.85(10)	100.36(6)
N1–In^*a*^–N3	115.80(10)	125.54(7)
N2–In^*a*^–N3	138.01(10)	130.25(7)
N4–In2–N5	102.70(9)	—
N4–In2–N6	115.29(10)	—
N5–In2–N6	140.66(10)	—

^*a*^[**5**]_2_ = In1, **6** = In.

**Fig. 7:**
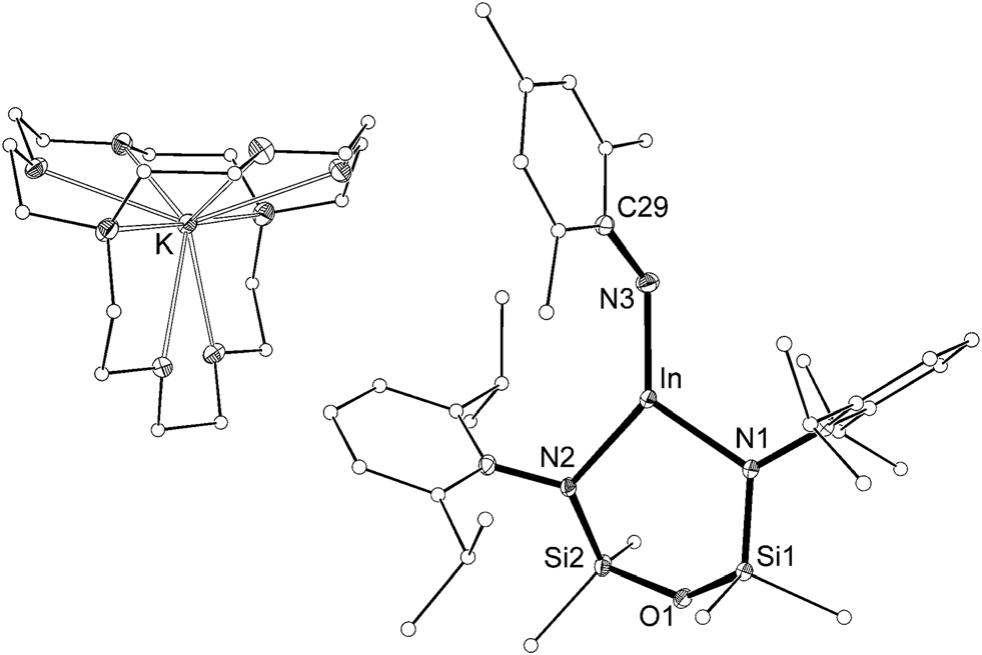
Displacement ellipsoid plot (30% probability, carbon atoms reduced, hydrogen atoms omitted) of [K(crypt-222)][In(NON^Ar^)(NMes)] (**6**).

The length of the In–N_imide_ bonds in **5** may be influenced by interactions with the potassium cations (N3–K1 2.661(3) Å, N6–K2 2.646(3) Å), which are located closer to the nitrogen atom than in other potassiated imides (range 2.732(3)–3.069(11) Å).^24^ To isolate the imido-bond from N···K interactions, **5** was crystallized in the presence of [2.2.2]-cryptand. The crystal structure of the product confirmed formation of the separated ion-pair [K(crypt-222)][In(NON^Ar^)(NMes)] (**6**, Fig. 7). Interestingly the In–N_imide_ bond length (1.9907(18) Å) remains unchanged (within 3*σ*) and the In–N–C angle is still bent (In–N3–C29 127.43(14)°), suggesting that the N···K interactions in **5** have little effect on the structural component of the indium–nitrogen bond.

### Computational analysis of In–N_imide_ bond

Optimisation and subsequent analysis using density functional theory confirmed the multiple-bond character of the In–N_imide_ unit in **5** and the isolated anion [In(NON^Ar^)(NMes)]^–^ from compound **6** ([**6**]^–^) (see ESI†). This is clearly demonstrated from the increased Wiberg bond orders for this group (**5**, 0.59; [**6**]^–^ 0.71) that are substantially higher than the In–N bonds to the NON^Ar^-ligand (**5**, 0.22/0.29; [**6**]^–^, 0.25).

**Scheme 3:**
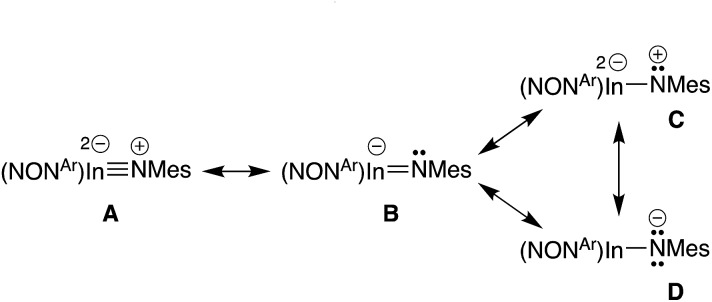
Possible resonance structures for indium imide anions.

To explore the nature of this bond in more detail, plausible resonance structures analogous to those examined for **VIb**,13*d* were submitted for NBO calculations (**A–D**, Scheme 3). The quality criterion used to compare results calculated for the different resonance structures is the percentage of non-Lewis (n-L) components, where a lower non-Lewis percentage indicates a better representation. Resonance form **C** did not yield a viable solution by this method. However, structures **A** (triple bond), **B** (double bond) and **D** (single bond) all have a low n-L contribution to their overall NBO solution (Table S2†). The best localisations were achieved for multiple-bonded **A** and **B** (n-L = 1.965% and 1.990% respectively), while **D** was only slightly less well localised (n-L = 2.094%). Although it is difficult to extract a precise numerical value for the multiplicity of the In–N_imide_ bond from these computational data, the results confirm a strong multiple-bond component in accordance with crystallographic results, and observed reactivity (*vide infra*).

**Fig. 8:**
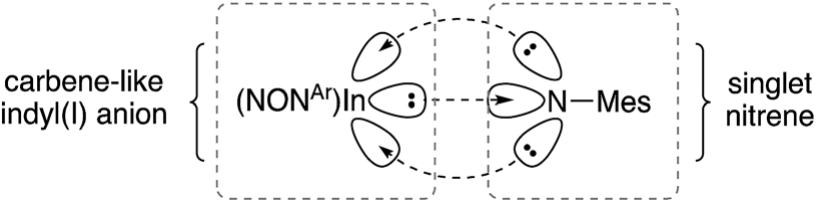
Proposed bonding model for In–N_imide_ interaction in [**6**]^–^, based on a model proposed Power and by co-workers (see ref. 13b).

Quantum Theory of Atoms In Molecules (QTAIM) analysis of **5** and [**6**]^–^ has been performed. The bond critical point between the In and N_imide_ bonds have a low ellipiticity (*ε*) of 0.079 and 0.072 for **5** and [**6**]^–^, respectively, inconsistent with a conventional In=N double where a larger value (>0.25) is predicted. These data suggest a non-elliptical cross-section of electron density in the In–N bond vector. This is consistent with a model proposed by Power and co-workers in related gallium imides related to **VIa**,13*b* in which an organogallium(i) species interacts with a singlet nitrene, with incomplete donation of electron pairs (represented by dashed lines in Fig. 8).

### Reactivity of indium imides with organic azides

As the imido-mesityl substituents are considerably less bulky than the terphenyl groups in **IV–VI**, we wished to determine whether the In–N_imide_ bond in **5** was available for controlled reactivity studies. Inspired by the proposed formation of metallotetrazenes from group 13 metal imides (Scheme 1), we investigated the reactivity of isolated samples of **5** with organic azides RN_3_ (R = Mes, SiMe_3_).

**Scheme 4:**
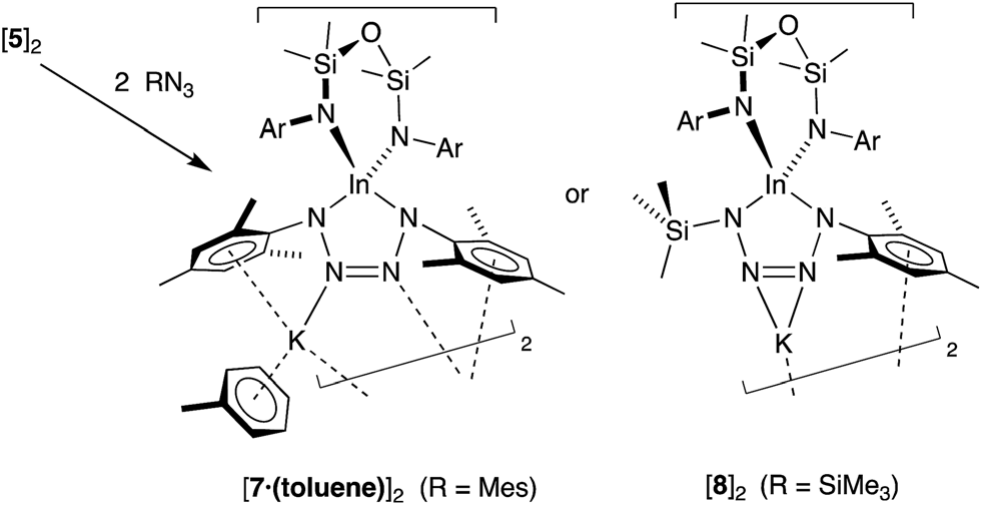
Reaction of K[In(NON^Ar^)(NMes)] (**5**) with RN_3_ (R = Mes, SiMe_3_).

**Fig. 9:**
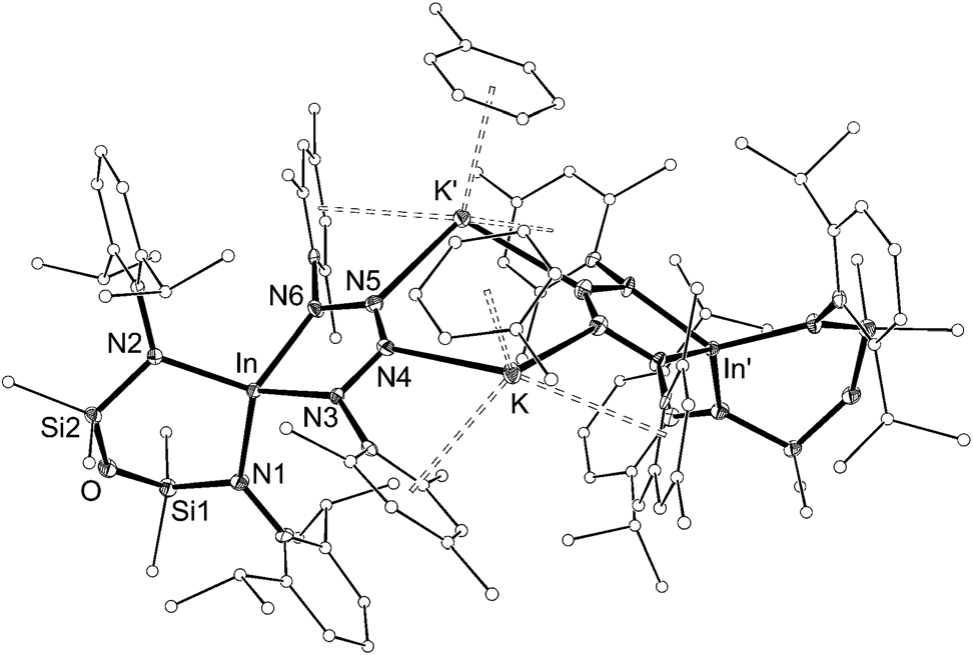
Displacement ellipsoid plot (30% probability, carbon atoms reduced, hydrogen atoms omitted) of [K{In(NON^Ar^)(κ-*N*,*N*′-N_4_{Mes}_2_–1,4)(C_7_H_8_)}]_2_ ([**7·(toluene)**]_2_).

**Fig. 10:**
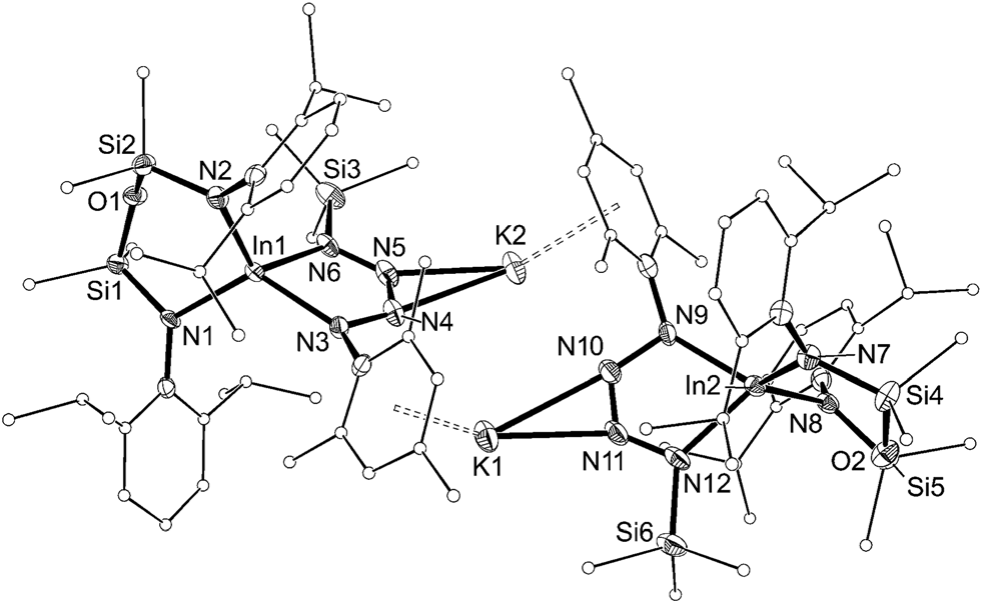
Displacement ellipsoid plot (30% probability, carbon atoms reduced, hydrogen atoms omitted) of [K{In(NON^Ar^)(κ-*N*,*N*′-N_4_{Mes}{SiMe_3_}-1,4)}]_2_ ([**8**]_2_).

Addition of a solution of RN_3_ to an orange solution of **5** at room temperature resulted in decolorization over an approximate 5 minute period (Scheme 4). NMR spectra show changes corresponding to the addition of mesityl (**7**) or SiMe_3_ (**8**) groups, consistent with their incorporation in the product. In agreement with these data, the composition of the products as the first examples of indium tetrazenido compounds was confirmed by X-ray crystallography (Fig. 9 and 10, Table 4). These results demonstrate that in this instance, the potassium atoms in **5** do not adversely affect the reactivity of the In–N_imide_ bond. We therefore propose that **5** behaves chemically as ‘In=NMes’, and that the formation of the unsymmetrical tetrazene **8** strongly endorses the previously assumed (2 + 3)-cycloaddition pathway of transient imides.

**Table 4:** Selected bond length (Å) and angles (°) for [K{In(NON^Ar^)(κ-*N*,*N*′-N_4_{Mes}_2_–1,4)(C_7_H_8_)}]_2_ ([**7·(toluene)**]_2_) and [K{In(NON^Ar^)(κ-*N*,*N*′-N_4_{Mes}{SiMe_3_}-1,4)}]_2_ ([**8**]_2_)

Compound	[**7·(toluene)**]_2_	[**8**]_2_
In^*a*^–N1	2.143(4)	2.137(5)
In^*a*^–N2	2.134(4)	2.117(5)
In^*a*^–N3	2.127(4)	2.116(5)
In^*a*^–N6	2.140(4)	2.147(5)
In2–N7	—	2.091(6)
In2–N8	—	2.132(5)
In2–N9	—	2.117(6)
In2–N12	—	2.146(6)
N3–N4	1.391(6)	1.398(8)
N4–N5	1.273(6)	1.277(9)
N5–N6	1.376(6)	1.373(9)
N9–N10	—	1.402(8)
N10–N11	—	1.272(9)
N11–N12	—	1.347(10)
N1–In^*a*^–N2	103.12(16)	106.1(2)
N3–In^*a*^–N6	74.67(16)	76.0(2)
N7–In2–N8		105.8(2)
N9–In2–N12		74.9(2)

^*a*^[**7·(toluene)**]_2_ = In1, **6** = In.

The dimesityl derivative [**7·(toluene)**]_2_ lies on a two-fold rotation axis that forms a dimeric unit, with K···π–aryl interactions between Mes-groups and an incorporated toluene molecule that encapsulates the cation (C···K distances 3.006(5)–3.993(5) Å). Crystallization of [**7·(toluene)**]_2_ in the presence of 18-crown-6 (18-*c*-6) disrupts dimer formation to afford [K(18-*c*-6)][In(NON^Ar^)(N_4_{Mes}_2_–1,4)] (**7** (18-*c*-6)). This salt forms a contact ion-pair linked by K···N interactions to the two central nitrogen atoms of the InN_4_-tetrazene ring (2.820(2) Å and 2.939(2) Å, Fig. S27†). The trimethylsilyl derivative K[In(NON^Ar^)(N_4_{Mes}{SiMe_3_}-1,4)] also crystallizes as the dimer [**8**]_2_, with the potassium cations linking non-symmetry related units through a combination of K···N (2.641(6)–2.913(6) Å) and K···π–aryl (3.024(9)–3.264(8) Å) interactions.

In all cases the anion comprises two approximately orthogonal rings fused at a four-coordinate indium centre. The metallotetrazene rings are essentially planar, with nitrogen–nitrogen bond lengths indicating double-bond character between atoms in the 3- and 4-positions of the heterocycle (see **VII**, Scheme 1). These parameters are consistent with neutral aluminum-17,18 and gallium-18*b* derivatives, although we note that compounds **7** and **8** represent the first structurally characterized indium compounds containing the tetrazenide ligand, and are unique examples where the MN_4_-heterocycle is a component of an anionic species.

## Conclusions

This work describes the first detailed reactivity study of an indyl-anion. We confirm that the negative charge associated with the indium centre does not adversely affect their ability to act as a reducing agent towards organic azides. The reactions proceed cleanly with elimination of dinitrogen and oxidation of the indium(i) to In(iii). The isolated compounds have been structurally verified as a new class of anionic indium imide, shown computationally to contain In–N_imide_ multiple bonds. Furthermore, we demonstrate that the reduced size of the imide-substituent in this work compared with previous examples allows access to the In–N_imide_ bond, demonstrated by the reaction with additional equivalents of azide. The products from this (2 + 3)-cycloaddition are the first time that this reaction has been extended to indium, and crystallographic analysis confirms a planar InN_4_-heterocycle as a component of the anion.

## Conflicts of interest

There are no conflicts to declare.

## Supplementary Material

Supplementary information

Crystal structure data
