# Supplementary material for: Reduction of organic azides by indyl-anions. Isolation and reactivity studies of indium–nitrogen multiple bonds
Source: Chem Sci. 2018 Nov 12;10(4):1212–8. doi: 10.1039/c8sc04078h (PMC6349055; doi:10.1039/c8sc04078h)
Supplement: Supplementary file 1 [file SC-010-C8SC04078H-s001.pdf]

Electronic Supplementary Material (ESI) for Chemical Science.
This journal is © The Royal Society of Chemistry 2018

**Supporting Information for:**

# Reduction of Organic Azides by Indyl-Anions. Isolation and Reactivity Studies of Indium-Nitrogen Multiple Bonds

Mathew D. Anker, Matthias Lein and Martyn P. Coles*

*School of Chemical and Physical Sciences, Victoria University of Wellington, P.O. Box 600, Wellington, New Zealand*

**Table of Contents:**

S3 General Experimental Details
S4 Synthesis of $K_2(NON^{Ar})$ (**1**)
S5 **Figure S1** $^1H$ NMR spectrum of $K_2[(NON^{Ar})(THF)]$ (**1_{THF}**)
S6 **Figure S2** $^{13}C\{^1H\}$ NMR spectrum of $K_2[(NON^{Ar})(THF)]$ (**1_{THF}**)
S7 **Figure S3a** ORTEP of the asymmetric unit of $[K_2(\{NON^{Ar}\}\{THF\}_3)]_2$ ([**1_{THF}₃**]$_2$)
S8 **Figure S3b** ORTEP of polymeric $[K_2(\{NON^{Ar}\}\{THF\}_3)]_2$
S9 **Figure S4a** ORTEP of the asymmetric unit of $K_2[(NON^{Ar})(Et_2O)_2]$ (**1_{Et₂O}₂**)
S10 **Figure S4b** ORTEP of polymeric $K_2[(NON^{Ar})(Et_2O)_2]$ (**1_{Et₂O}₂**)
S11 **Figure S5a** ORTEP of the asymmetric unit of $K_2[(NON^{Ar})$(18-crown-6)] (**1_{18-c-6}**)
S12 **Figure S5b** ORTEP of dimeric $K_2[(NON^{Ar})$(18-crown-6)] (**1_{18-c-6}**)
S13 Synthesis of $K[In(NON^{Ar})Cl_2]$ (**2**)
S14 **Figure S6** $^1H$ NMR spectrum of $K[In(NON^{Ar})Cl_2]$ (**2**)
S15 **Figure S7** $^{13}C\{^1H\}$ NMR spectrum of $K[In(NON^{Ar})Cl_2]$ (**2**)
S16 **Figure S8a** ORTEP of the asymmetric unit of $K[In(NON^{Ar})Cl_2]$ (**2**)
S17 **Figure S8b** ORTEP of polymeric $K[In(NON^{Ar})Cl_2]$ (**2**)
S18 **Figure S9** ORTEP of $K[In(NON^{Ar})Cl_2]$·benzene (**2_benzene**)
S19 Synthesis of $K[In(NON^{Ar})]$ (**3**)
S20 **Figure S10** $^1H$ NMR spectrum of $K[In(NON^{Ar})]$ (**3**)
S21 **Figure S11** $^{13}C\{^1H\}$ NMR spectrum of $K[In(NON^{Ar})]$ (**3**)
S22 **Figure S12** ORTEP of $[K(In\{NON^{Ar}\})]_2$ ([**3**]$_2$)
S23 Synthesis of $K[In(NON^{Ar})(N(H)\{2\text{-}(CPh_2)\text{-}6\text{-}(CHPh_2)\text{-}4\text{-}t\text{Bu}C_6H_2\}]$ (**4**)
S24 **Figure S13** $^1H$ NMR spectrum of $K[In(NON^{Ar})(N(H)\{2\text{-}(CPh_2)\text{-}6\text{-}(CHPh_2)\text{-}4\text{-}t\text{Bu}C_6H_2\}]$ (**4**)
S25 **Figure S14** $^{13}C\{^1H\}$ NMR spectrum of $K[In(NON^{Ar})(N(H)\{2\text{-}(CPh_2)\text{-}6\text{-}(CHPh_2)\text{-}4\text{-}t\text{Bu}C_6H_2\}]$ (**4**)
S26 **Figure S15** HSQC spectrum of $K[In(NON^{Ar})(N(H)\{2\text{-}(CPh_2)\text{-}6\text{-}(CHPh_2)\text{-}4\text{-}t\text{Bu}C_6H_2\}]$ (**4**)
S27 **Figure S16** ORTEP of $K[In(NON^{Ar})(N(H)\{2\text{-}(CPh_2)\text{-}6\text{-}(CHPh_2)\text{-}4\text{-}t\text{Bu}C_6H_2\}]$ (**4**)
S28 a) Synthesis of $K[In(NON^{Ar})(NMes))]$ (**5**)
b) Synthesis of [K(222-crypt)]$[In(NON^{Ar})(NMes))]$ (**6**)
S29 **Figure S17** $^1H$ NMR spectrum of $K[In(NON^{Ar})(NMes))]$ (**5**)
S30 **Figure S18** $^{13}C\{^1H\}$ NMR spectrum of $K[In(NON^{Ar})(NMes))]$ (**5**)
S31 **Figure S19** ORTEP of $[K(In\{NON^{Ar}\}\{NMes\})]_2$ ([**5**]$_2$)
S32 **Figure S20** ORTEP of [K(crypt-222)]$[In\{NON^{Ar}\}\{NMes\})]$ (**6**)

S33 **Figure S21** View of In(NMes) components of [**5**]$_2$ and **6**
S34 Representative procedure for the preparation of K[In(NON$^{Ar}$)(N$_4${Mes}$_2$-1,4] (**7**) and K[In(NON$^{Ar}$)(N$_4${Mes}{SiMe$_3$}-1,4] (**8**)
S35 **Figure S22** $^1$H NMR spectrum of K[In(NON$^{Ar}$)(N$_4${Mes}$_2$-1,4] (**7**)
S36 **Figure S23** $^{13}$C{$^1$H} NMR spectrum of K[In(NON$^{Ar}$)(N$_4${Mes}$_2$-1,4] (**7**)
S37 **Figure S24a** ORTEP of the asymmetric unit of [K(In{NON$^{Ar}$}{N$_4$(Mes)$_2$-1,4})]$_2$ ([**7·(toluene)**]$_2$)
S38 **Figure S24b** ORTEP of the core of [K(In{NON$^{Ar}$}{N$_4$(Mes)$_2$-1,4})]$_2$ ([**7·(toluene)**]$_2$)
S39 **Figure S25** $^1$H NMR spectrum of K[In(NON$^{Ar}$)(N$_4${Mes}{SiMe$_3$}-1,4] (**8**)
S40 **Figure S26** $^{13}$C{$^1$H} NMR spectrum of K[In(NON$^{Ar}$)(N$_4${Mes}{SiMe$_3$}-1,4] (**8**)
S41 **Figure S27a** ORTEP of the asymmetric unit of [K(In{NON$^{Ar}$}{N$_4$(Mes)(SiMe$_3$)-1,4})]$_2$ ([**8**]$_2$)
S42 **Figure S27b** ORTEP of the core of [K(In{NON$^{Ar}$}{N$_4$(Mes)(SiMe$_3$)-1,4})]$_2$ ([**8**]$_2$)
S43 **Figure S28** ORTEP of [K(18-crown-6)][In(NON$^{Ar}$)(N$_4${Mes}$_2$-1,4)] (**7·(18-c-6)**)
S44 Crystallography
S46 Computational Methods
S47 **Table S1** Calculated structural parameters and charges for **5** and [**6**]$^-$
S48 **Figure S29** QTAIM derived molecular graphs of K[In(NON$^{Ar}$)(NMes)] (**5**).
S49 **Figure S30** QTAIM derived molecular graphs of [In(NON$^{Ar}$)(NMes)]$^-$ ([**6**]$^-$).
S50 **Figure S31** Contour plot of the Laplacian of electron density of [In(NON$^{Ar}$)(NMes)]$^-$ ([**6**]$^-$)
S51 **Table S2** NBO Plots for [In(NON$^{Ar}$)(NMes)]$^-$ ([**6**]$^-$)
S52 References
S53 *x*, *y*, *z* coordinates from DFT calculations

**General Experimental Details**

All manipulations were carried out under dry nitrogen using standard Schlenk-line and cannula techniques, or in a conventional nitrogen-filled glovebox. Solvents were dried over appropriate drying agents and degassed prior to use. NMR spectra were recorded using a Bruker Avance DPX 300 MHz spectrometer at 300.1 ($^{1}H$) and 75.4 ($^{13}C$) MHz or a Varian VNMRS 500 MHz spectrometer at 500.1 ($^{1}H$) and 75.4 ($^{13}C$) MHz. Proton and carbon chemical shifts were referenced internally to residual solvent resonances and all coupling are reported in Hz. Elemental analyses were performed by S. Boyer at London Metropolitan University. $(NON^{Ar})H_2$[S1] was prepared according to literature procedures.

*Synthesis of $K_2$(NON$^{Ar}$)(THF) (**1**)*

A solution of (NON$^{Ar}$)$H_2$ (1.00 g, 2.06 mmol) in THF was added to 2.1 equivalents of solid KH (0.173 g, 4.33 mmol). The solution was stirred for 2 days. The solution was filtered, and the solvent was removed *in vacuo*. The resultant solid **1** was washed with cold hexane to give $K_2$(NON$^{Ar}$)(THF) as a colourless solid. Yield 1.12 g, 86 %.

Single crystals suitable for X–ray diffraction were grown from a saturated THF solution stored at –30 °C overnight, and shown to correspond to the formula $K_2$[(NON$^{Ar}$)(THF)$_3$] (**1_{THF}$_3$**).

$^1$H NMR ($CD_3CN$, 600 MHz, 333 K): $\delta$ 7.06 (d, *J* = 7.3, 2H, $C_6H_3$), 7.00 (dd, *J* = 8.3, 6.9, 1H, $C_6H_3$), 6.97 (d, *J* = 7.7, 2H, $C_6H_3$), 6.67 (t, *J* = 7.7, 1H, $C_6H_3$), 3.64 (m, 4H, THF-C*H$_2$*), 3.56 (sept, *J* = 6.8, 2H, C*H*Me$_2$), 2.95 (sept, *J* = 6.8, 2H, C*H*Me$_2$), 1.80 (m, 4H, THF-C*H$_2$*), 1.20 (d, *J* = 6.8, 12H, CH*Me$_2$*), 1.15 (d, *J* = 6.9, 12H, CH*Me$_2$*), 0.10 (s, 1H, Si*Me$_2$*), 0.09 (s, 2H, Si*Me$_2$*), 0.08 (s, 6H, Si*Me$_2$*), 0.03 (s, 3H, Si*Me$_2$*).

$^{13}$C{$^1$H} NMR ($CD_3CN$, 151 MHz, 333 K): $\delta$ 145.7, 142.0, 140.3, 133.2, 133.1, 124.7, 123.8, 123.5, 118.7 (*C$_6$*H$_3$), 68.3 (THF-*C*H$_2$), 28.8, 28.3(*C*HMe$_2$), 26.3 (THF-*C*H$_2$), 24.0, 22.8 (CH*Me$_2$*), 3.6, 0.20 (Si*Me$_2$*).

Extreme sensitivity to moisture and/or oxygen, combined with variable amounts of incorporated solvent as a result of sample preparation precluded the acquisition of accurate elemental analysis results for **1**.

Compound **1** was also crystallized from $Et_2O$ as $K_2$[(NON$^{Ar}$)($Et_2O$)$_2$] (**1_{Et$_2$O}$_2$**) and in the presence of 18-c-6 as $K_2$[(NON$^{Ar}$)(18-c-6)] (**1_{18-c-6}**). The X-ray diffraction data was solved in each case and is presented in this publication for reference.

**Figure S1:** $^1$H NMR spectrum ($CD_3CN$, 600 MHz, 298 K) of $K_2[(NON^{Ar})(THF)]$ (**1_{THF}**)

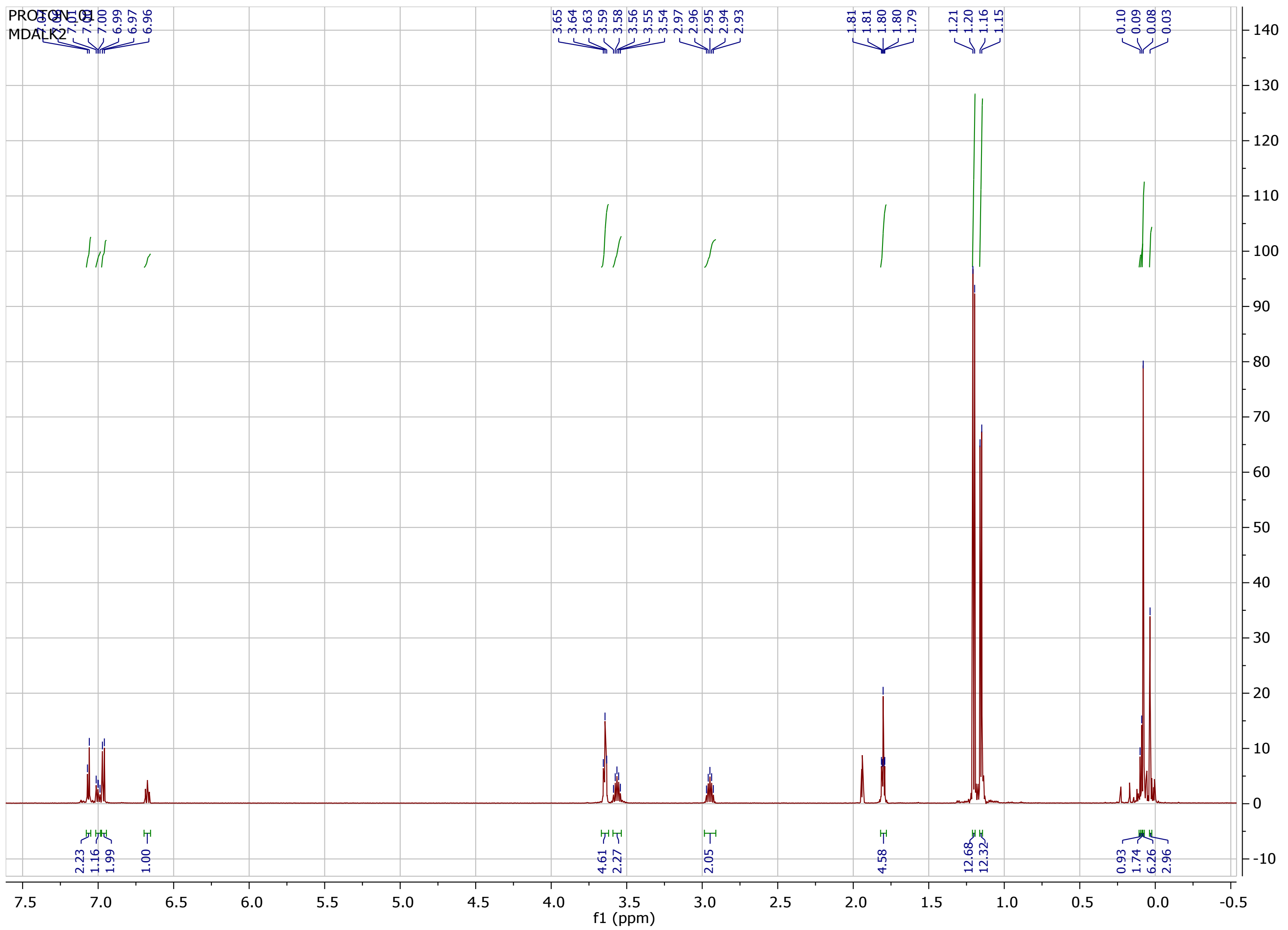

**Figure S2:** $^{13}C\{^{1}H\}$ NMR spectrum ($CD_3CN$, 151 MHz, 298 K) of $K_2[(NON^{Ar})(THF)]$ (**1_{THF}**)

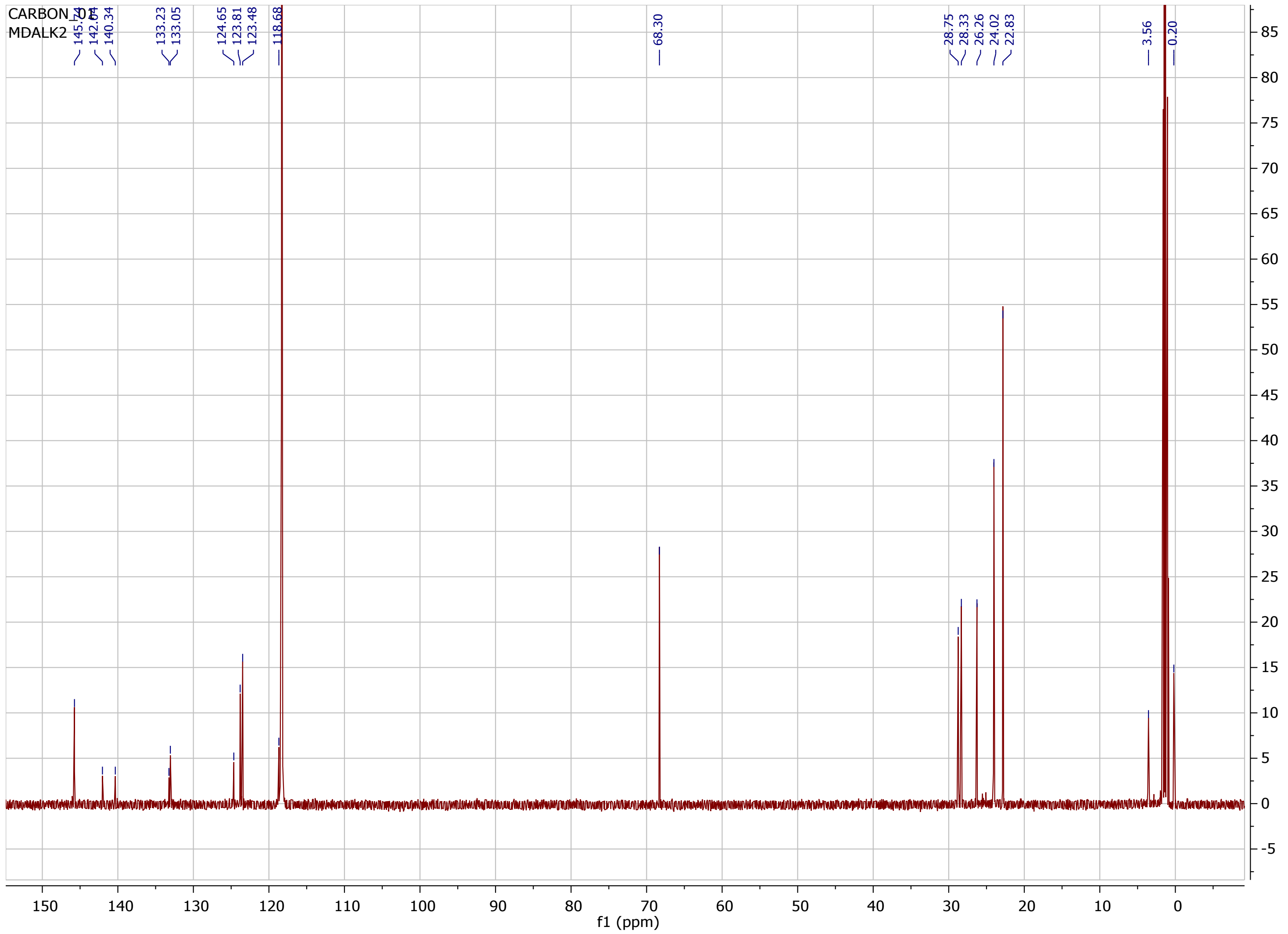

**Figure S3a:** ORTEP of the asymmetric unit of $[K_2(\{NON^{Ar}\}\{THF\}_3)]_2$ (**[1_{THF}$_3$]$_2$**) (ellipsoids 30%, hydrogen atoms omitted).

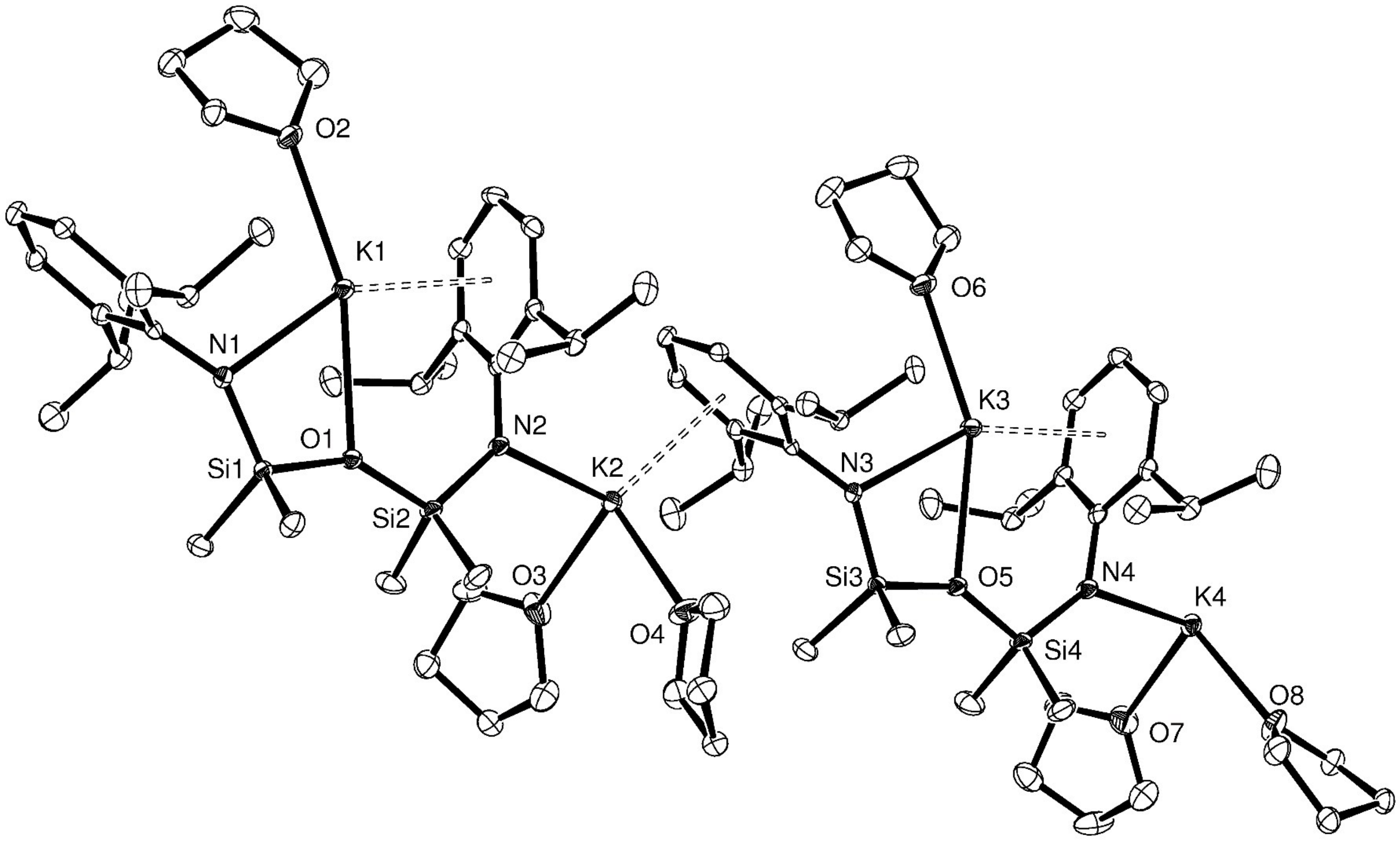

**Figure S3b:** ORTEP showing the connectivity of polymeric $K_2[(NON^{Ar})(THF)_3]$ (**1_{THF}$_3$**) (ellipsoids 30%, hydrogen atoms omitted).

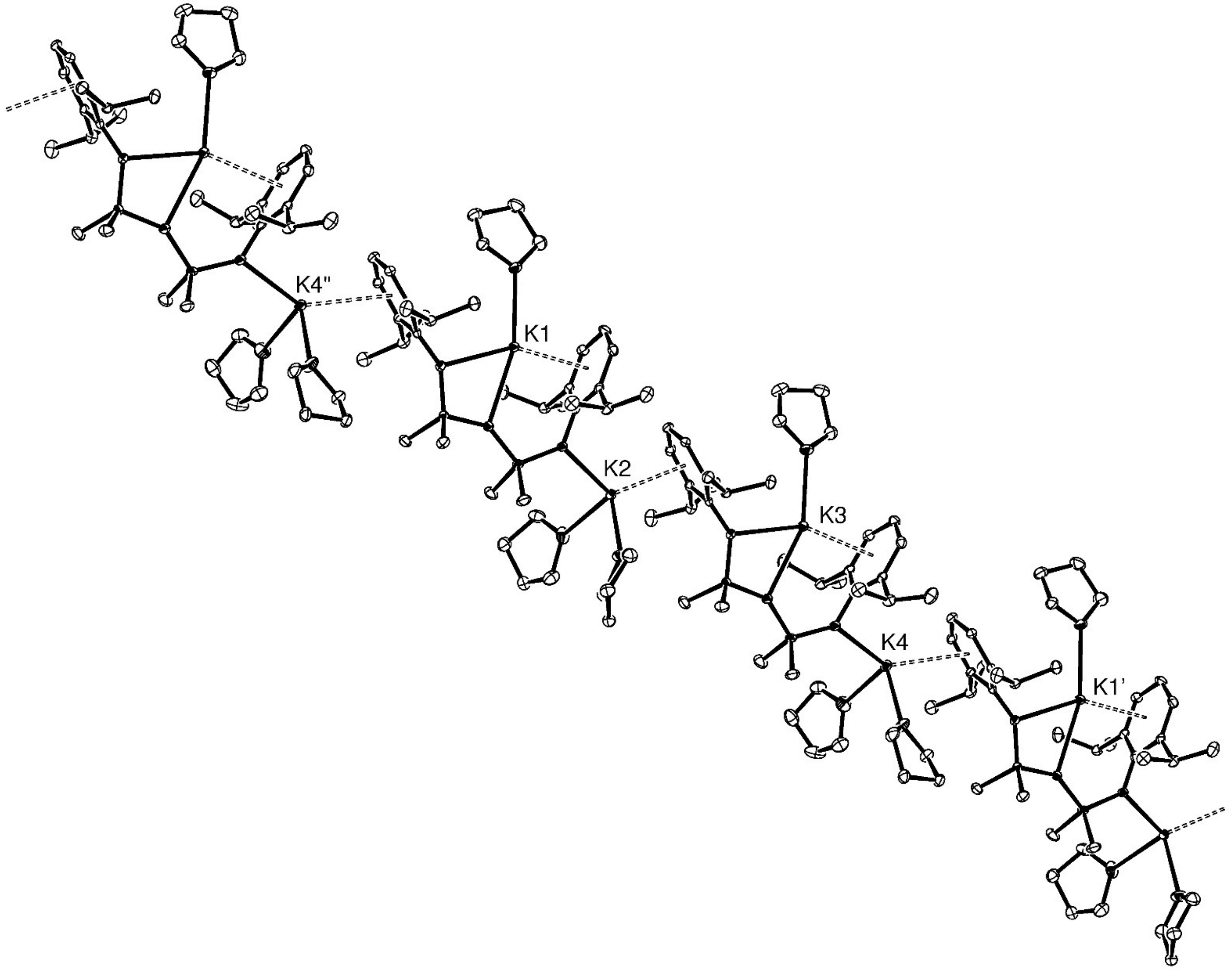

**Figure S4a:** ORTEP of the asymmetric unit of $K_2[(NON^{Ar})(Et_2O)_2]$ (**1_{Et₂O}₂**) (ellipsoids 30%, hydrogen atoms omitted).

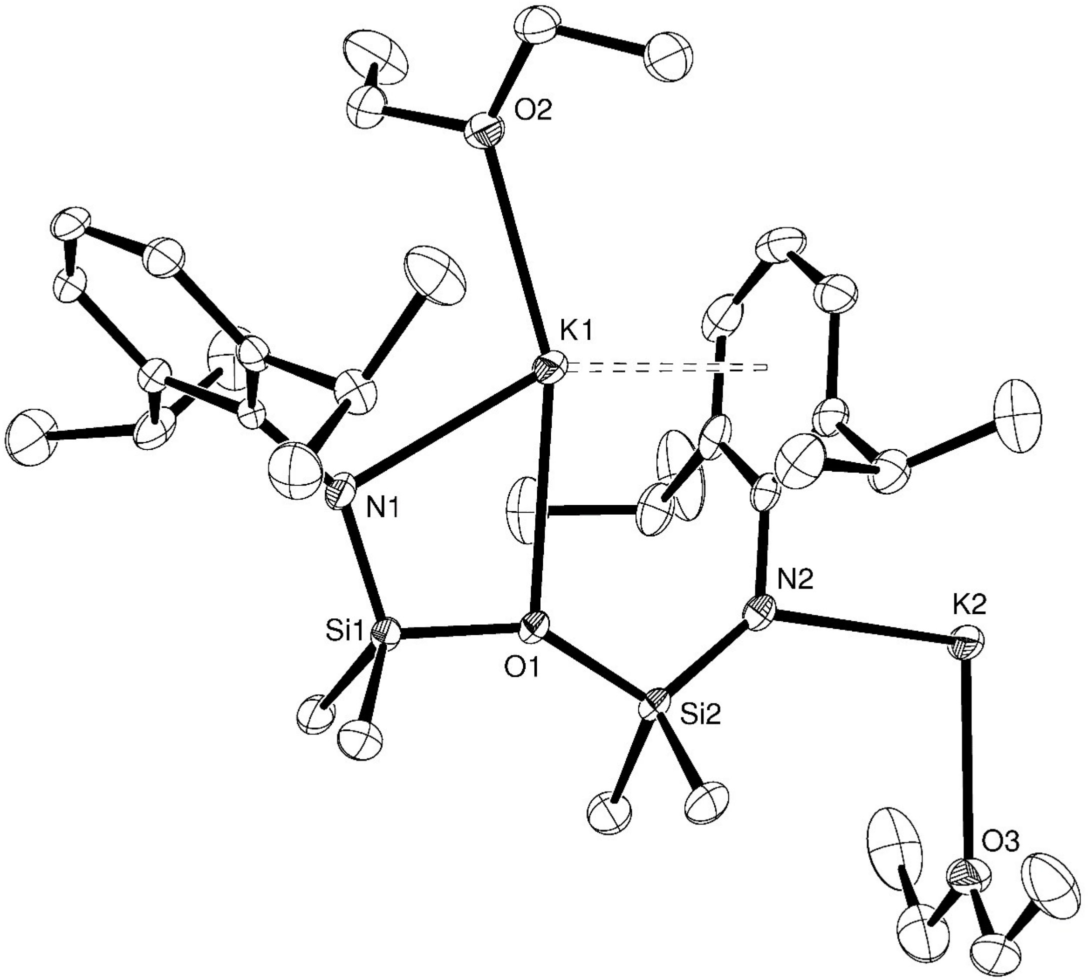

**Figure S4b:** ORTEP showing the connectivity of polymeric $K_2[(NON^{Ar})(Et_2O)_2]$ (**1_Et₂O**) (ellipsoids 30%, hydrogen atoms omitted).

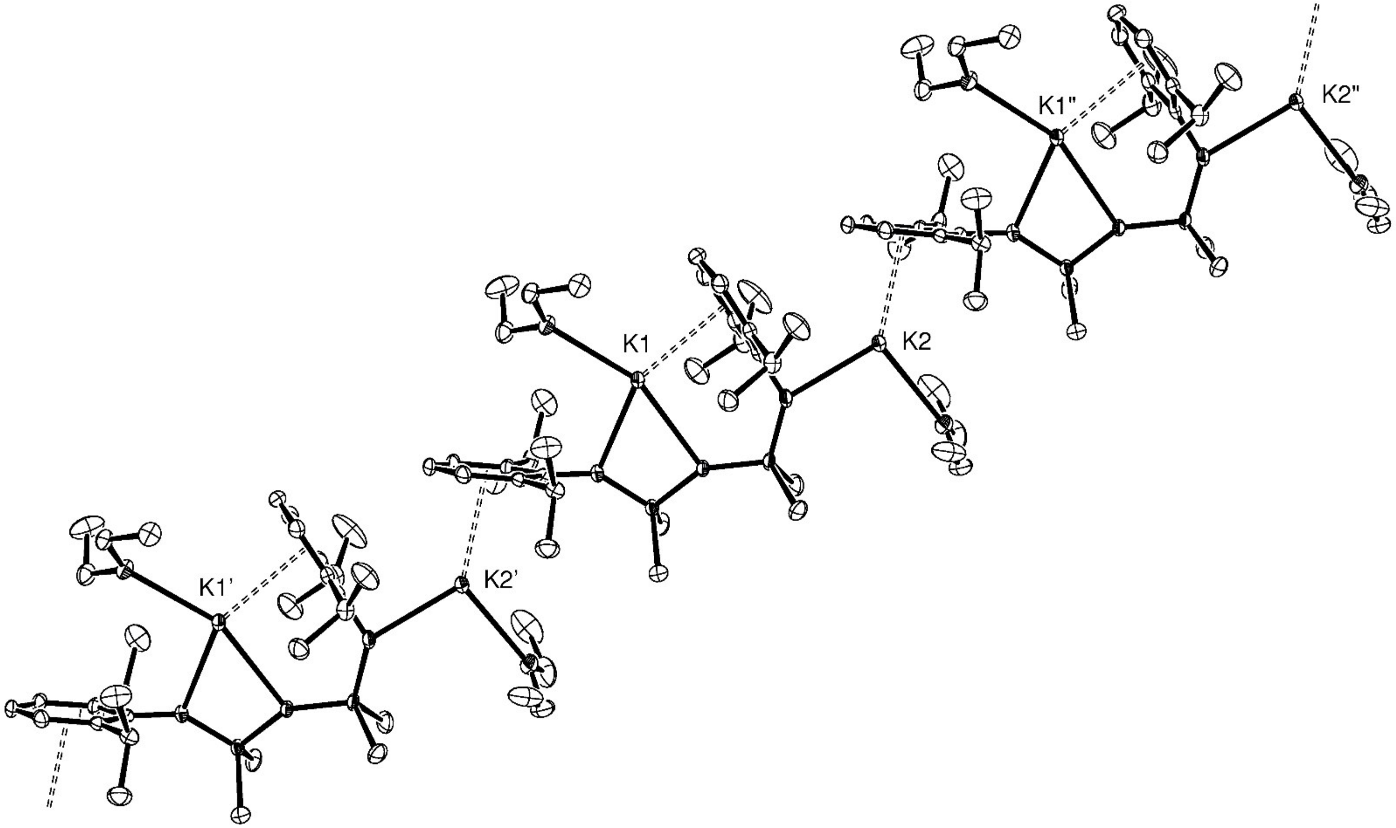

**Figure S5a:** ORTEP of the asymmetric unit of $K_2[(NON^{Ar})(18\text{-crown-6})]$ (**1_18-c-6**) (ellipsoids 30%, hydrogen atoms omitted).

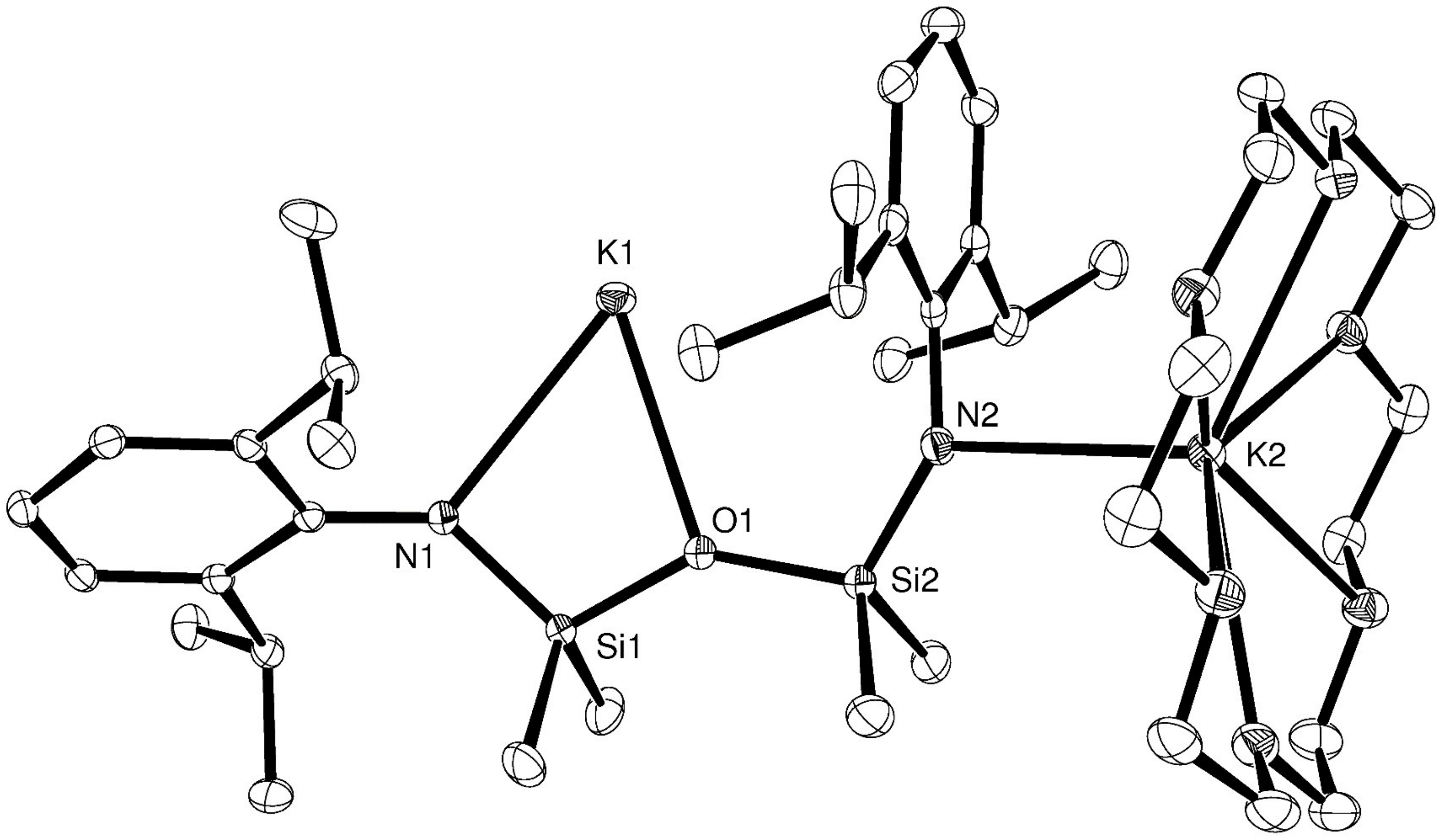

**Figure S5b:** ORTEP showing the connectivity of dimeric $K_2[(NON^{Ar})(18\text{-crown-6})]$ (**1_18-c-6**) (ellipsoids 30%, hydrogen atoms omitted).

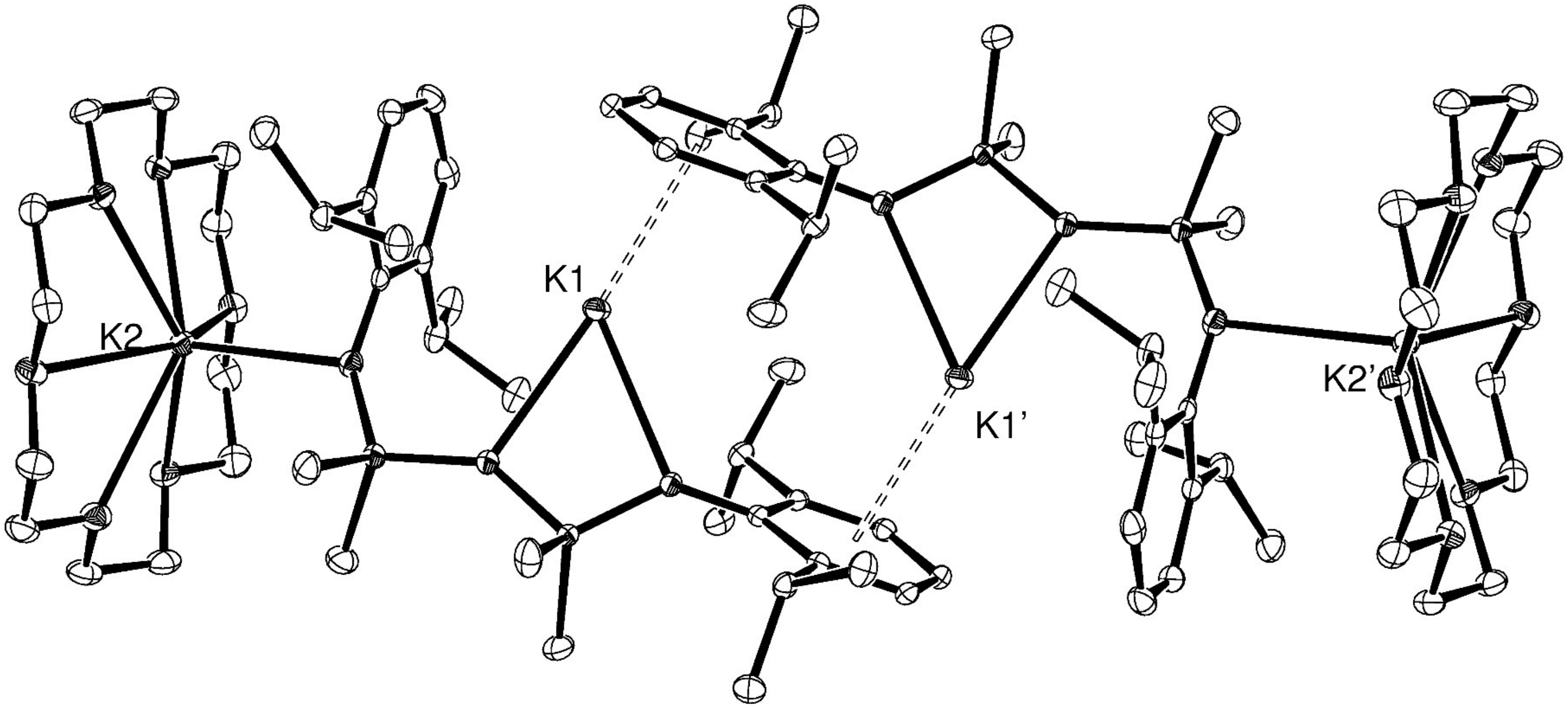

*Synthesis of K[In(NON$^{Ar}$)Cl$_2$] (**2**)*

A solution of $K_2$[(NON$^{Ar}$)(THF)] (**1_{THF}**) (0.500 g, 0.79 mmol) in $Et_2O$ was added to a suspension of $InCl_3$ (0.174 g, 0.79 mmol) in $Et_2O$ and stirred for 12 hours. The solution was filtered and solvent was removed *in vacuo* to give K[In(NON$^{Ar}$)Cl$_2$] as a colourless solid. Yield 0.430 g, 77 %..

Single crystals suitable for X–ray diffraction were grown from a saturated solution toluene solution stored at –30 °C overnight.

$^{1}$H NMR ($C_6D_6$, 300 MHz): $\delta$ 7.05 (d, $J$ = 7.5, 4H, $C_6$*$H_3$*), 6.90 (t, $J$ = 7.5, 2H, $C_6$*$H_3$*), 4.18 (sept, $J$ = 6.8, 4H, C*H*Me$_2$), 1.34 (d, $J$ = 6.8, 24H, CH*Me$_2$*), 0.42 (s, 12H, Si*Me$_2$*).

$^{13}$C{$^{1}$H} NMR ($C_6D_6$, 151 MHz): $\delta$ 148.5, 145.7, 128.4, 124.0, 123.38 (*$C_6$*$H_2$), 27.5 (*C*HMe$_2$), 25.5 (CH*Me$_2$*), 2.9 (Si*Me$_2$*).

Elemental Analysis: $C_{28}H_{46}Cl_2InKN_2OSi_2$. Calc: C, 47.52; H, 6.55; N, 3.96. Anal: C, 46.64; H, 6.32; N, 4.09.

The crystal structure of **2** was also obtained as the benzene solvate (**2_benzene**).

**Figure S6:** $^{1}$H NMR spectrum ($C_6D_6$, 300 MHz, 298 K) of K[In(NON$^{Ar}$)$Cl_2$] (**2**)

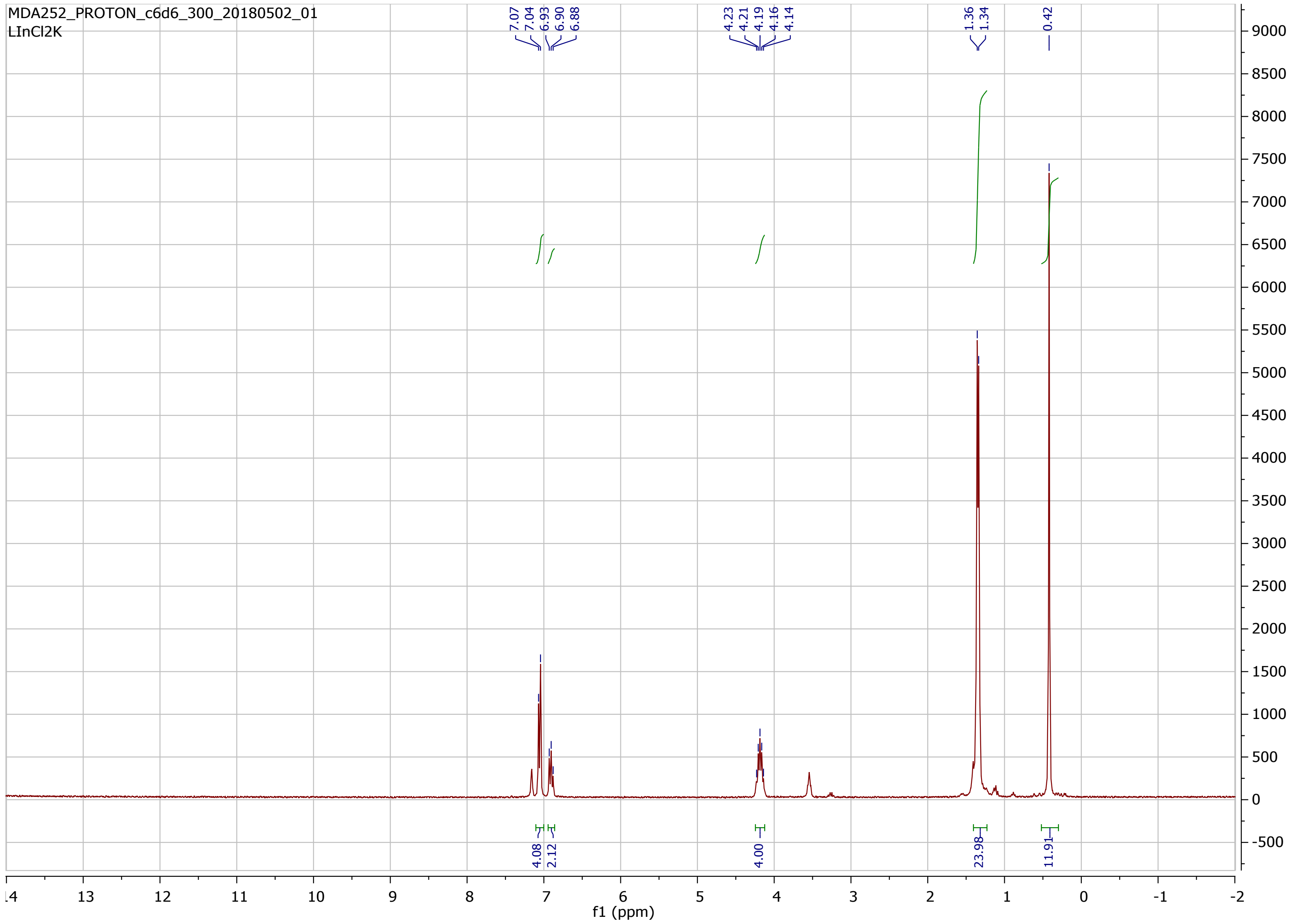

**Figure S7:** $^{13}C\{^1H\}$ NMR spectrum ($C_6D_6$, 151 MHz, 298 K) of $K[In(NON^{Ar})Cl_2]$ (**2**)

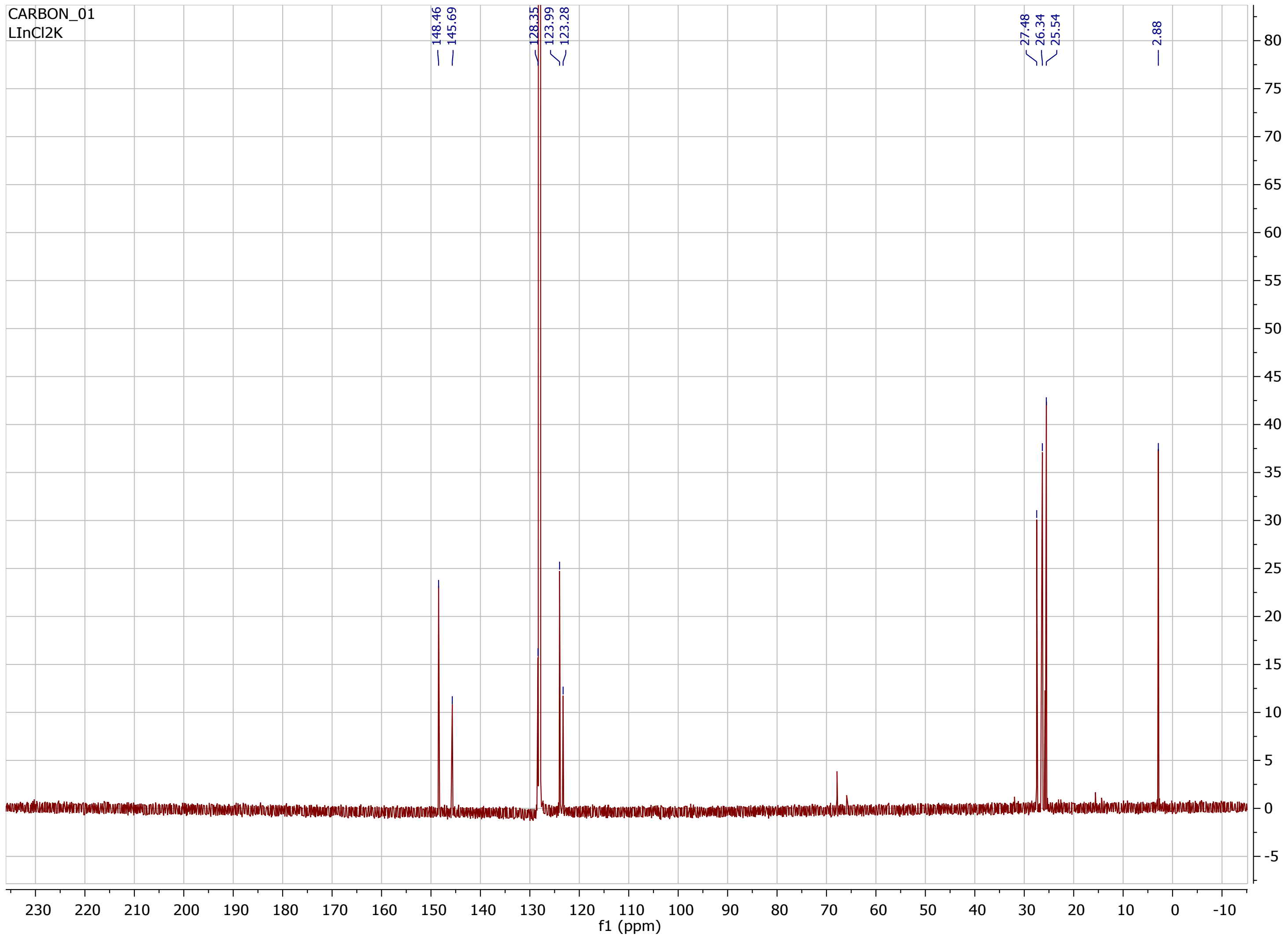

**Figure S8a:** ORTEP of the asymmetric unit of K[In(NON$^{Ar}$)$Cl_2$] (**2**) (ellipsoids 30%, hydrogen atoms omitted).

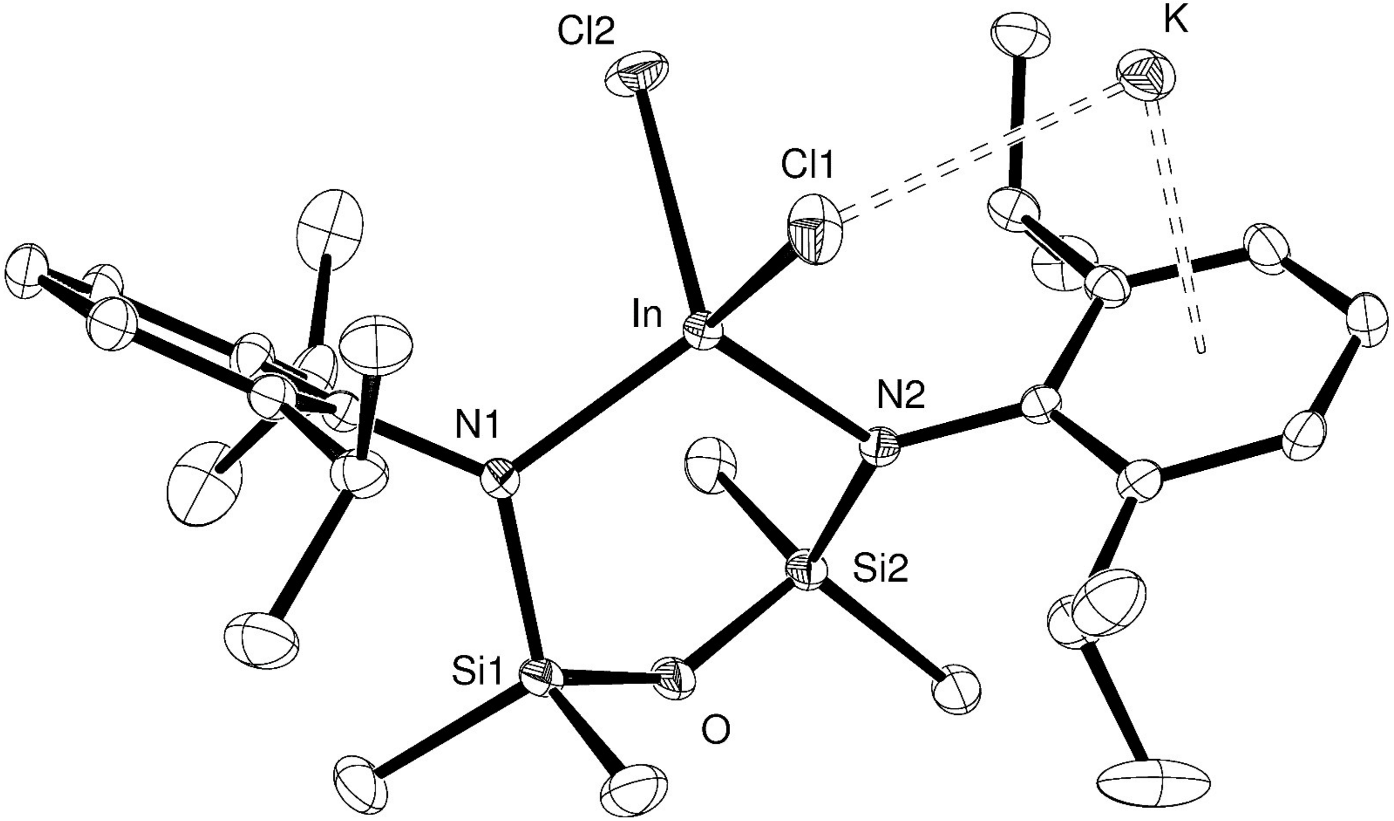

**Figure S8b:** ORTEP showing the connectivity of polymeric K[In(NON$^{Ar}$)Cl$_2$] (**2**) (ellipsoids 30%, hydrogen atoms omitted).

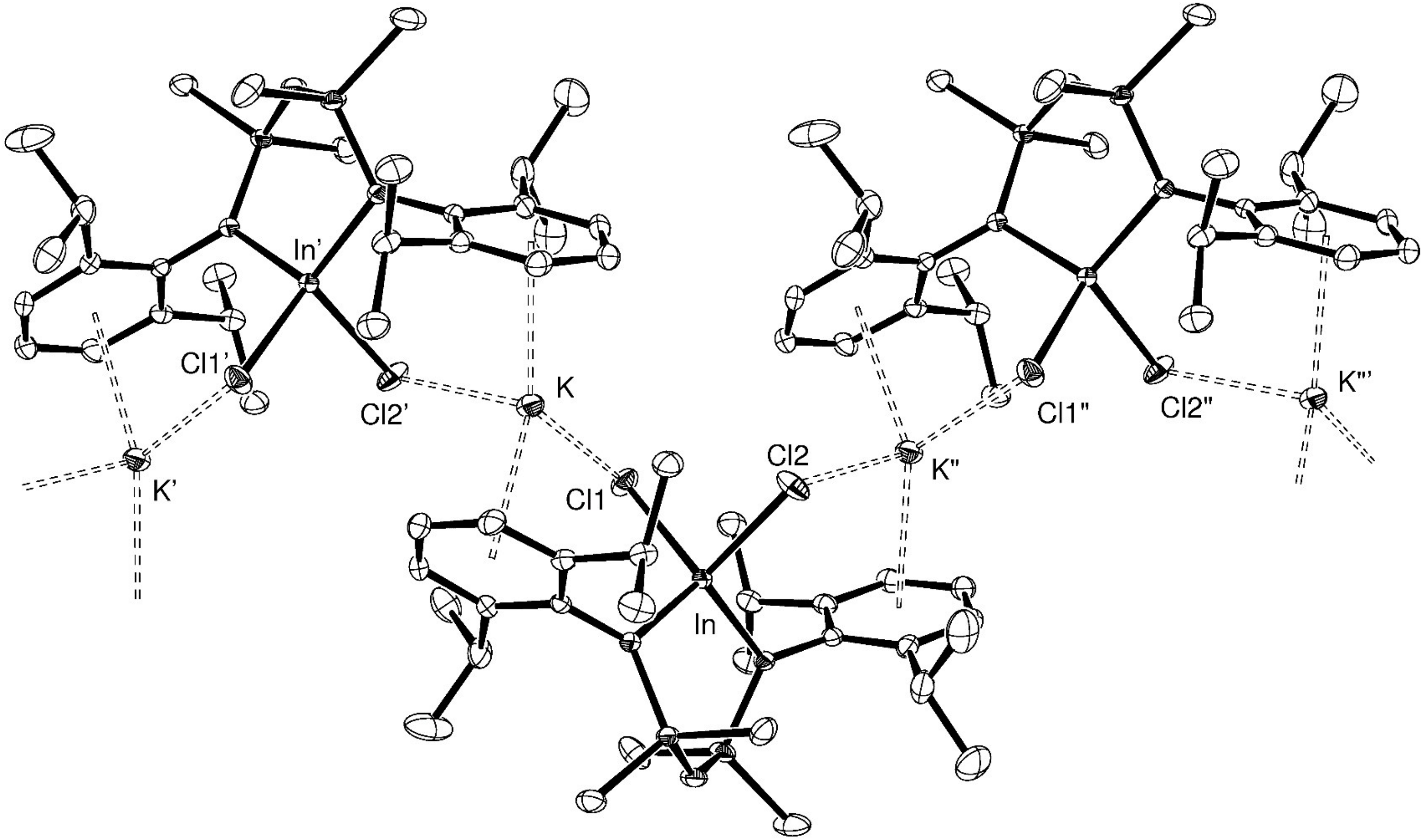

**Figure S9:** ORTEP of K[In(NON$^{Ar}$)Cl$_2$]·benzene (**2_benzene**) (ellipsoids 30%, hydrogen atoms omitted).

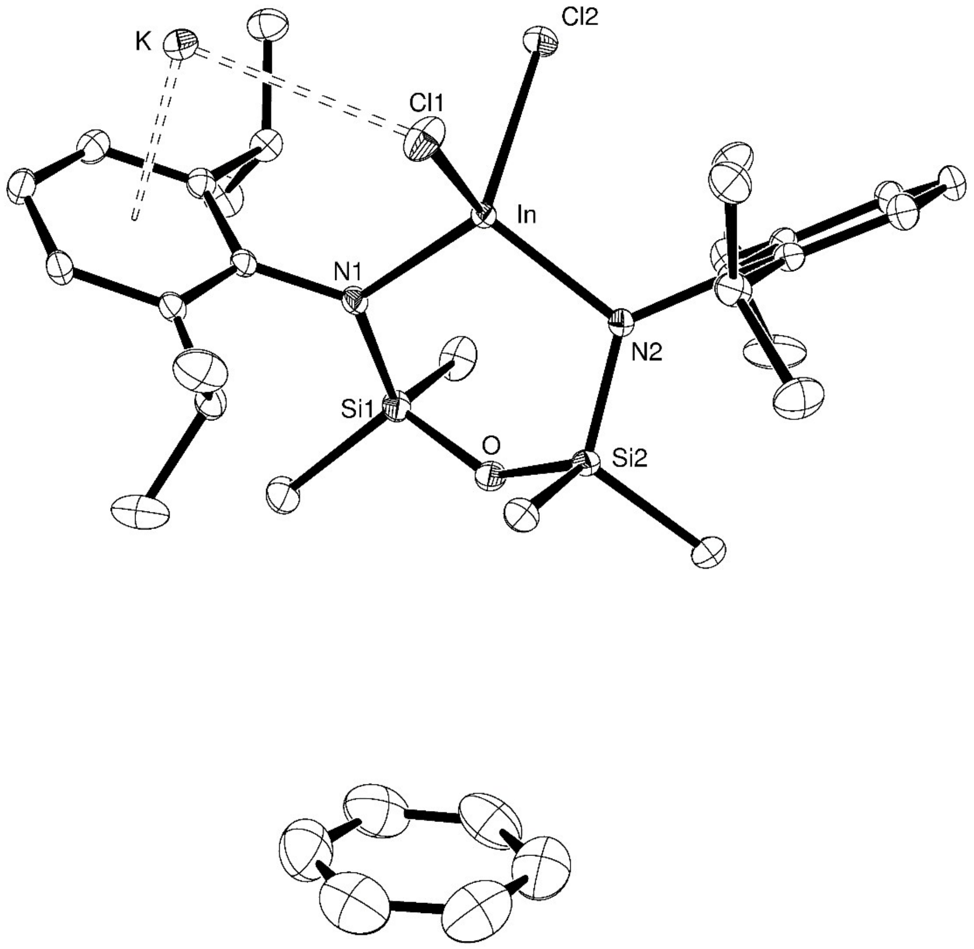

*Synthesis of K[In(NON$^{Ar}$)] (**3**)*

A solution of **2** (0.400 g, 0.56 mmol) in $Et_2O$ was added to a Schlenk with a potassium mirror (0.044 g, 1.13 mmol) and was stirred at room temperature of 3 days. The solvent was removed *in vacuo*, and the remaining products was redissolved in hexane and filtered. Removal of solvent in *vacuo* gave **3** as a yellow microcrystalline solid. Yield 0.627 g, 74 %.

Single crystals suitable for X–ray diffraction were grown from a saturated solution of **3** in hexane stored at –30 °C overnight.

$^1$H NMR ($C_6D_6$, 300 MHz): $\delta$ 6.88 (d, *J* = 7.5, 4H, $C_6H_3$), 6.58 (t, *J* = 7.5, 2H, $C_6H_3$), 4.16 (sept, *J* = 6.8, 4H, C*H*Me$_2$), 1.26 (d, *J* = 6.8, 12H, CH*Me$_2$*), 1.01 (d, *J* = 6.8, 12H, CH*Me$_2$*), 0.40 (s, 12H, Si*Me$_2$*).

$^{13}$C{$^1$H} NMR ($C_6D_6$, 151 MHz): $\delta$ 152.3, 147.6, 123.0, 119.9 (*C$_6$*H$_3$), 27.0 (*C*HMe$_2$), 23.9 (CH*Me$_2$*), 4.0 (Si*Me$_2$*).

Extreme sensitivity to moisture and/or oxygen, combined with variable amounts of incorporated solvent as a result of sample preparation precluded the acquisition of accurate elemental analysis results for **3**.

**Figure S10:** $^{1}$H NMR spectrum ($C_6D_6$, 300 MHz, 298 K) of K[In(NON$^{Ar}$)] (**3**)

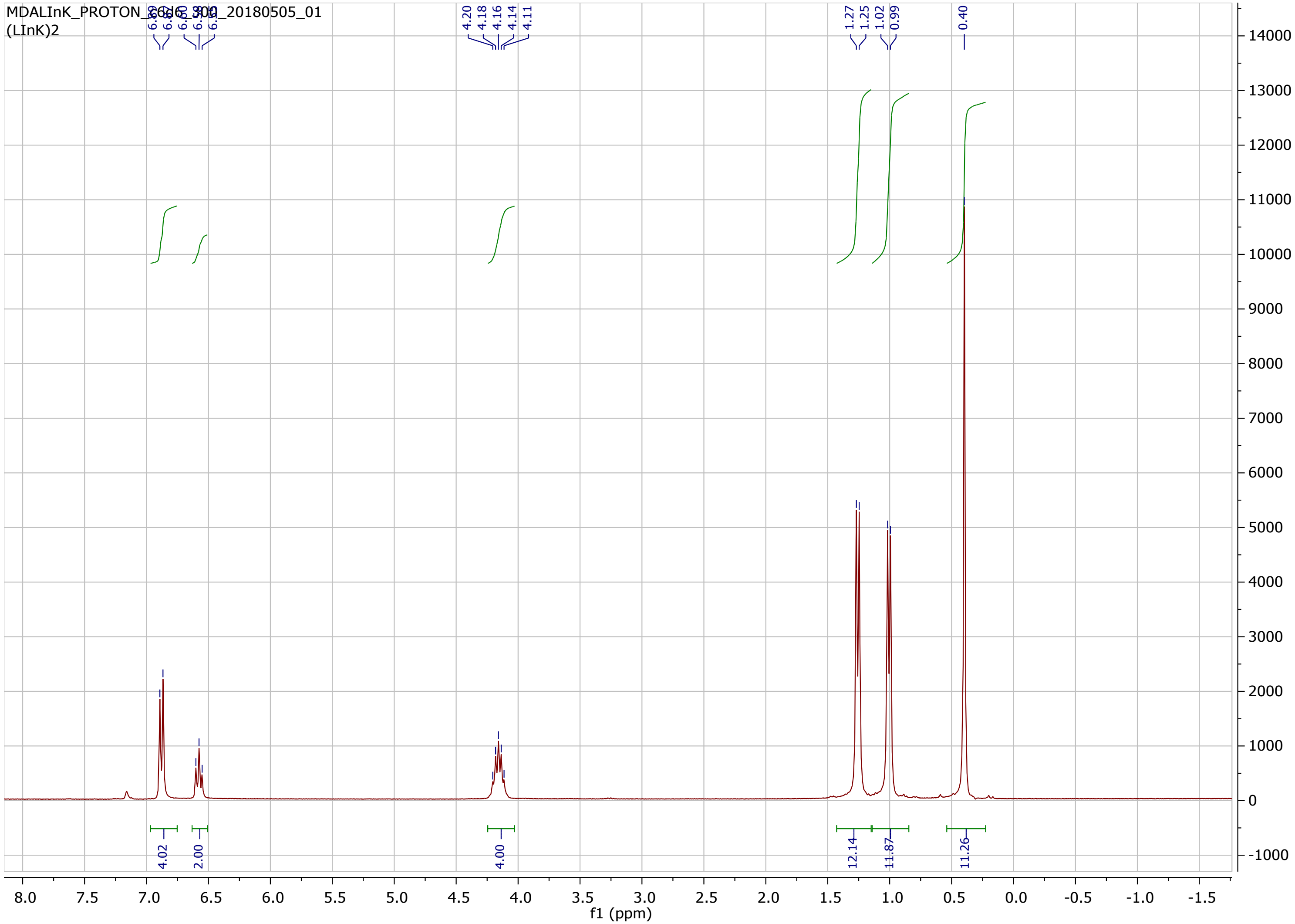

**Figure S11:** $^{13}C\{^1H\}$ NMR spectrum ($C_6D_6$, 151 MHz, 298 K) of K[In(NON$^{Ar}$)] (**3**)

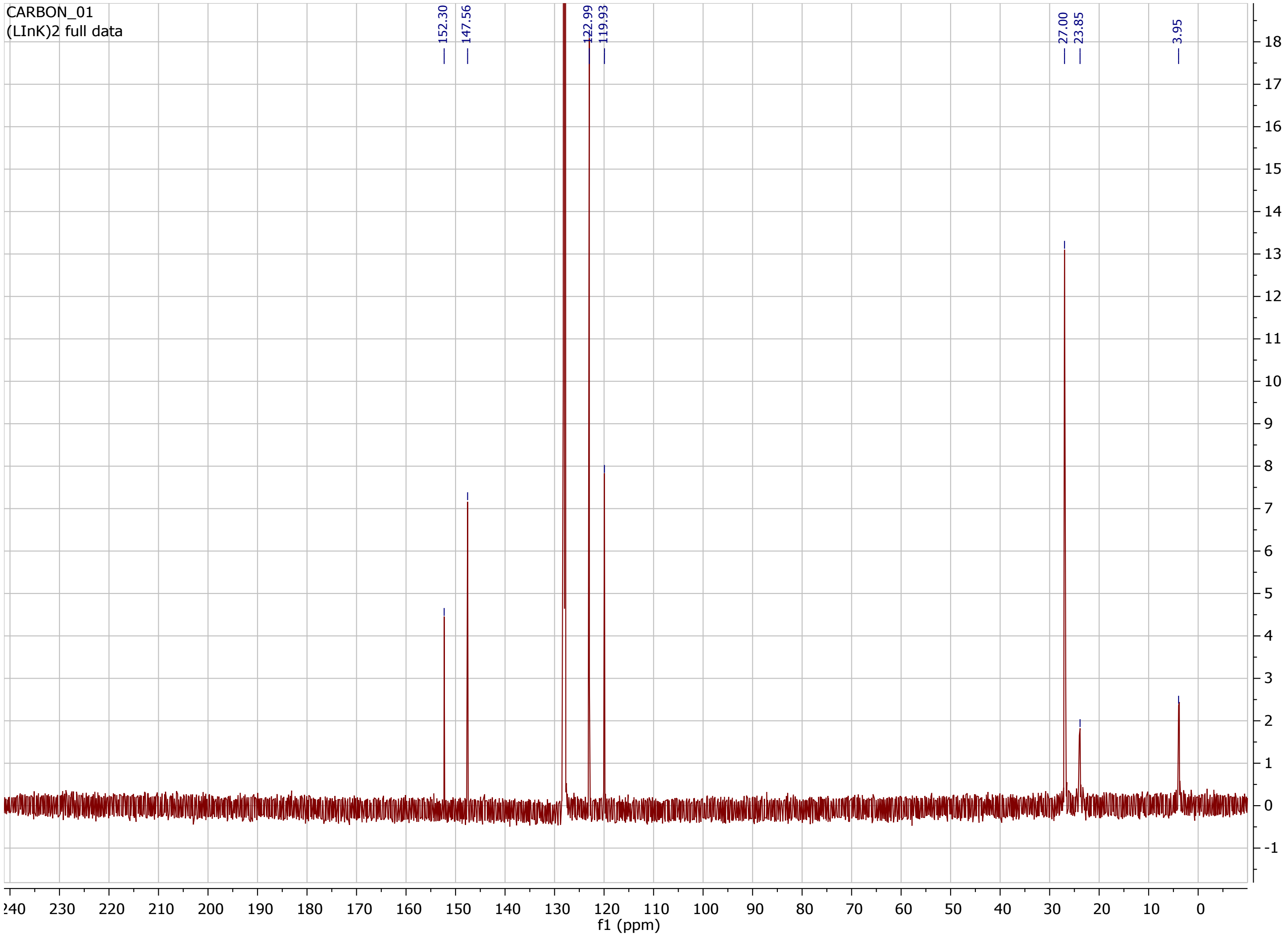

**Figure S12:** ORTEP of [K(In{NON$^{Ar}$})]$_2$ ([**3**]$_2$) (ellipsoids 30%, hydrogen atoms and hexane solvate omitted).

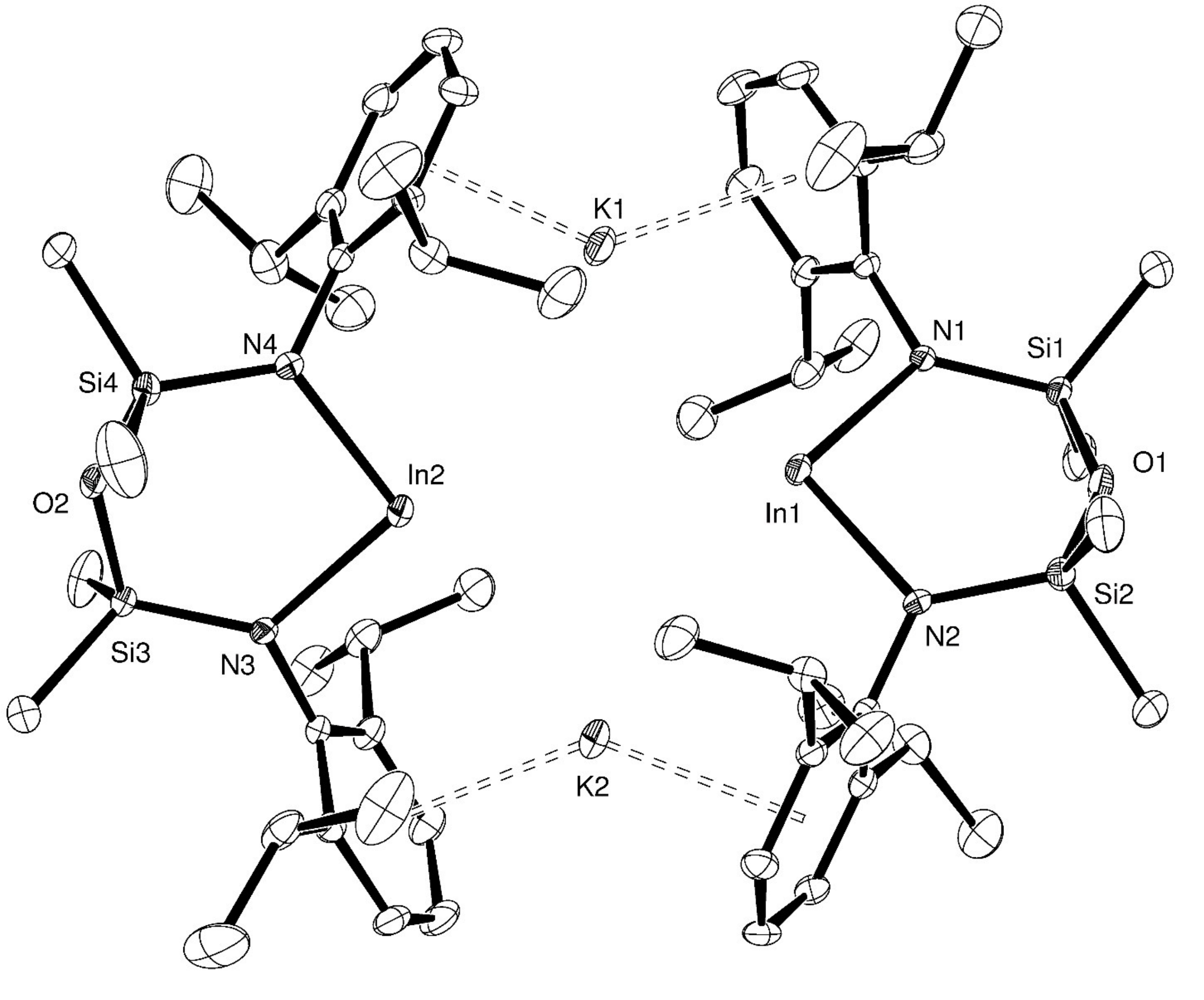

*Synthesis of K[In(NON$^{Ar}$)(N(H){2-(CPh$_2$)-6-(CHPh$_2$)-4-tBuC$_6$H$_2$}] (**4**)*

A solution of Ar$^‡$N$_3$ (Ar$^‡$ = 2,6-(CHPh$_2$)$_2$-4-*t*BuC$_6$H$_2$, 0.071 g, 0.16 mmol) in hexane was added dropwise to a solution of **3** (0.100 g, 0.16 mmol) in hexane at –78 °C and then allowed to warm to room temperature and stirred for 1 hour. The solution was concentrated *in vacuo* and stored at –30 °C overnight to afford a colourless crystalline solid **4**. Yield 0.150 g, 88 %.

Single crystals suitable for X–ray diffraction were grown from a saturated solution of **4** in toluene and stored at –30 °C overnight.

$^1$H NMR (C$_6$D$_6$, 600 MHz): *δ* 7.18 (m, 4H, Ar-*H*), 7.13 (m, 6H, Ar-*H*,), 7.12 – 7.07 (m, 3H, Ar-*H*), 7.03 (m, 2H, Ar-*H*), 7.01 – 6.98 (m, 3H, Ar-*H*,), 6.91 (m, 2H, Ar-*H*), 6.83 (s, 4H, Ar-*H*), 6.81 – 6.72 (m, 3H, Ar-*H*), 6.67 (s, 2H, Ar-*H*), 5.56 (s (br), 1H, C*H*Ph$_2$), 4.18 (m (br), 4H, C*H*Me$_2$), 3.24 (s, 1H, N*H*), 1.48 (d, *J* = 6.7, 6H, CH*Me$_2$*), 1.38 (d, *J* = 6.7, 6H, CH*Me$_2$*), 1.03 (s, 9H, C*Me$_3$*), 0.91 (d (br), 6H, CH*Me$_2$*), 0.77 (d, *J* = 6.7, 6H, CH*Me$_2$*), 0.58 (s, 6H, Si*Me$_2$*), 0.45 (s, 6H, Si*Me$_2$*).

$^{13}$C NMR (C$_6$D$_6$, 151 MHz): *δ* 151.3, 149.8, 146.7, 144.4, 143.6, 142.2, 137.9, 135.7, 130.0, 129.3, 128.7, 128.6, 128.6, 127.4, 127.3, 126.8, 126.7, 125.7, 124.5, 124.1, 123.6, 122.3 (Ar-*C*), 54.9 (*C*HPh$_2$), 53.6 (*C*HPh$_2$), 34.1 (*C*Me$_3$), 31.8, 31.1, (*C*HMe$_2$), 27.8, 27.7, 26.5, 25.6, (CH*Me$_2$*), 21.5 (C*Me$_3$*) 4.75, 3.13 (Si*Me*$_3$).

Extreme sensitivity to moisture and/or oxygen, combined with variable amounts of incorporated solvent as a result of sample preparation precluded the acquisition of accurate elemental analysis results for **4**.

**Figure S13:** $^{1}$H NMR spectrum ($C_6D_6$, 600 MHz, 298 K) of K[In(NON$^{Ar}$)(N(H){2-($CPh_2$)-6-($CHPh_2$)-4-*t*Bu$C_6H_2$}] (**4**)

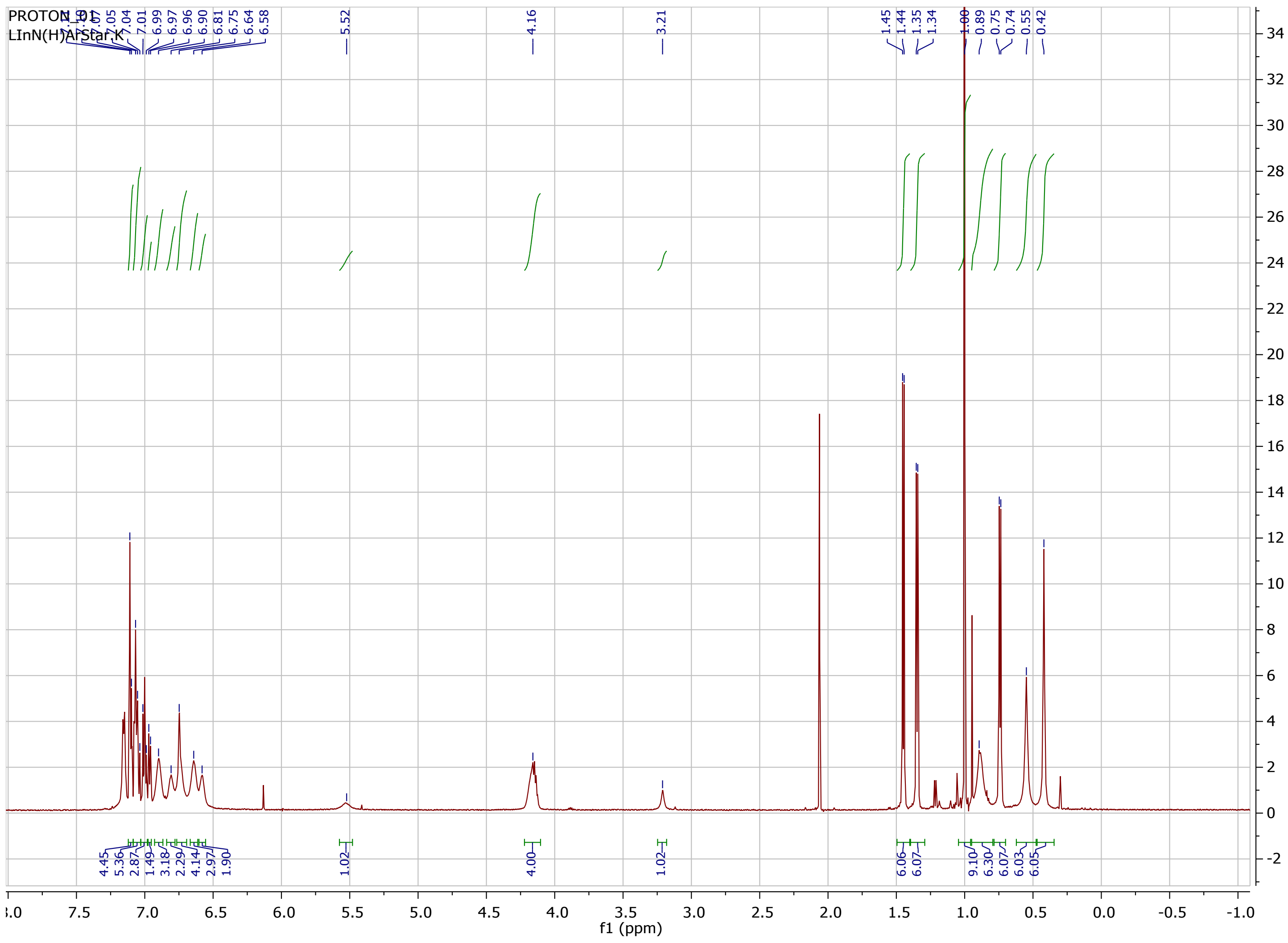

**Figure S14:** $^{13}C\{^{1}H\}$ NMR spectrum ($C_6D_6$, 151 MHz, 298 K) of K[In(NON$^{Ar}$)(N(H){2-(CPh$_2$)-6-(CHPh$_2$)-4-*t*BuC$_6$H$_2$}] (**4**)

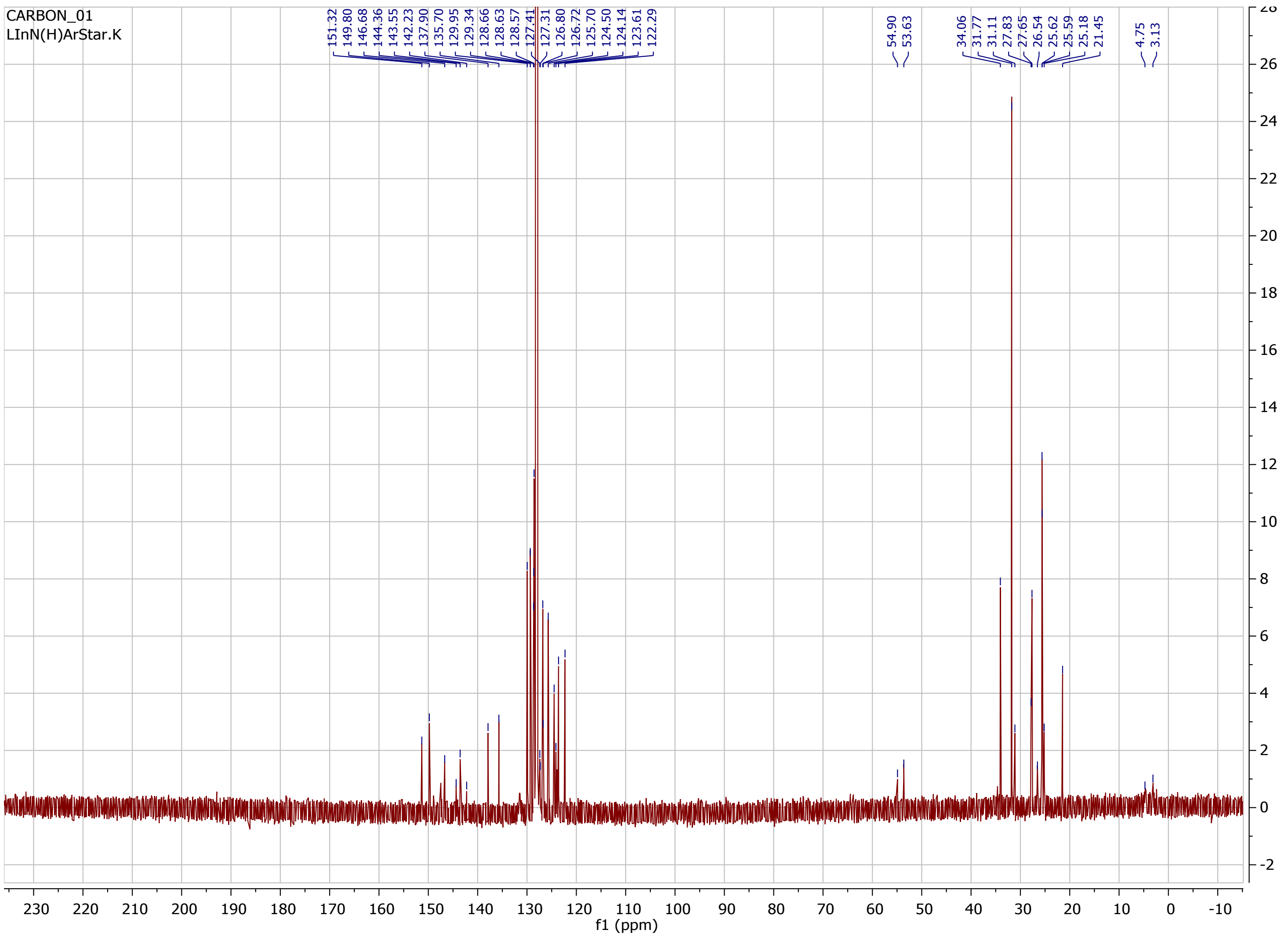

**Figure S15:** HSQC spectrum ($C_6D_6$, 151 MHz, 298 K) of K[In(NON$^{Ar}$)(N(H){2-(CPh$_2$)-6-(CHPh$_2$)-4-*t*BuC$_6$H$_2$}] (**4**)

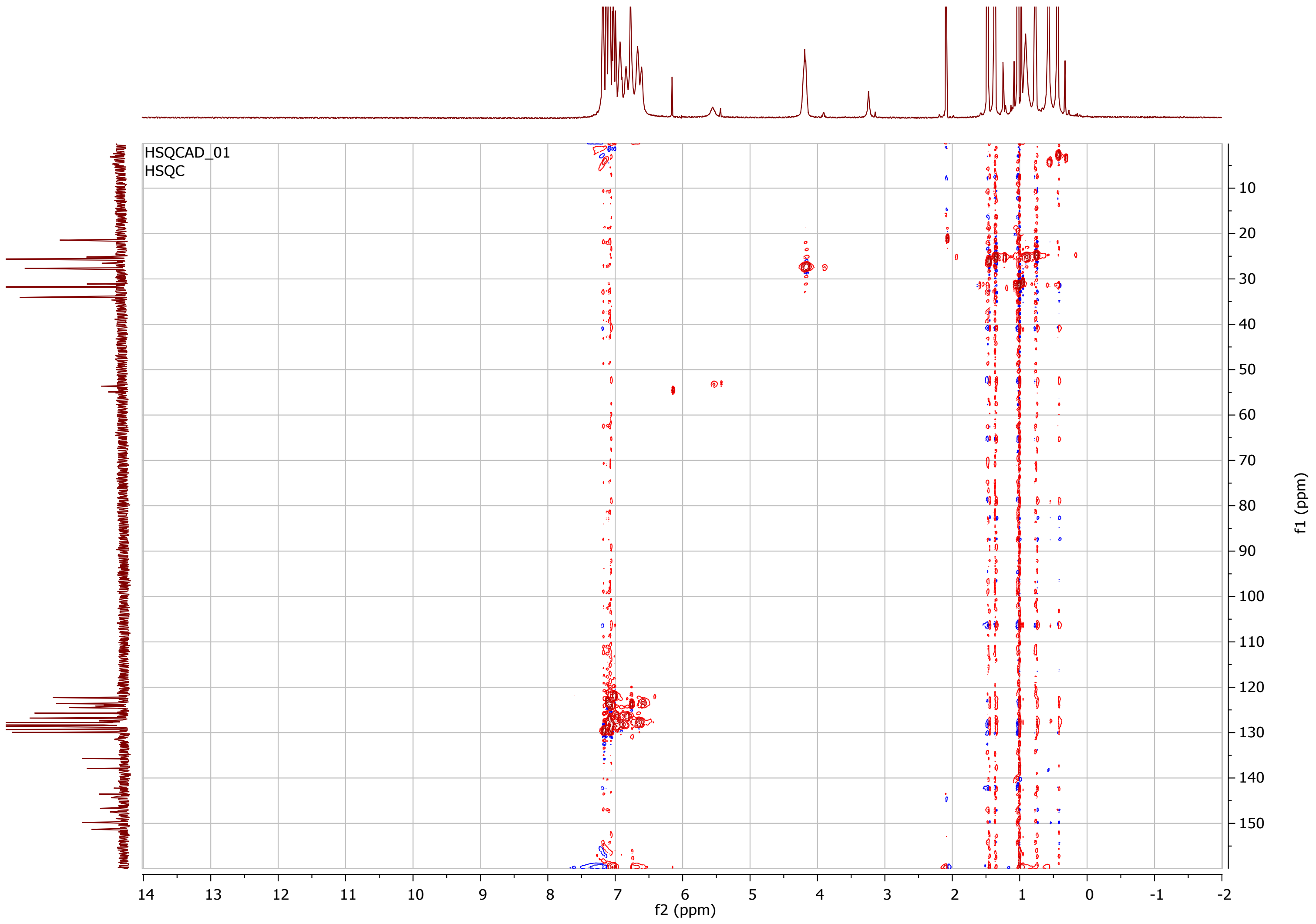

**Figure S16:** ORTEP of K[In(NON$^{Ar}$)(N(H){2-(CPh$_2$)-6-(CHPh$_2$)-4-*t*BuC$_6$H$_2$}] (**4**) (ellipsoids 30%, hydrogen atoms except N*H* and toluene solvate omitted).

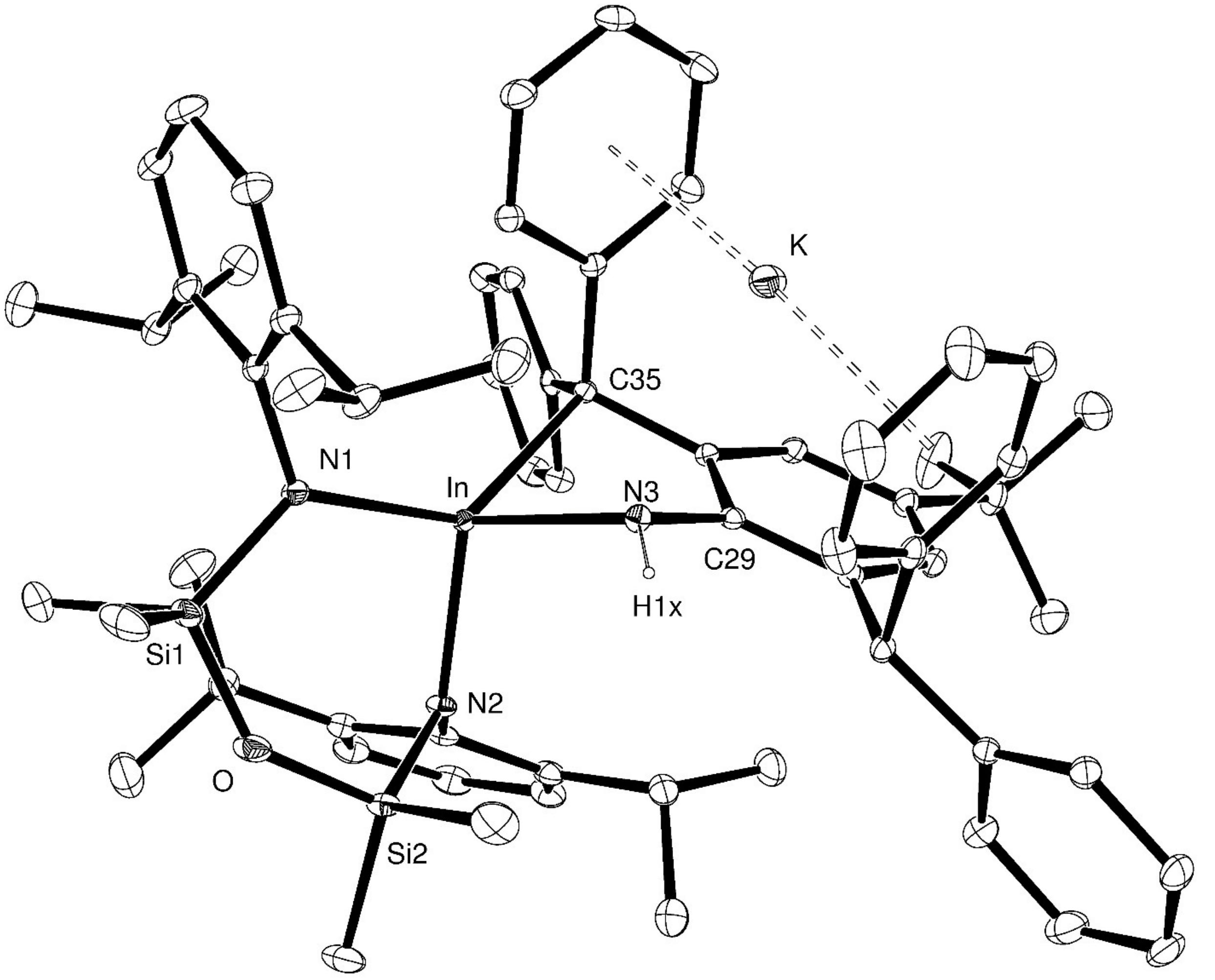

*Synthesis of K[In(NON$^{Ar}$)(NMes)] (**5**)*

A solution of mesityl azide (0.027 g, 0.16 mmol) in hexane was added dropwise to a solution of **3** (0.100 g, 0.16 mmol) in hexane at –78 °C then allowed to warm to room temperature and stirred for 1 hour. The solution was concentrated *in vacuo* and stored at –30 °C overnight to afford a yellow orange crystalline solid **5**. Yield 0.110 g, 89 %.

Single crystals suitable for X–ray diffraction were grown from a saturated solution of **5** in hexane and stored at –30 °C overnight.

$^{1}$H NMR ($C_6D_6$, 600 MHz): $\delta$ 6.95 (s (br), 6H, $C_6H_3$), 6.46 (s, 2H, Mes-$C_6H_2$), 4.01 (m (br), 4H, C*H*Me$_2$), 2.04 (s, 3H, Mes-4-*Me*$C_6H_2$), 1.57 (s, 6H, Mes-2,6-$Me_2C_6H_2$), 1.25 (d, *J* = 6.8, 12H, CH$Me_2$), 1.07 (s (br), 12H, CH$Me_2$), 0.33 (s, 12H, Si$Me_2$).

$^{13}$C{$^{1}$H} NMR ($C_6D_6$, 151 MHz): $\delta$ 163.9, 146.9, 129.4, 127.5, 123.6, 123.3, 118.7 (Ar-$C_6H_3$ and Mes-$C_6H_2$), 32.0 (Mes-4-*Me*$C_6H_2$), 27.8 (*C*HMe$_2$), 24.2 (CH$Me_2$), 20.8 (CH$Me_2$), 20.4 (Mes-2,6-$Me_2C_6H_2$), 2.5 (Si$Me_2$).

Extreme sensitivity to moisture and/or oxygen, combined with variable amounts of incorporated solvent as a result of sample preparation precluded the acquisition of accurate elemental analysis results for **5**.

*Synthesis of [K(crypt-222)][In(NON$^{Ar}$)(NMes)] (**6**)*

One equivalent of 222-cryptand (0.014 g, 0.038 mmol) dissolved in $C_6D_6$ was added to a J Young's tap NMR tube containing $C_6D_6$ solution of **5** (0.014 g, 0.038 mmol). Addition of the 222-cryptand solution led to incipient crystallisation of **6** as red plates. Unfortunately, the salt is highly insoluble once crystallized and we were unable to get sufficient quantities back into solution to obtain any spectroscopic data.

**Figure S17:** $^{1}$H NMR spectrum ($C_6D_6$, 600 MHz, 298 K) of K[In(NON$^{Ar}$)(NMes)] (**5**)

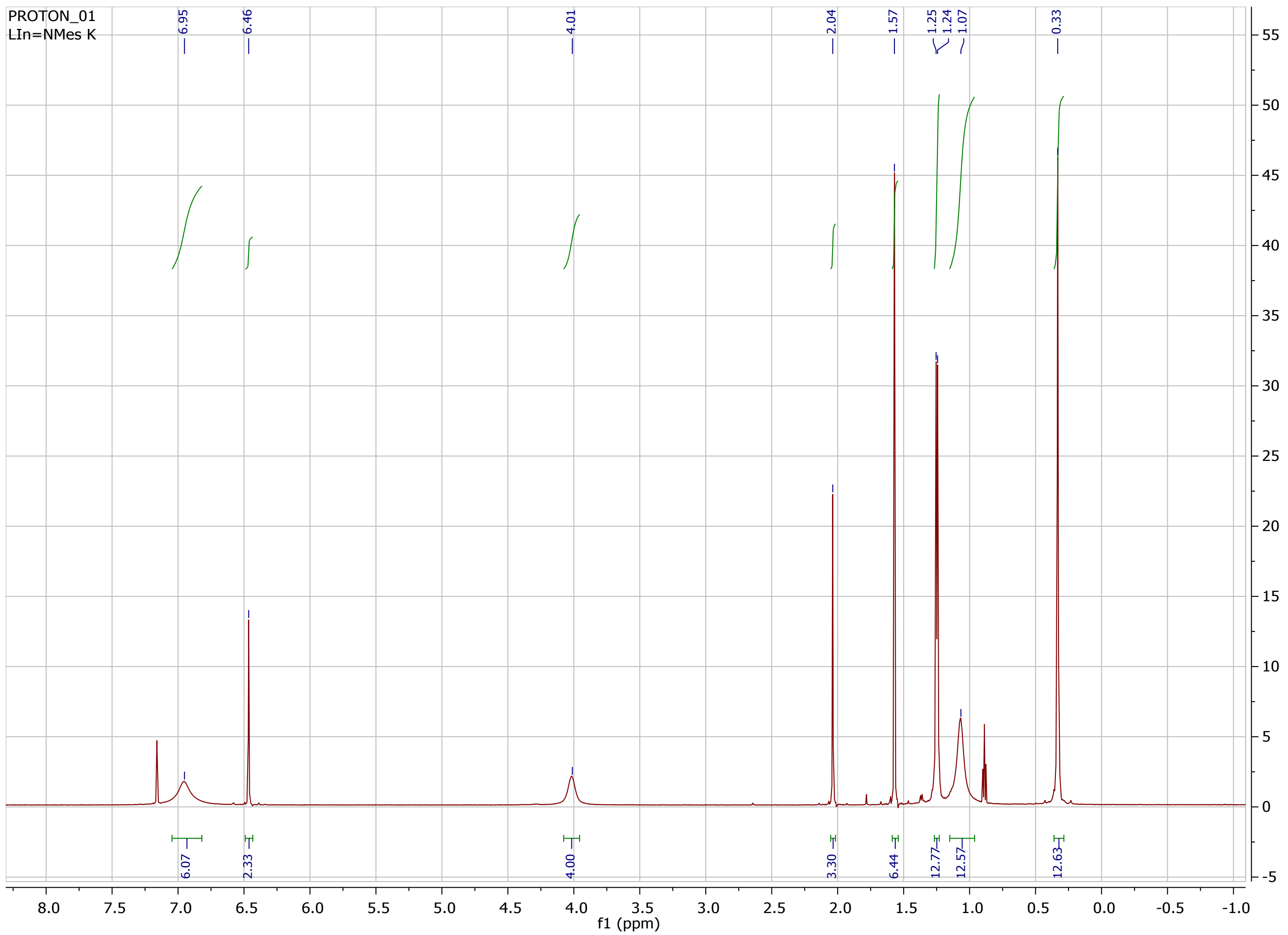

**Figure S18:** $^{13}C\{^1H\}$ NMR spectrum ($C_6D_6$, 151 MHz, 298 K) of K[In(NON$^{Ar}$)(NMes)] (**5**)

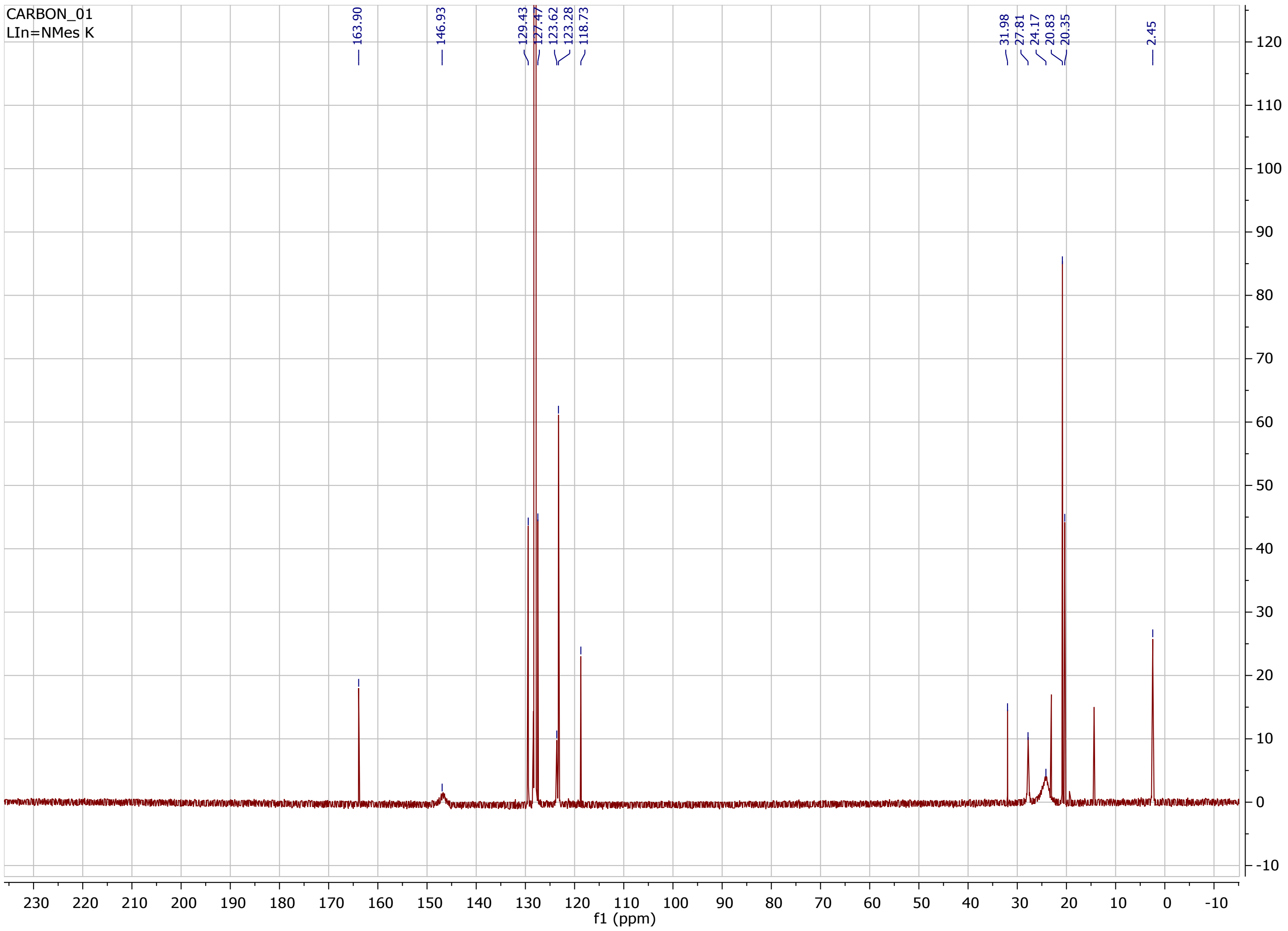

**Figure S19:** ORTEP of [K(In{NON$^{Ar}$}{NMes})]$_2$ ([**5**]$_2$) (ellipsoids 30%, hydrogen atoms and toluene solvate (x4) omitted).

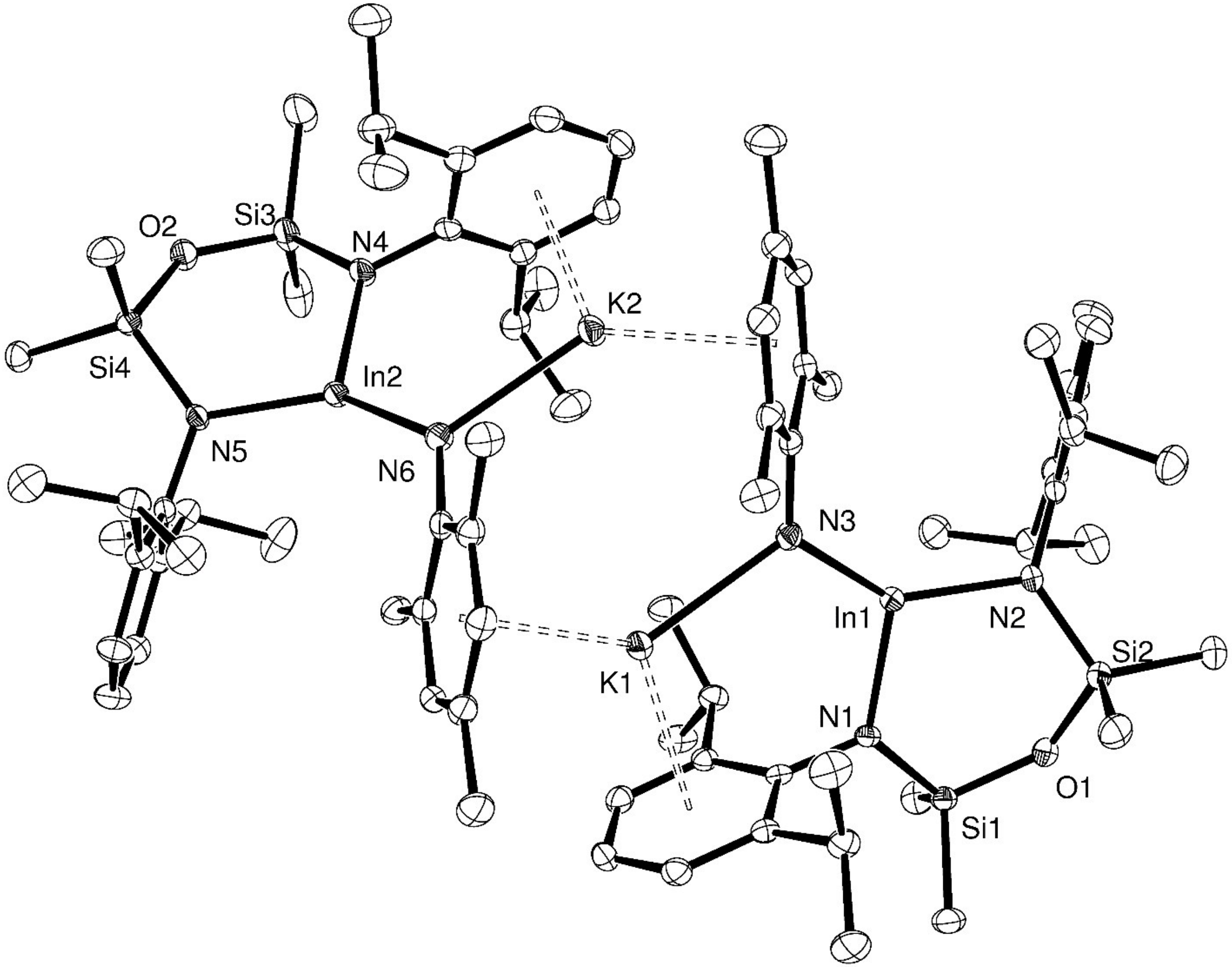

**Figure S20:** ORTEP of [K(crypt-222)][In(NON$^{Ar}$)(NMes)] (**6**) (ellipsoids 30%, hydrogen atoms omitted).

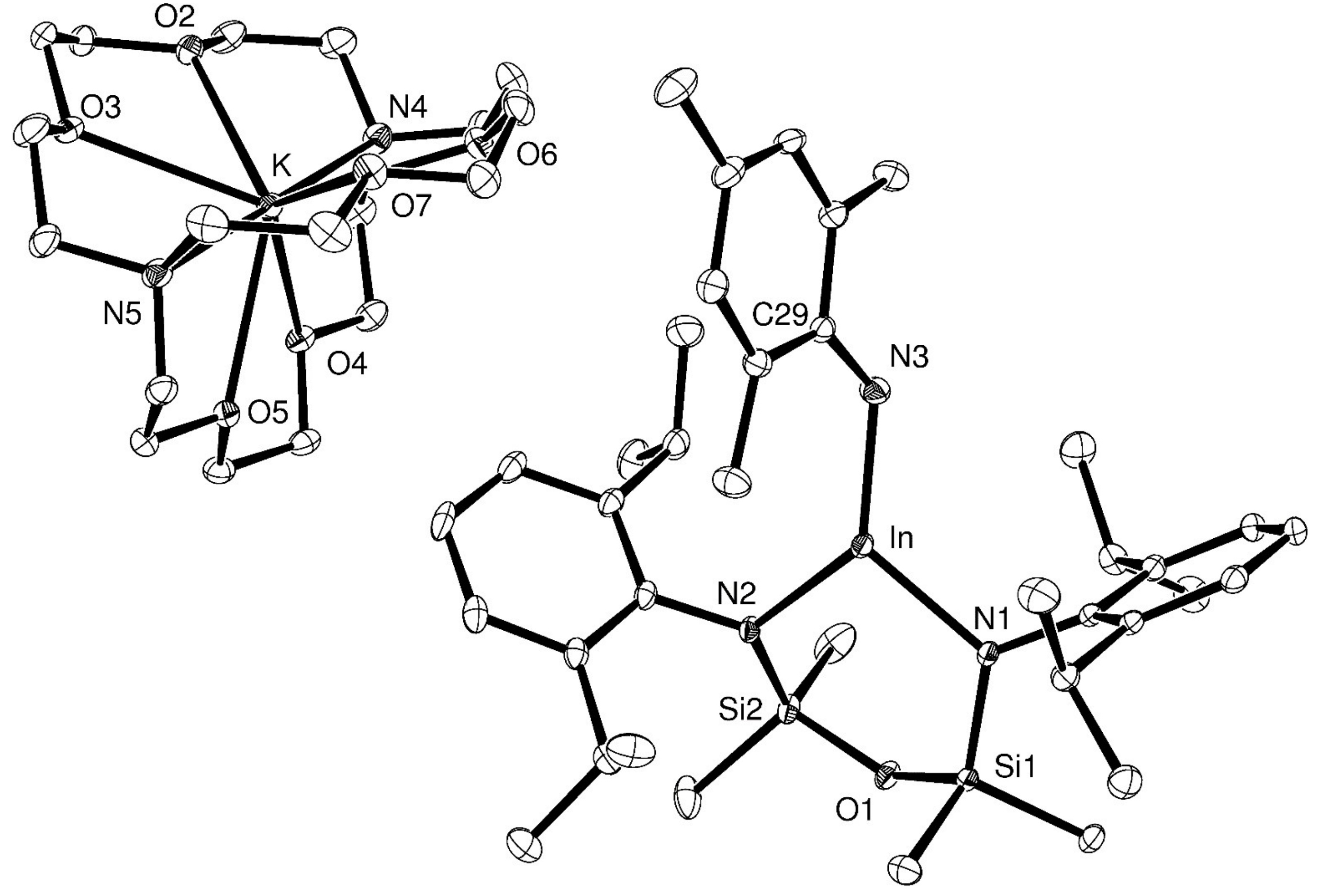

**Figure S21:** View of In(NMes) components of (a) [**5**]$_2$ and (b) **6** projected perpendicular to the In-N-C$_{ipso}$ plane

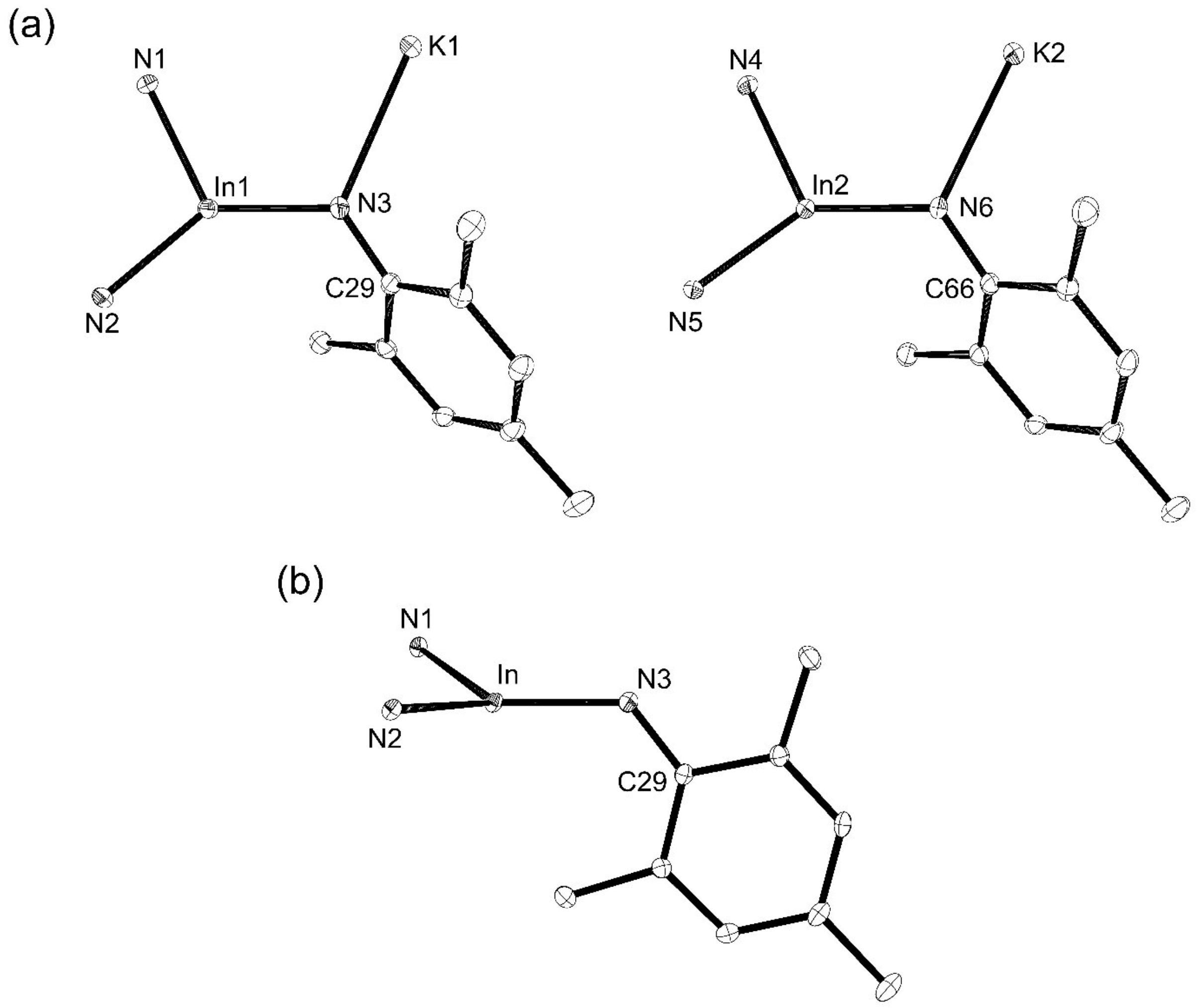

*Representative procedure for the preparation of K[In(NON$^{Ar}$)($N_4${Mes}$_2$-1,4] (**7**) and K[In(NON$^{Ar}$)($N_4${Mes}{Si$Me_3$}-1,4] (**8**)*

To a yellow toluene solution of compound **5** (0.050 g, 0.06 mmol) was added one equivalent of $RN_3$ (R = TMS or Mes, 0.06 mmol) in toluene at room temperature. The solution was stirred for 5 minutes after which time the solution had become colorless. The solution was concentrated *in vacuo* and stored at –30˚C overnight to afford a colourless crystalline solid. Yield: compound **7**, 0.052 g, 93 %; compound **8**, 0.048 g, 85 %.

Compound **7**:

$^1$H NMR ($CD_3CN$, 600 MHz): $\delta$ 6.92 (d, *J* = 7.5, 4H, $C_6H_3$), 6.83 (t, *J* = 7.5, 2H, $C_6H_3$), 6.59 (s, 4H, $C_6H_2$), 3.79 (sept, *J* = 6.7, 4H, C*H*$Me_2$), 2.14 (s, 6H, Mes-4-*Me*$C_6H_2$), 1.75 (s (br), 12H, Mes-2,6-$Me_2C_6$H), 1.06 (d, *J* = 6.7, 12H, CH$Me_2$), 0.74 (d, *J* = 6.7, 12H, CH$Me_2$), -0.08 (s, 12H, Si$Me_2$).

$^{13}$C{$^1$H} NMR ($CD_3CN$, 151 MHz): $\delta$ 149.6, 148.3, 147.6, 134.6, 130.7, 130.4, 124.1, 122.5(Ar-$C_6H_3$ and Mes-$C_6H_2$), 32.3, 27.6, 26.8, 26.1 (Mes-2,4,6-$Me_3C_6H_2$), 23.4 (*C*H$Me_2$), 21.0, 20.8 (CH$Me_2$), 3.5 (Si$Me_2$).

Compound **8**:

$^1$H NMR ($CD_3CN$, 600 MHz): $\delta$ 6.93 (dd, *J* = 7.5, 1.9, 2H, $C_6H_3$), 6.88 (dd, *J* = 7.6, 1.8, 2H, $C_6H_3$), 6.81 (t, *J* = 7.5, 2H, $C_6H_3$), 6.33 (s, 2H, $C_6H_2$), 3.94 (sept, *J* = 6.8, 1H, CH$Me_2$), 2.00 (s, 3H, Mes-4-*Me*$C_6H_2$), 1.16 (d, *J* = 6.8, 6H, CH$Me_2$), 1.13 (d, *J* = 6.8, 6H, CH$Me_2$), 0.90 (d, *J* = 6.8, 6H, CH$Me_2$), (s, 6H, Mes-2,6-$Me_2C_6H_2$), 0.45 (d, *J* = 6.8, 6H, CH$Me_2$), 0.43 (s, 9H, Si$Me_3$), 0.04 (s, 6H, Si$Me_2$), 0.03 (s, 6H, Si$Me_2$).

$^{13}$C NMR ($CD_3CN$, 151 MHz): $\delta$ 149.4, 148.1, 147.4, 137.5, 132.6, 128.6, 124.3, 123.9, 122.3 (Ar-$C_6H_3$ and Mes-$C_6H_2$), 32.3, 27.6, 27.5 (Mes-2,4,6-$Me_3C_6H_2$), 27.4 (*C*H$Me_2$), 26.8, 25.1, 20.7, 18.3 (CH$Me_2$), 3.7 (Si$Me_3$), 3.4, 2.8 (Si$Me_2$).

Extreme sensitivity to moisture and/or oxygen, combined with variable amounts of incorporated solvent as a result of sample preparation precluded the acquisition of accurate elemental analysis results for **7** and **8**.

**Figure S22:** $^1$H NMR spectrum ($CD_3CN$, 600 MHz, 298 K) of K[In(NON$^{Ar}$)($N_4${Mes}$_2$-1,4] (**7**)

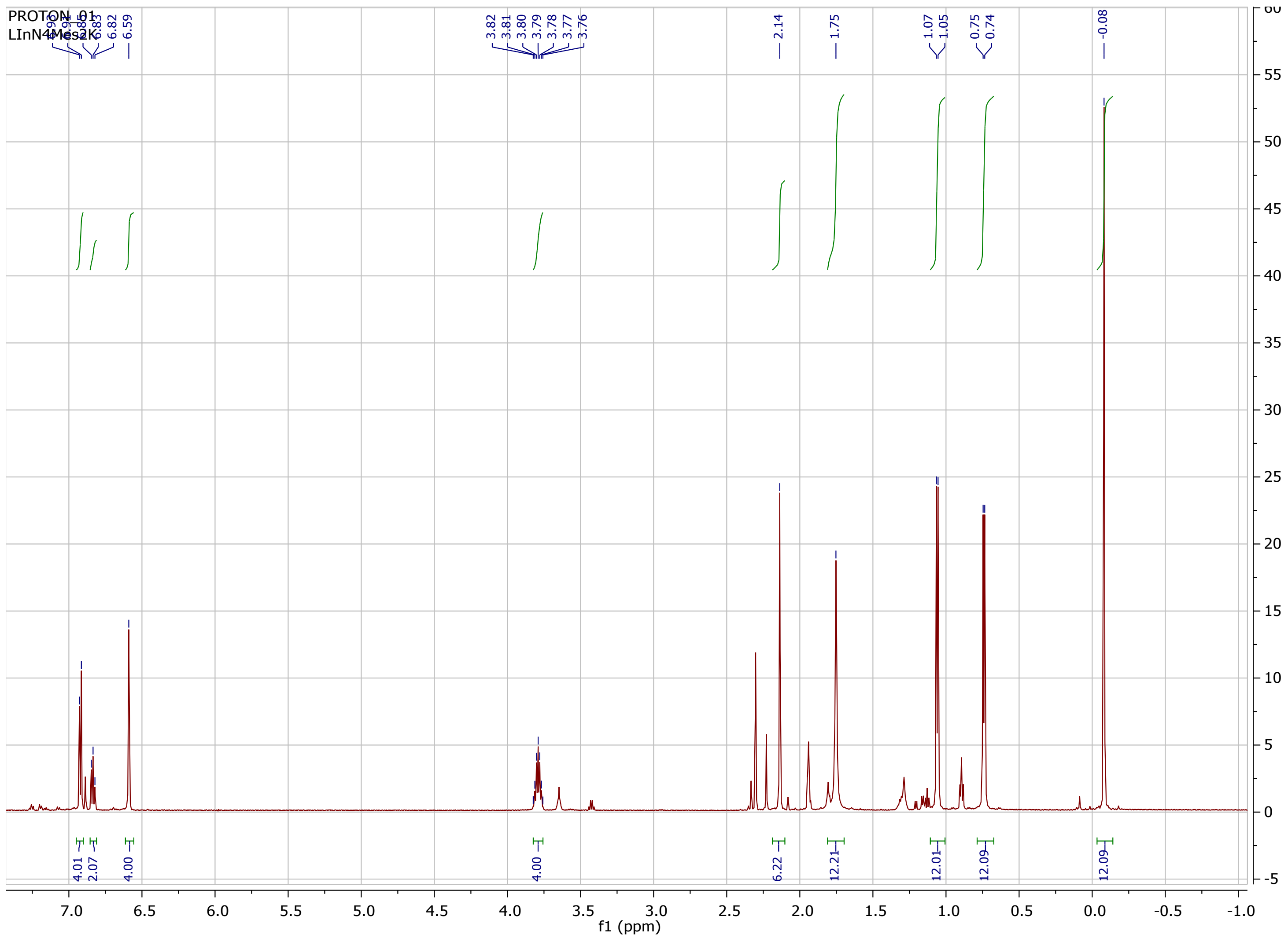

**Figure S23:** $^{13}C\{^{1}H\}$ NMR spectrum ($CD_3CN$, 151 MHz, 298 K) of K[In(NON$^{Ar}$)($N_4$\{Mes\}$_2$-1,4] (**7**)

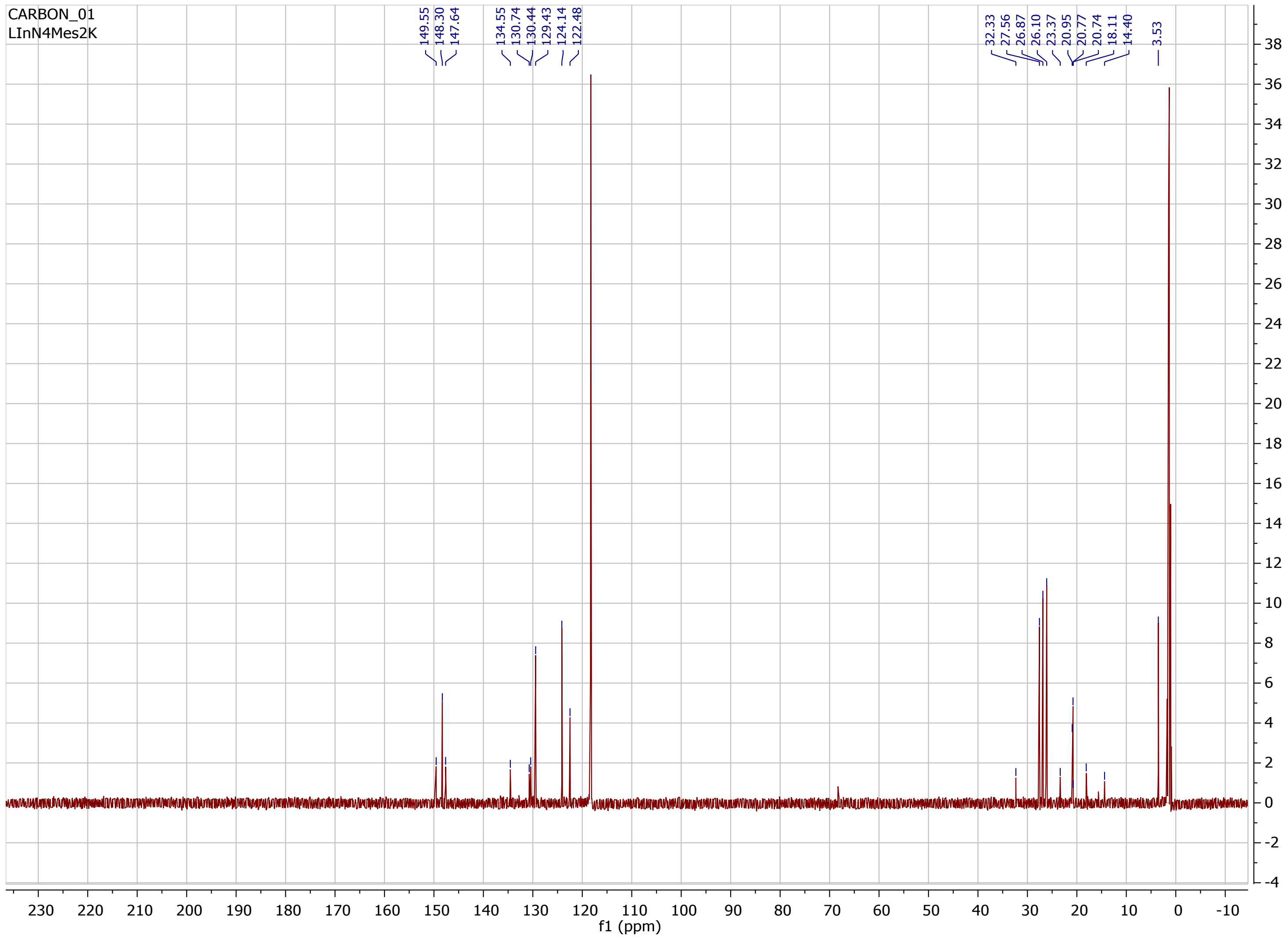

**Figure S24a:** ORTEP of the asymmetric unit of [K(In{NON$^{Ar}$}{$N_4$(Mes)$_2$-1,4})]$_2$ ([**7·(toluene)**]$_2$) (ellipsoids 30%, hydrogen atoms and toluene solvate omitted).

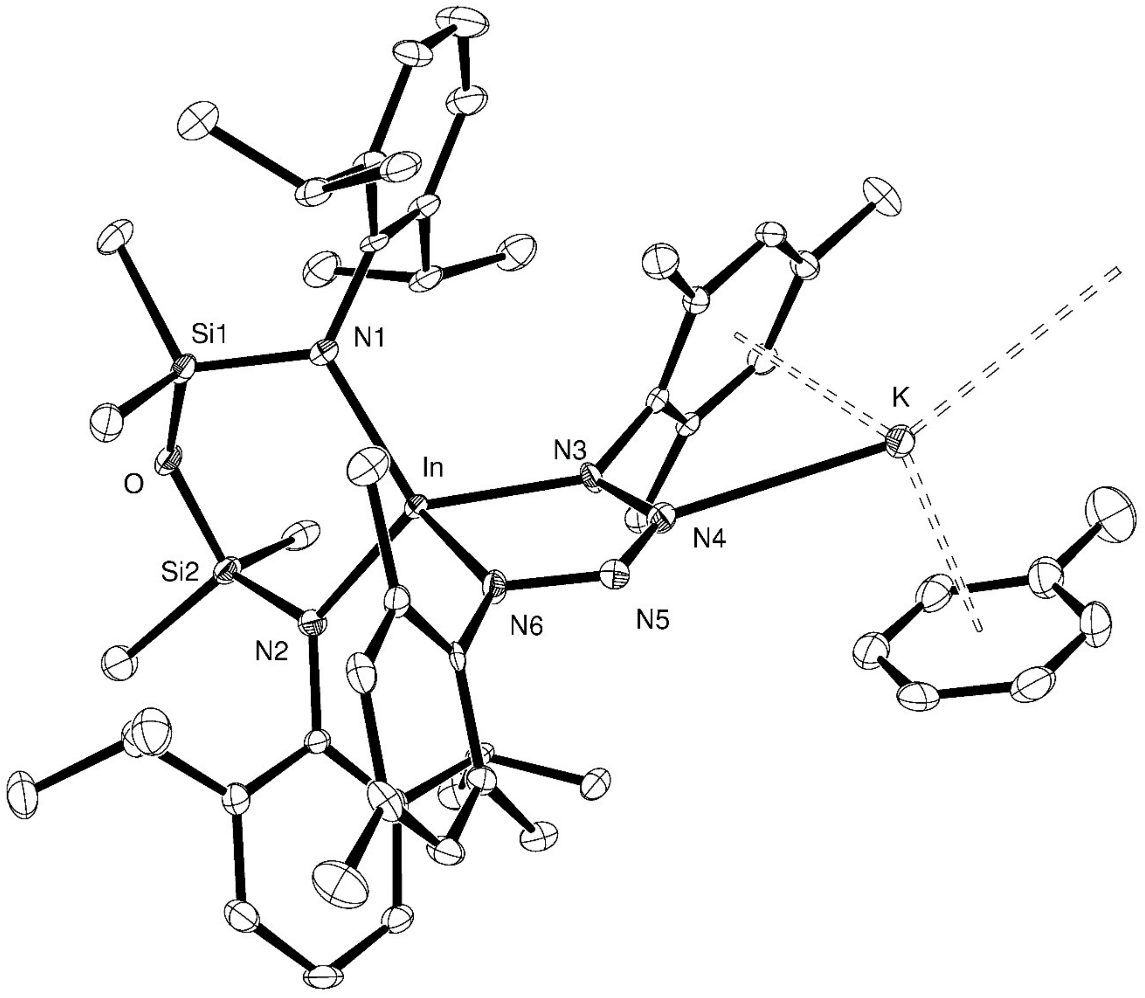

**Figure S24b:** ORTEP of the core of [K(In{NON$^{Ar}$}{$N_4$(Mes)$_2$-1,4})]$_2$ ([**7·(toluene)**]$_2$) (ellipsoids 30%, hydrogen atoms, selected carbon atoms and toluene solvate omitted).

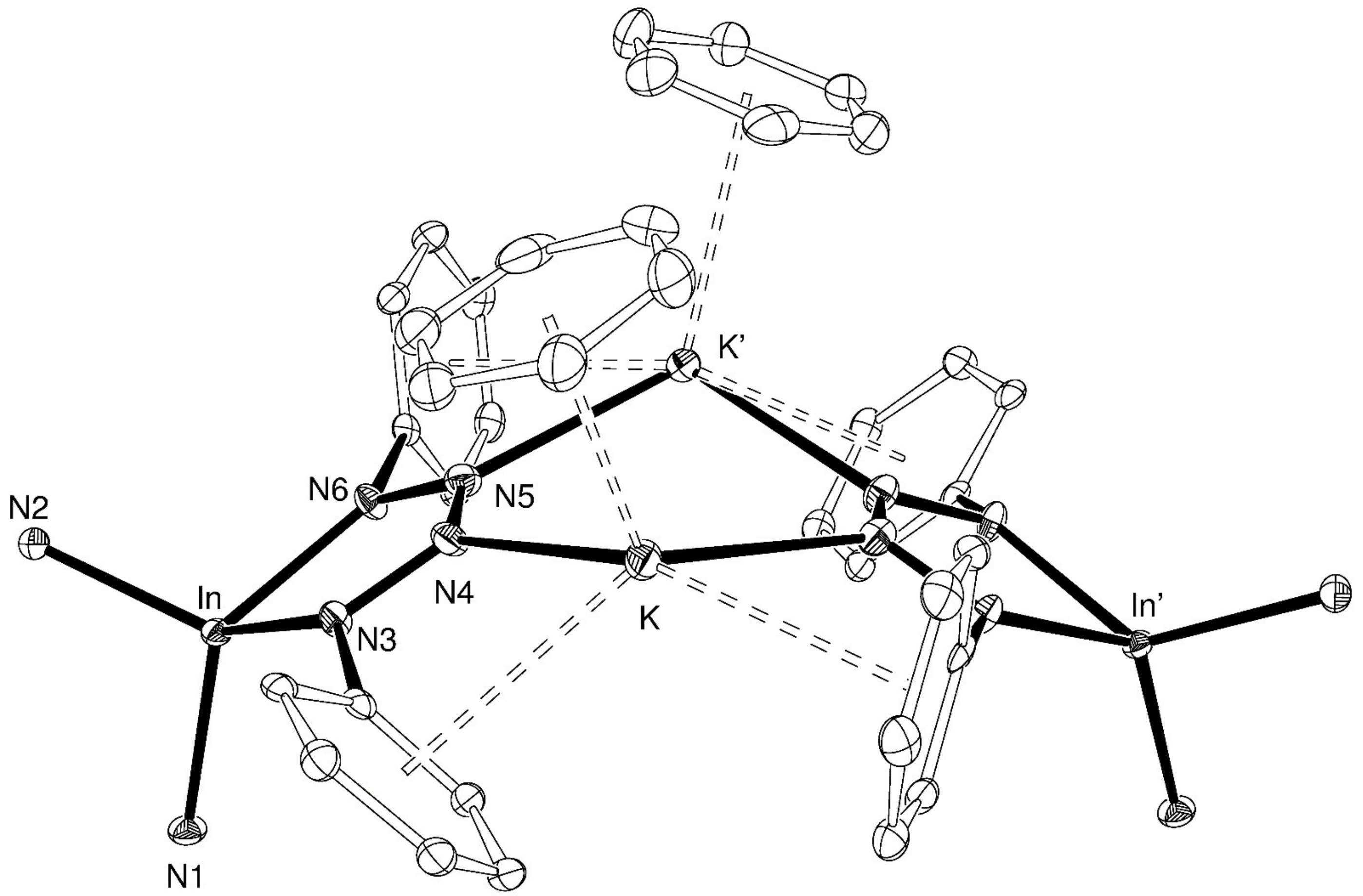

**Figure S25:** $^{1}H$ NMR spectrum ($CD_3CN$, 600 MHz, 298 K) of K[In(NON$^{Ar}$)($N_4${Mes}{$SiMe_3$}-1,4] (**8**)

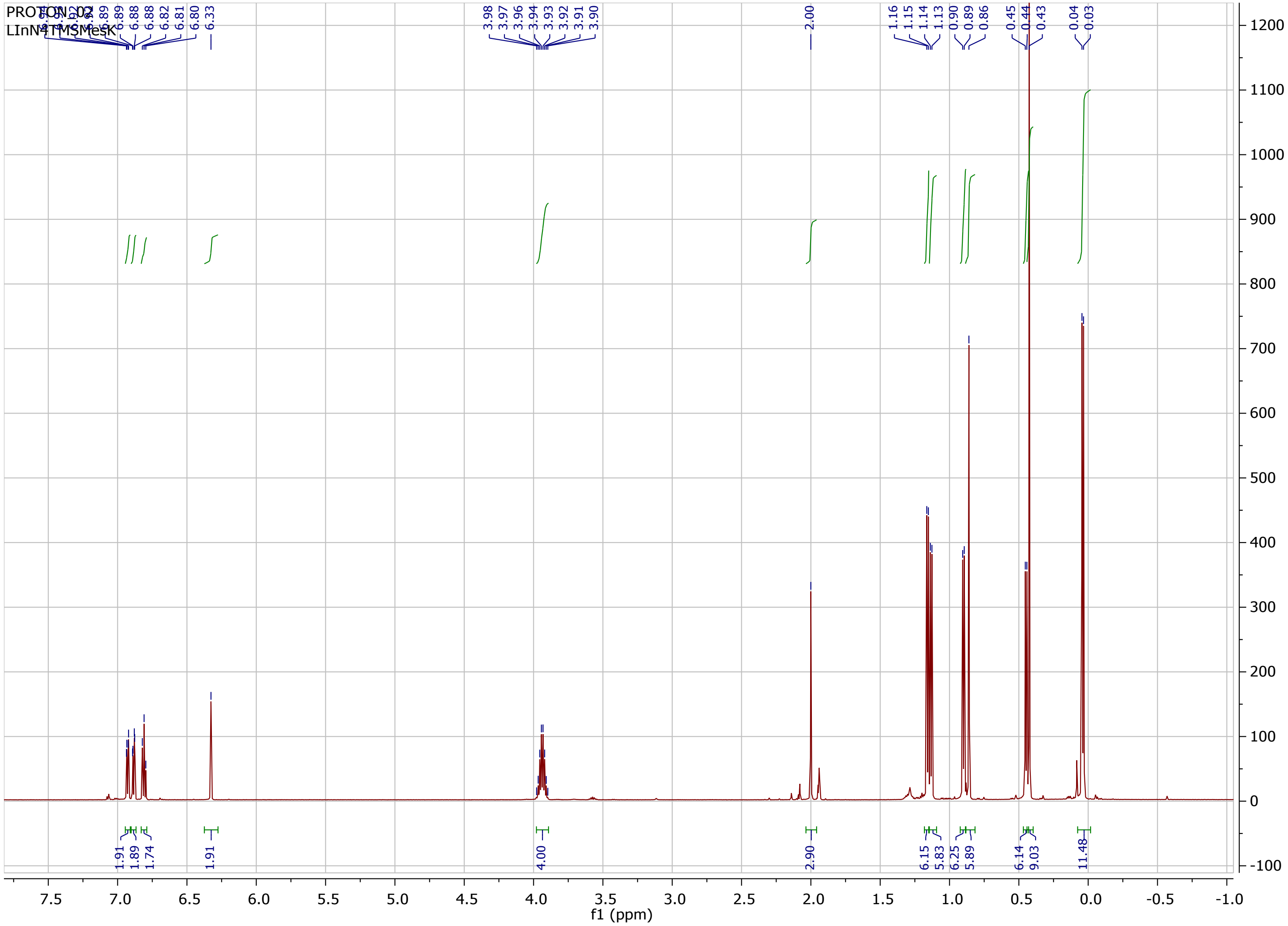

**Figure S26:** $^{13}C\{^{1}H\}$ NMR spectrum ($CD_3CN$, 151 MHz, 298 K) of K[In(NON$^{Ar}$)($N_4$\{Mes\}\{SiMe$_3$\}-1,4] (**8**)

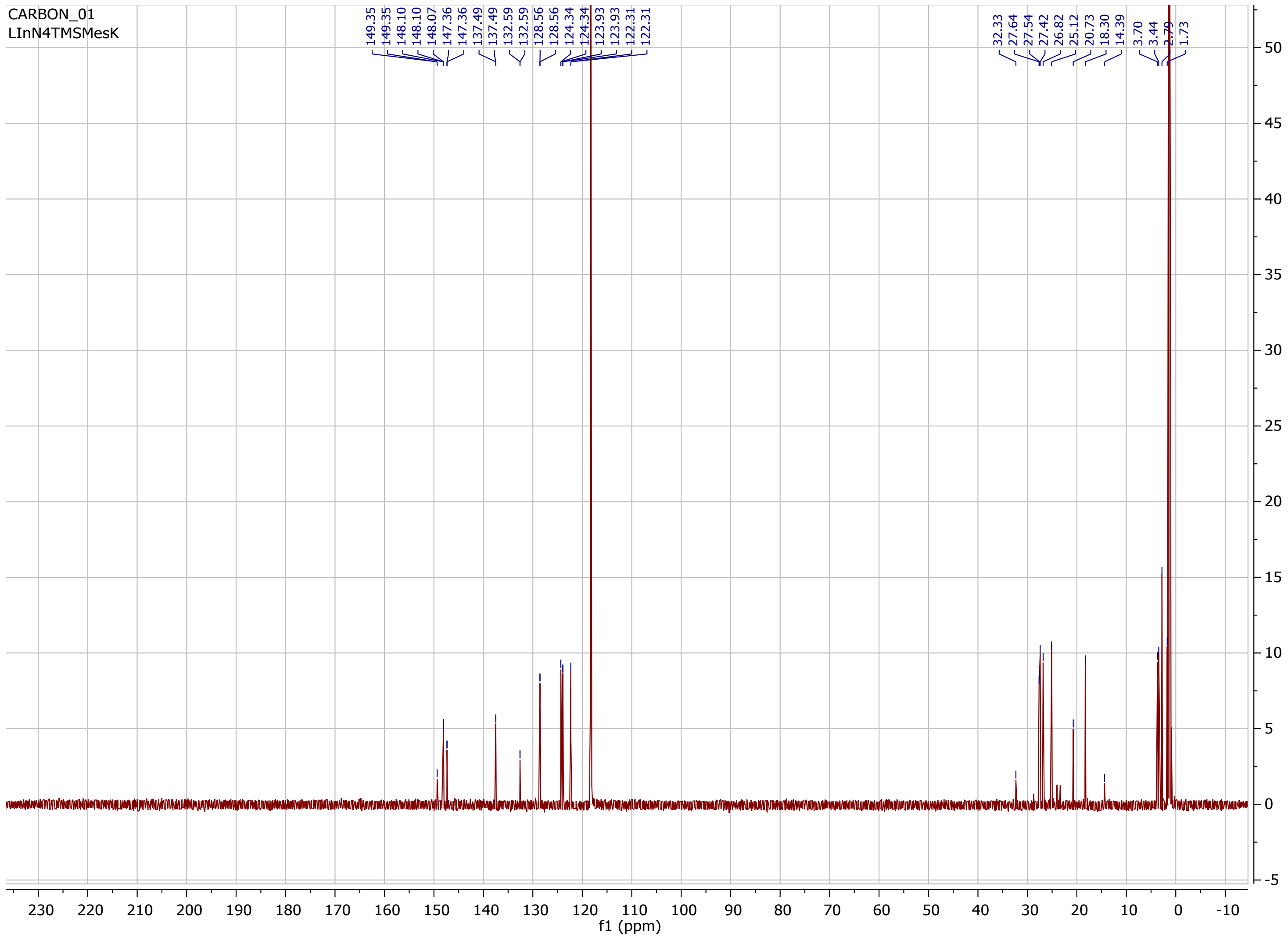

**Figure S27a:** ORTEP of [K(In{NON$^{Ar}$}{$N_4$(Mes)($SiMe_3$)-1,4})]$_2$ ([**8**]$_2$) (ellipsoids 20%, hydrogen atoms omitted).

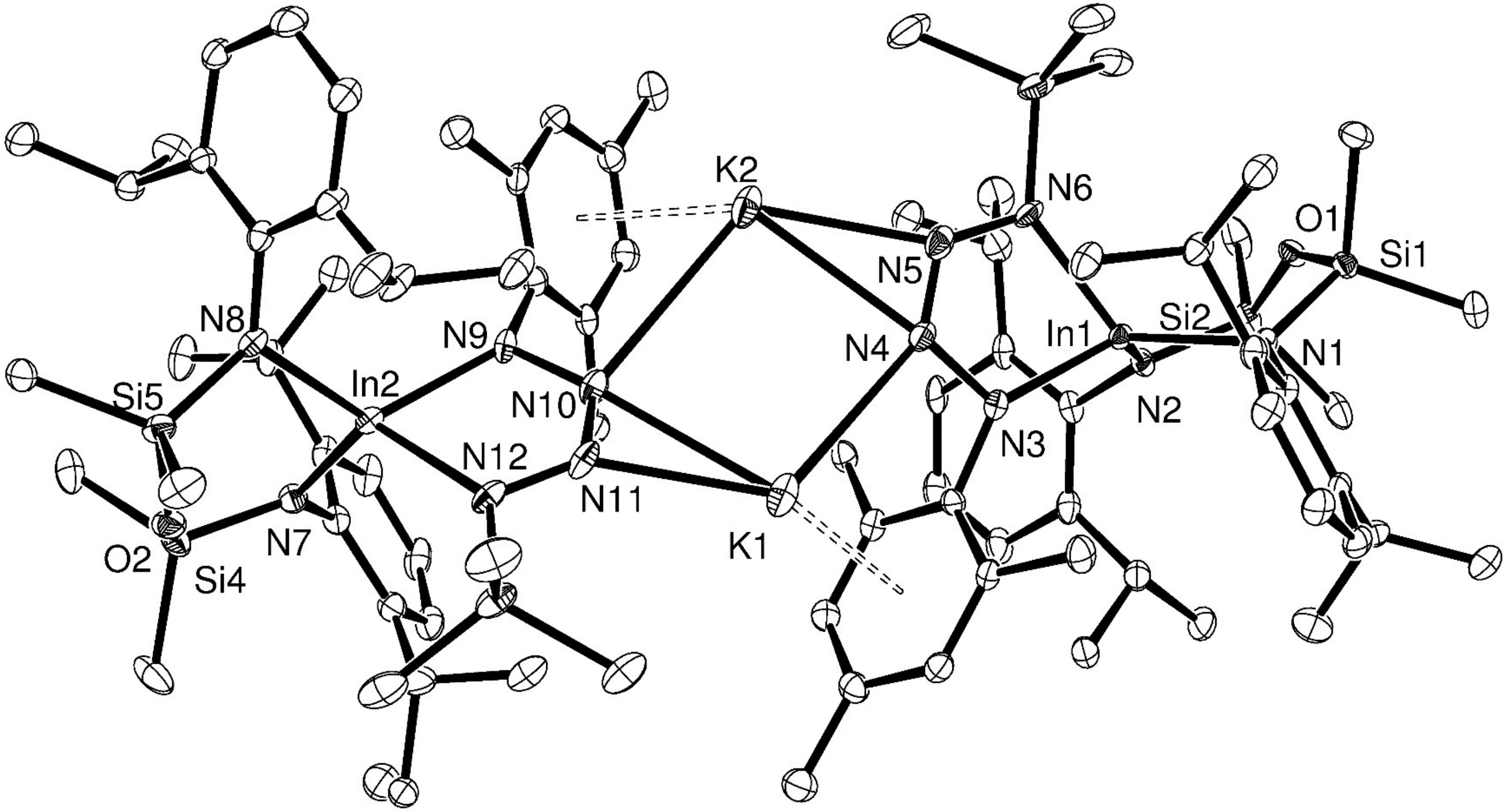

**Figure S27b:** ORTEP of the core of [K(In{NON$^{Ar}$}{$N_4$(Mes)($SiMe_3$)-1,4})]$_2$ ([**8**]$_2$) (ellipsoids 20%, hydrogen atoms and selected carbon atoms omitted).

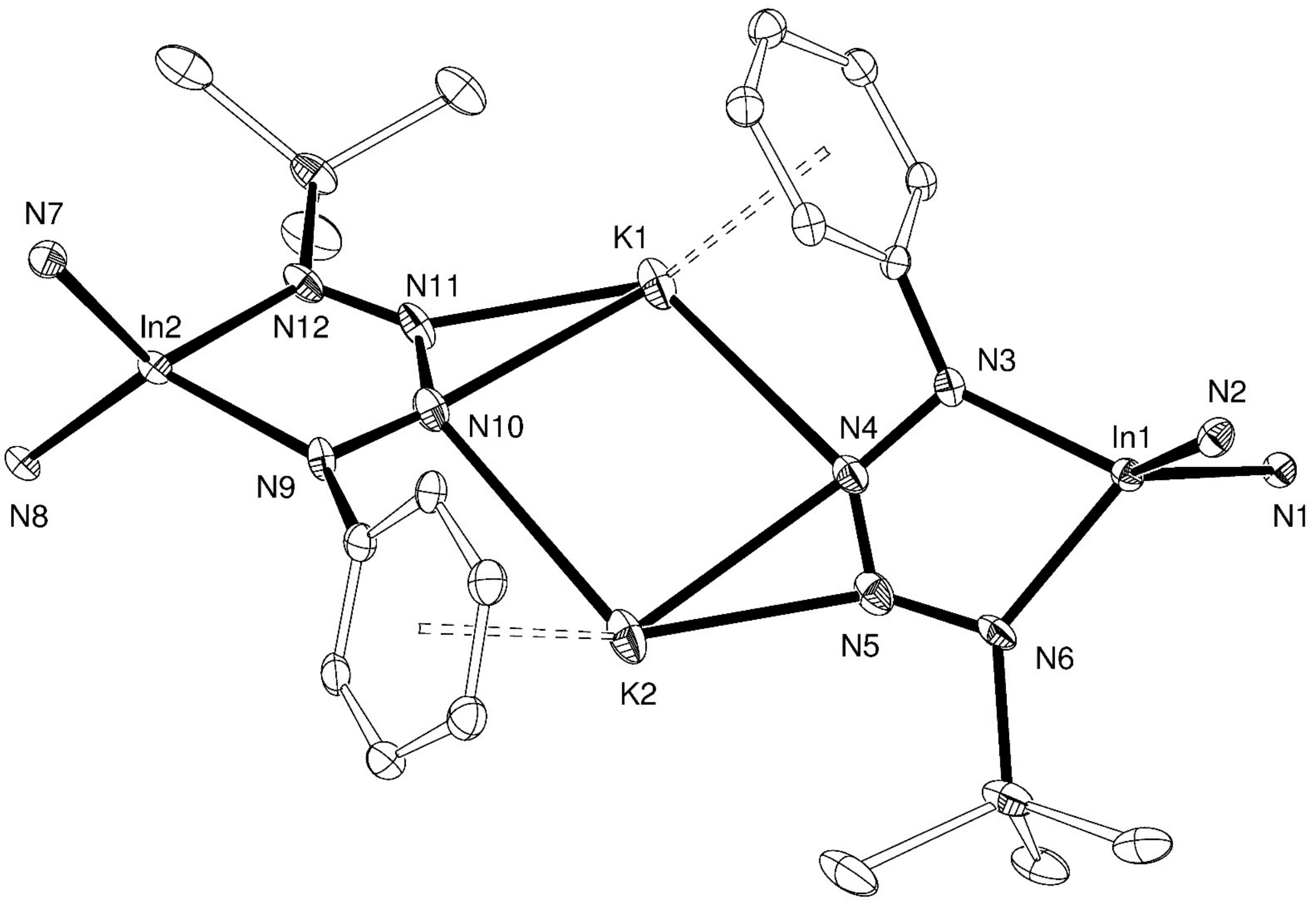

**Figure S28:** ORTEP of [K(18-crown-6)][In(NON$^{Ar}$)(N$_4${Mes}$_2$-1,4)] (**7·(18-c-6)**) (ellipsoids 30%, hydrogen atoms and toluene (x2) solvate omitted)

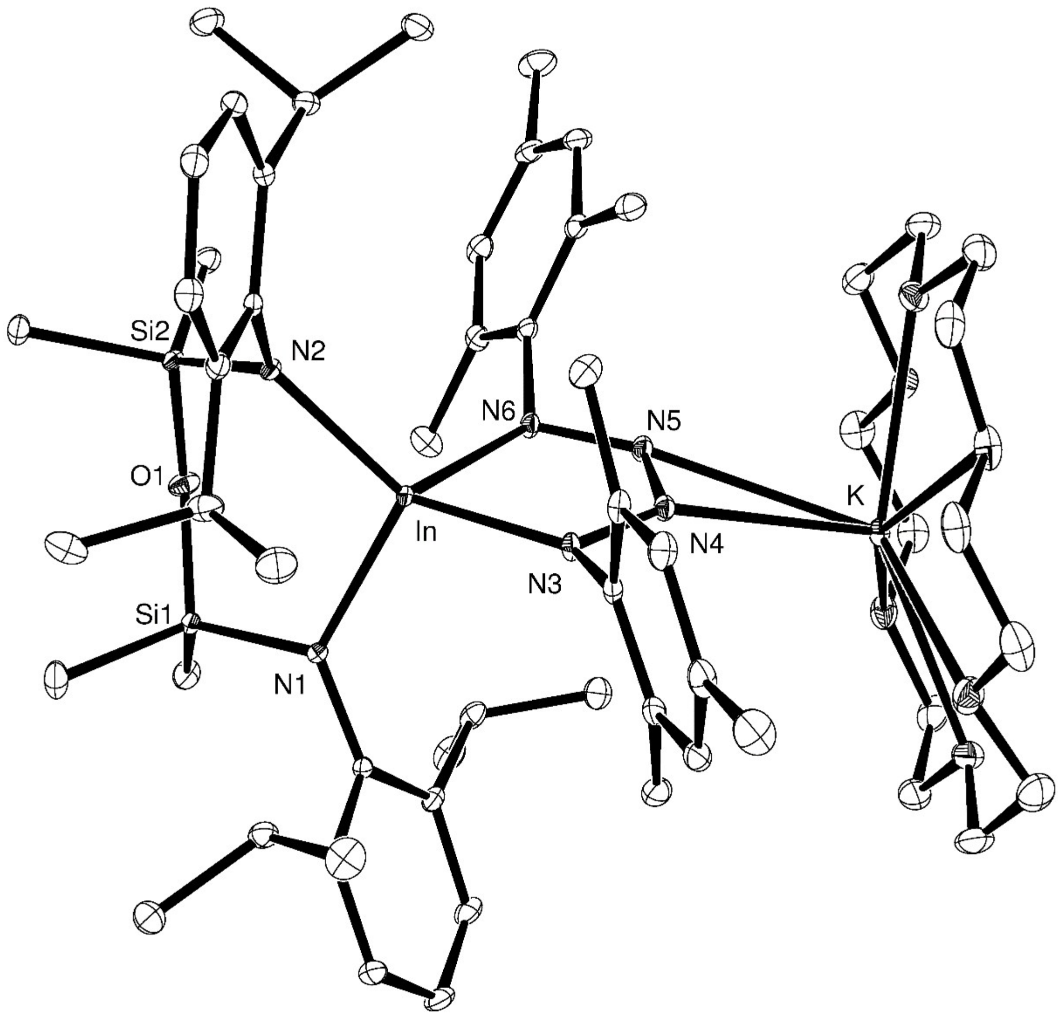

**Crystallography**

Crystals were covered in inert oil and suitable single crystals were selected under a microscope and mounted on an Agilent SuperNova diffractometer fitted with an EOS S2 detector. Data were collected at the temperature indicated using focused microsource Mo K$\alpha$ radiation at 0.71073 Å ([**1_{THF}$_3$**]$_2$, **1_{Et$_2$O}$_2$**, [**7·(toluene)**]$_2$) or Cu K$\alpha$ radiation at 1.54184 Å (**1_{18-c-6}**, **2**, **2_benzene,** [**3**]$_2$, **4**, [**5**]$_2$, **6**, [**8**]$_2$, **7·(18-c-6)**). Intensities were corrected for Lorentz and polarisation effects and for absorption using multi-scan methods.[S2] Space groups were determined from systematic absences and checked for higher symmetry. All structures were solved using direct methods with SHELXS,[S3] refined on $F^2$ using all data by full matrix least-squares procedures with SHELXL-97,[S4] WinGX.[S5] Non-hydrogen atoms were refined with anisotropic displacement parameters. Hydrogen atoms were placed in calculated positions or manually assigned from residual electron density where appropriate unless otherwise stated. The functions minimized were Σ*w*(*F2*o-*F2c*), with *w* = [σ2(F2o) + *a*P2 + *bP*]-1, where *P* = [max(Fo)2 + 2F2c]/3. The isotropic displacement parameters are 1.2 or 1.5 times the isotropic equivalent of their carrier atoms.

Additional information:

*[$K_2$({NON$^{Ar}$}{THF}$_3$)]$_2$ ([**1_{THF}$_3$**]$_2$)*: The molecule is polymeric, running parallel to the *c*-axis. A methylene groups of one of the THFs is disordered and was modelled over two sites with SIMU and DELU restraints used to obtain more satisfactory displacement ellipsoids. Alternative solutions considering missed (pseudo) symmetry failed to give a satisfactory model.

*$K_2$[(NON$^{Ar}$)($Et_2O$)$_2$] (**1_{Et$_2$O}$_2$**)*: The molecule is polymeric, running parallel to the *b*-axis.

*$K_2$[(NON$^{Ar}$)(18-crown-6)] (**1_{18-c-6}**)*: The molecule is dimeric, with the two halves related by an inversion center.

*K[In(NON$^{Ar}$)$Cl_2$] (**2**)*: The molecule is polymeric.

*K[In(NON$^{Ar}$)$Cl_2$]·benzene (**2_benzene**)*: The asymmetric unit contains 1/2 a molecule of benzene located on a two-fold rotation axis.

*K(In{NON$^{Ar}$})]$_2$ ([**3**]$_2$)*: The asymmetric unit contains 1/2 a molecule of hexane located on an inversion center. A methyl group of one of the *i*Pr groups and a methyl group of one of the

$SiMe_2$ groups are disordered and were modelled over two positions with SADI / SIMU / DELU restraints.

*K[In(NON$^{Ar}$)(N(H){2-($CPh_2$)-6-($CHPh_2$)-4-tBu$C_6H_2$}]* (**4**): The asymmetric unit contains a molecule of toluene solvate. The hydrogen atom on the amido-nitrogen atom was located on the difference map and freely refined.

*[K(In{NON$^{Ar}$}{NMes})]$_2$* (*[**5**]$_2$*): The asymmetric unit contains four molecules of toluene. One of these solvate molecules is disordered and was modelled over two positions with the carbon atoms isotropic; the $C_6$-ring of the lower occupancy orientation was constrained in a regular hexagon using the AFIX66 command. The carbon atoms of separate toluene were modelled using SIMU and DELU restraints to obtain more satisfactory displacement ellipsoids.

*[K(In{NON$^{Ar}$}{$N_4$(Mes)$_2$-1,4})]$_2$* (*[**7·(toluene)**]$_2$*): The molecule is dimeric, with the two halves related by a two-fold rotation axis passing through the center of the $K_2N_4$ ring. The toluene solvate is disordered and was modelled over two positions with rigid constraints (AFIX66) on the $C_6$-rings of both components.

*[K(In{NON$^{Ar}$}{$N_4$(Mes)($SiMe_3$)-1,4})]$_2$* (*[**8**]$_2$*): A methyl group on one of the *i*Pr substituents is disordered and was modelled over two positions. It was not possible to obtain satisfactory ellipsoids and both components were refined as isotropic models.

The asymmetric unit contains two poorly defined toluene solvate molecules that have been treated as a diffuse contribution to the overall scattering without specific atom positions by SQUEEZE/PLATON. Details are given in the .cif file.

*[K(18-crown-6)][In(NON$^{Ar}$)($N_4${Mes}$_2$-1,4)]* (***7·(18-c-6)***): The asymmetric unit contains two molecules of toluene. One of these is disordered and was modelled over 2 positions with all carbon atom isotropic.

**Computational Methods**

All structural optimisations were carried out with the Gaussian 09 suite of programs (Revision D.01),[S6] using the density functional method (DFT) with the PBE0-D3BJ hybrid functional[S7] (including empirical corrections for dispersion interactions) and the balanced, polarised def2-SVP basis-set[S8] of double-ζ quality. Frequency calculations at the same level of theory were employed to ensure that the obtained structures are minima on the potential energy surface.

All subsequent calculations for the analysis of the electronic structure were performed at the obtained geometry with the same method (PBE0-D3BJ) but employed the large def2-TZVP basis set of triple-ζ quality.

The bonding was analysed using the Natural Bond Orbital (NBO) approach[S9] using the NBO 3.0 program,[S10] and Wiberg Bond Indices (WBI) were computed.[S11] Additional analysis was carried out with the Quantum Theory of Atoms in Molecules (QTAIM) approach using the AIMAll programme package.

Molecular and MO/NBO graphics and were rendered with GaussView 5.0.9.[S12]

**Table S1** Calculated structural parameters and charges (q) from the Natural Population Analysis (NPA), Wiberg Bond Indices (WBI) as well as electron densities (ρ), values of the Laplapcian of the electron density ($\nabla^2\rho$) and ellipticity (ε) at the $InN^{Mes}$-bond critical point for compounds **5** and the anionic $[In(NON^{Ar})(NMes)]^{-}$ component of compound **6** ([**6**]$^{-}$). Note that corresponding values for the $In\text{-}N^{NON\text{-}Ar}$ bond are given in parentheses where appropriate.

| | $K[In(NON^{Ar})(NMes)]$ (**5**) | $[In(NON^{Ar})(NMes)]^{-}$ ([**6**]$^{-}$) |
|---|---|---|
| r ($In,N^{Mes}$) | 2.00 Å | 1.97 Å |
| a ($In,N^{Mes},C$) | 123° | 126° |
| r ($N^{Mes},K$) | 2.55 Å | n/a |
| q (In) | 1.94 | 1.88 |
| q ($N^{Mes}$) | −1.36 | −1.20 |
| WBI ($InN^{Mes}$) | 0.59<br>(0.22/0.29) | 0.71<br>(0.25/0.25) |
| ρ | 0.120<br>(0.090/0.101) | 0.125<br>(0.092/0.091) |
| $\nabla^2\rho$ | +0.373<br>(+0.295/+0.315) | +0.400<br>(+0.301/+0.284) |
| ε | 0.079<br>(0.104/0.098) | 0.072<br>(0.104/0.090) |

**Figure S29** QTAIM derived molecular graphs of K[In(NON$^{Ar}$)(NMes)] (**5**). Bond paths are indicated in solid black lines (dotted black lines for weak interactions), the corresponding bond critical points are displayed in green. Ring and cage critical points are not displayed.

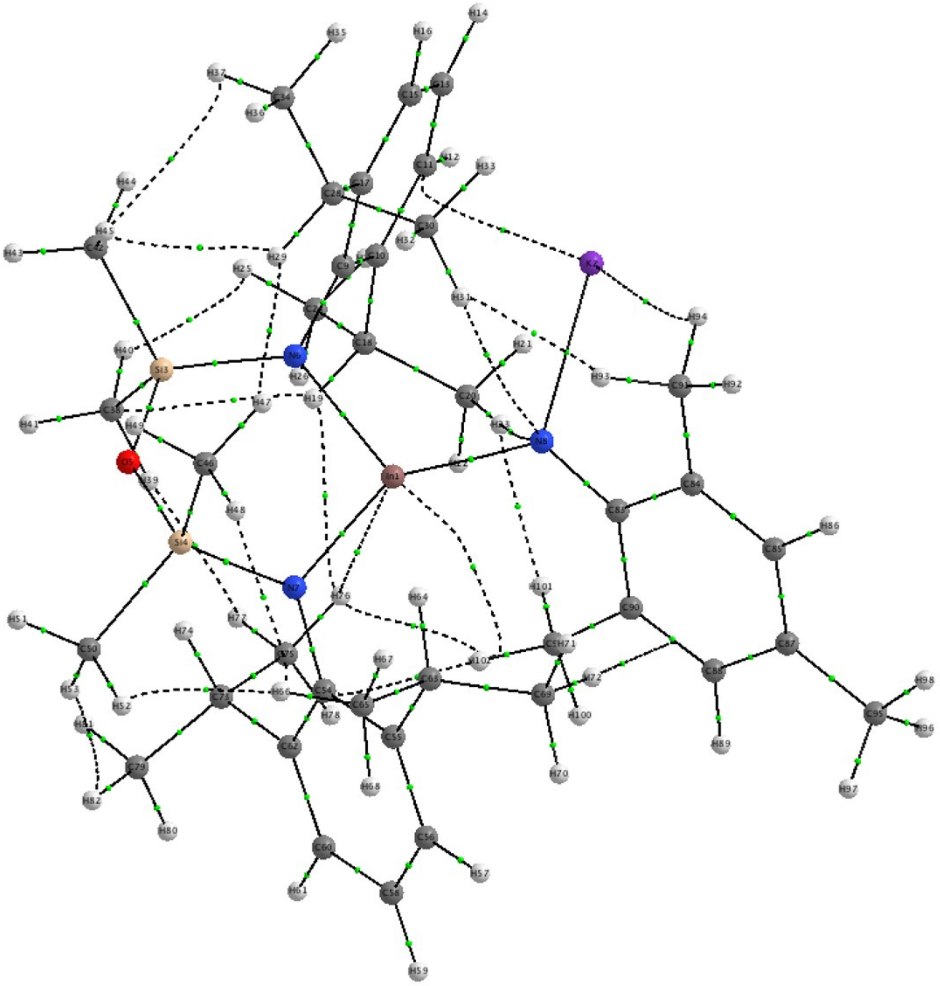

**Figure S30** QTAIM derived molecular graphs of [In(NON$^{Ar}$)(NMes)]$^{-}$ ([**6**]$^{-}$). Bond paths are indicated in solid black lines (dotted black lines for weak interactions), the corresponding bond critical points are displayed in green. Ring and cage critical points are not displayed.

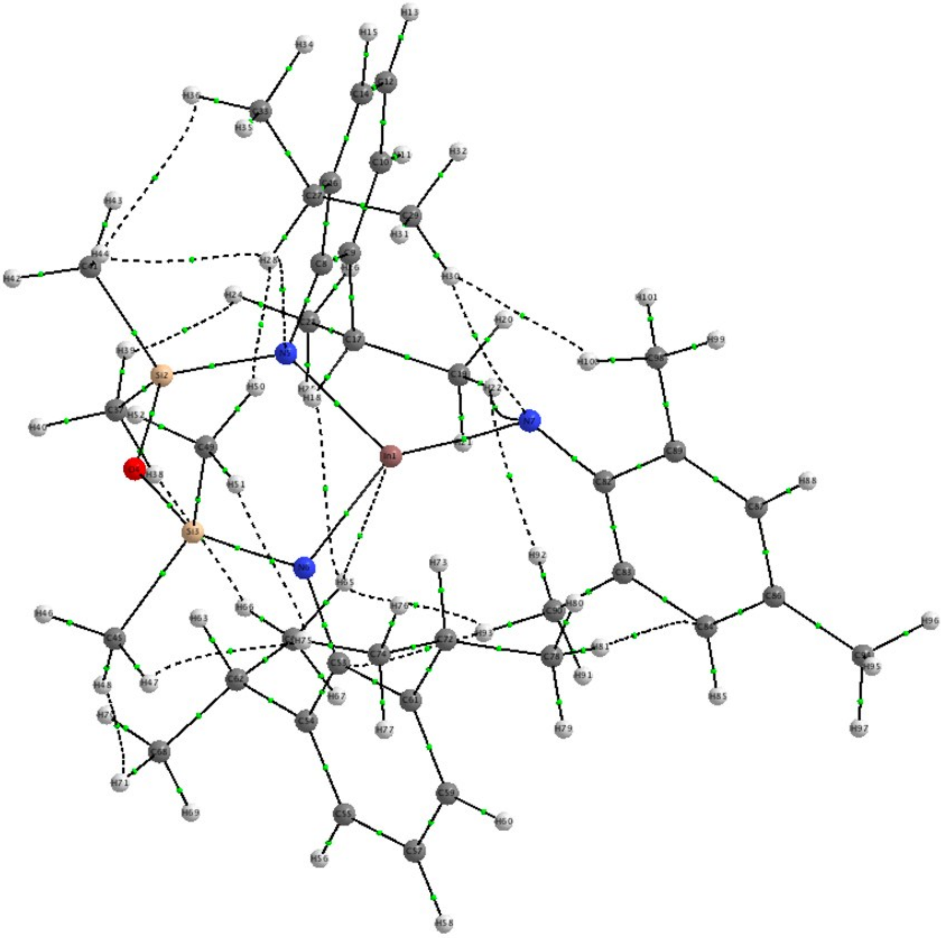

**Figure S31** Contour plot of the Laplacian of the electron density of [In(NON$^{Ar}$)(NMes)]$^-$ ([**6**]$^-$) in the N-N-In-N plane. Selected bond paths are indicated as solid black lines and their corresponding bond critical points are displayed in green.

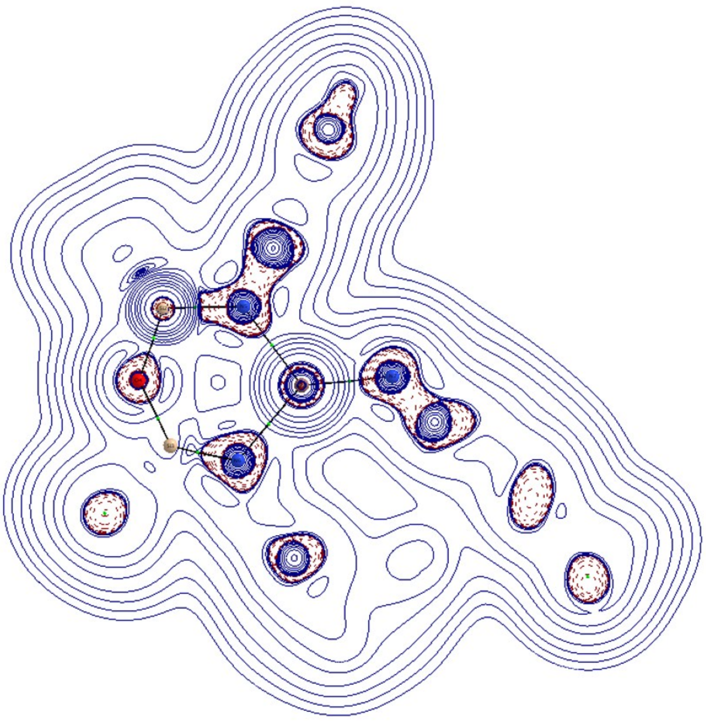

Enlarged area of the above plot

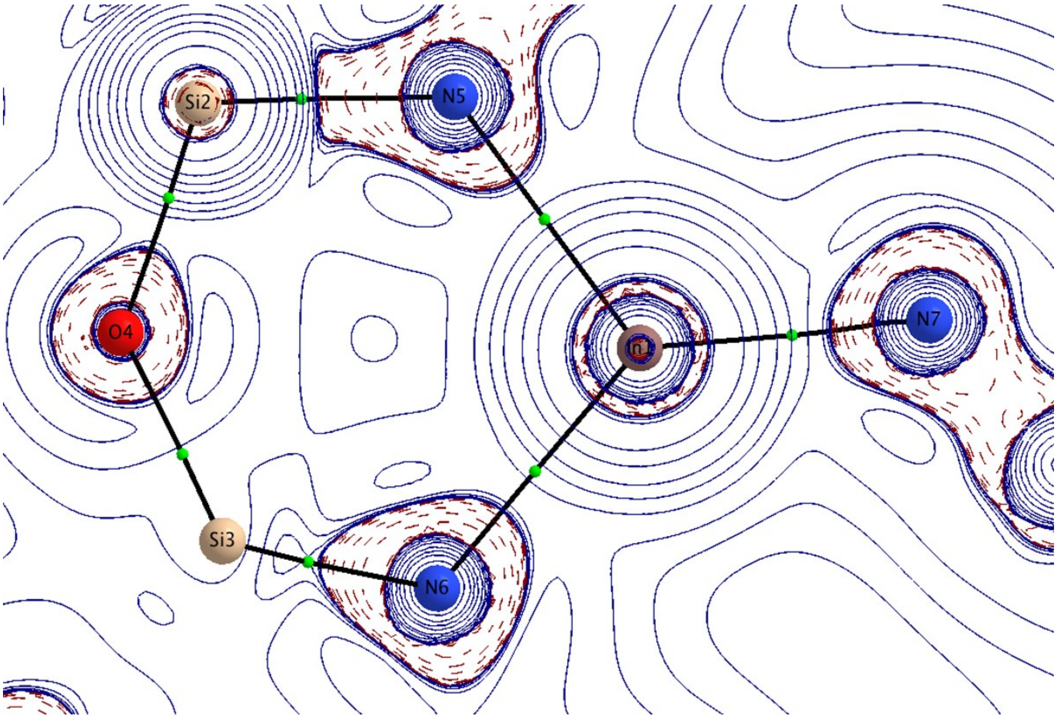

**Table S2** NBO Plots for $[In(NON^{Ar})(NMes)]^{-}$ ($[\mathbf{6}]^{-}$) showing the orbitals that contribute to the In-N interaction in different resonance structures. The non-Lewis (n-L) component is given in parentheses for each resonance structure.

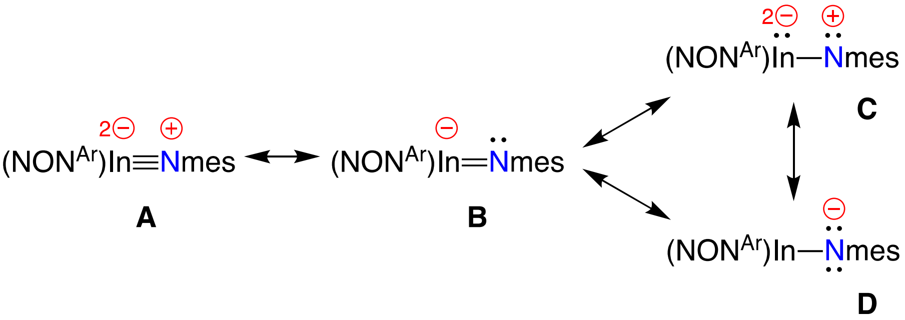


| **Resonance structure A**<br>(n-L = 1.965 %) | **Resonance structure B**<br>(n-L = 1.990 %) | **Resonance structure D**<br>(n-L = 2.094 %) |
|---|---|---|
| 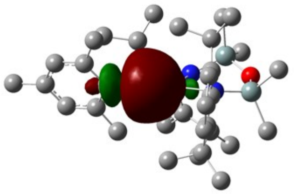<br>σ | 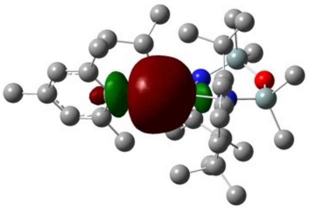<br>σ | 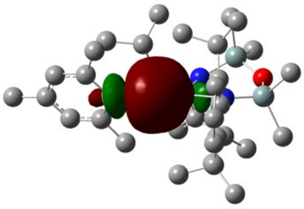<br>σ |
| 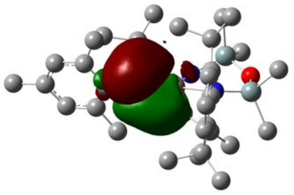<br>π | 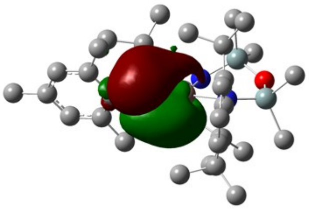<br>π | 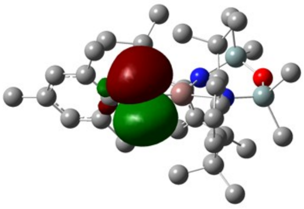<br>lp |
| 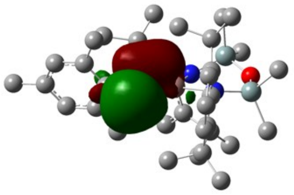<br>π | 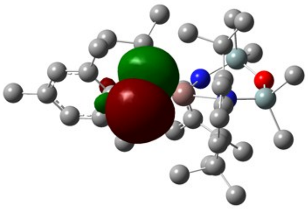<br>l p | 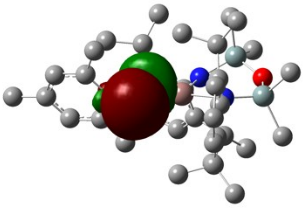<br>lp |

**References**


[S1] D. B. Leznoff, G. Mund, K. C. Jantunen, P. H. Bhatia, A. J. Gabert, R. J. Batchelor, *J. Nucl. Sci. Tech.* **2002**, 406-409.

[S2] R. Blessing, *Acta Crystallogr.* **1995**, *A51*, 33-38.

[S3] G. Sheldrick, *Acta Crystallogr.* **2008**, *A64*, 112-122.

[S4] SHELXL-97 G. M. Sheldrick, University of Gottingen, Germany, (1997).

[S5] L. Farrugia, *J. Appl. Cryst.* **1999**, *32*, 837-838.

[S6] *Gaussian 09* M. J. Frisch, G. W. Trucks, H. B. Schlegel, G. E. Scuseria, M. A. Robb, J. R. Cheeseman, G. Scalmani, V. Barone, B. Mennucci, G. A. Petersson, H. Nakatsuji, M. Caricato, X. Li, H. P. Hratchian, A. F. Izmaylov, J. Bloino, G. Zheng, J. L. Sonnenberg, M. Hada, M. Ehara, K. Toyota, R. Fukuda, J. Hasegawa, M. Ishida, T. Nakajima, Y. Honda, O. Kitao, H. Nakai, T. Vreven, J. A. Montgomery Jr., J. E. Peralta, F. Ogliaro, M. J. Bearpark, J. Heyd, E. N. Brothers, K. N. Kudin, V. N. Staroverov, R. Kobayashi, J. Normand, K. Raghavachari, A. P. Rendell, J. C. Burant, S. S. Iyengar, J. Tomasi, M. Cossi, N. Rega, N. J. Millam, M. Klene, J. E. Knox, J. B. Cross, V. Bakken, C. Adamo, J. Jaramillo, R. Gomperts, R. E. Stratmann, O. Yazyev, A. J. Austin, R. Cammi, C. Pomelli, J. W. Ochterski, R. L. Martin, K. Morokuma, V. G. Zakrzewski, G. A. Voth, P. Salvador, J. J. Dannenberg, S. Dapprich, A. D. Daniels, Ö. Farkas, J. B. Foresman, J. V. Ortiz, J. Cioslowski, D. J. Fox, Wallingford, CT, USA, Gaussian, Inc., (2009).

[S7] C. Adamo, V. Barone, *J. Chem. Phys.* **1999**, *110*, 6158-6169.

[S8] F. Weigend, R. Ahlrichs, *Phys. Chem. Chem. Phys.* **2005**, *7*, 3297-3305.

[S9] a) J. P. Foster, F. Weinhold, *J. Am. Chem. Soc.* **1980**, *102*, 7211-7218; b) A. E. Reed, F. Weinhold, *J. Chem. Phys.* **1983**, *78*, 4066-4073; c) A. E. Reed, R. B. Weinstock, F. Weinhold, *J. Chem. Phys.* **1985**, *83*, 735-746; d) A. E. Reed, F. Weinhold, *J. Chem. Phys.* **1985**, *83*, 1736-1740; e) J. E. Carpenter, F. Weinhold, *J. Mol. Struct. (Theochem)* **1988**, *169*, 41-62; f) A. E. Reed, L. A. Curtiss, F. Weinhold, *Chem. Rev.* **1988**, *88*, 899-926; g) F. Weinhold, J. E. Carpenter, in *The Structure of Small Molecules and Ions* (Eds.: R. Naaman, Z. Vager), Plenum, **1988**, pp. 227-236.

[S10] a) *NBO 6.0* E. D. Glendening, J. K. Badenhoop, A. E. Reed, J. E. Carpenter, J. A. Bohmann, C. M. Morales, C. R. Landis, F. Weinhold, Theoretical Chemistry Institute, University of Wisconsin, Madison, (2013); b) E. D. Glendening, C. R. Landis, F. Weinhold, *J. Comp. Chem.* **2013**, *34*, 1429-1437.

[S11] K. B. Wiberg, *Tetrahedron* **1968**, *24*, 1083 - 1096.

[S12] *GaussView Version 5.0.9* R. Dennington, T. Keith, J. Millam, (2009).

***x,y,z* coordinates for K[In(NON$^{Ar}$)(NMes)] (5)**

| | | | |
|---|---|---|---|
| In | 0.02674 | 0.01891 | -0.05684 |
| K | 1.83832 | 3.43468 | 0.49211 |
| Si | 2.55028 | -2.25301 | -0.23833 |
| Si | -0.19205 | -2.78564 | -1.66087 |
| O | 1.31649 | -3.06426 | -0.99567 |
| N | 2.06960 | -0.57850 | -0.06152 |
| N | -1.03156 | -1.68416 | -0.62216 |
| N | -0.23776 | 1.97891 | 0.20759 |
| C | 3.01661 | 0.42986 | 0.15214 |
| C | 3.39885 | 0.81571 | 1.46990 |
| C | 4.34140 | 1.83559 | 1.65022 |
| H | 4.63689 | 2.11328 | 2.66597 |
| C | 4.92013 | 2.49264 | 0.56608 |
| H | 5.67222 | 3.26957 | 0.72492 |
| C | 4.54149 | 2.12782 | -0.72589 |
| H | 4.99507 | 2.63623 | -1.58083 |
| C | 3.60416 | 1.11458 | -0.95353 |
| C | 2.75641 | 0.17565 | 2.68318 |
| H | 2.20292 | -0.69720 | 2.31204 |
| C | 1.73765 | 1.12552 | 3.31686 |
| H | 2.22973 | 2.03186 | 3.71113 |
| H | 1.21843 | 0.64290 | 4.15976 |
| H | 0.96980 | 1.43012 | 2.58552 |
| C | 3.77536 | -0.31141 | 3.70894 |
| H | 4.52886 | -0.96790 | 3.24899 |
| H | 3.27314 | -0.87835 | 4.50792 |
| H | 4.30966 | 0.52438 | 4.18893 |
| C | 3.16932 | 0.79397 | -2.36821 |
| H | 2.68616 | -0.19130 | -2.32125 |
| C | 2.10686 | 1.79251 | -2.83089 |
| H | 1.25536 | 1.83074 | -2.13110 |

| | | | |
|---|---|---|---|
| H | 1.71229 | 1.52341 | -3.82341 |
| H | 2.52834 | 2.80998 | -2.90978 |
| C | 4.32409 | 0.71154 | -3.36004 |
| H | 4.79699 | 1.69222 | -3.53045 |
| H | 3.96066 | 0.35654 | -4.33671 |
| H | 5.10306 | 0.01627 | -3.01426 |
| C | 2.84028 | -3.13765 | 1.38871 |
| H | 1.95951 | -3.07344 | 2.04429 |
| H | 3.70596 | -2.72951 | 1.93218 |
| H | 3.03581 | -4.20354 | 1.19105 |
| C | 4.11129 | -2.39501 | -1.26622 |
| H | 4.49297 | -3.42701 | -1.22301 |
| H | 4.90251 | -1.72496 | -0.89381 |
| H | 3.92097 | -2.15097 | -2.32174 |
| C | 0.12376 | -2.01058 | -3.34136 |
| H | 0.62194 | -1.03361 | -3.22558 |
| H | -0.81333 | -1.84127 | -3.89285 |
| H | 0.77997 | -2.64754 | -3.95510 |
| C | -1.09820 | -4.41003 | -1.79703 |
| H | -0.55927 | -5.09668 | -2.46759 |
| H | -2.11520 | -4.26470 | -2.19215 |
| H | -1.18606 | -4.88980 | -0.81102 |
| C | -2.30426 | -1.85728 | -0.04164 |
| C | -3.43834 | -1.21516 | -0.60042 |
| C | -4.67508 | -1.34597 | 0.03518 |
| H | -5.54871 | -0.84476 | -0.38778 |
| C | -4.81381 | -2.09671 | 1.19756 |
| H | -5.78967 | -2.18736 | 1.68068 |
| C | -3.70085 | -2.72637 | 1.74415 |
| H | -3.81161 | -3.30821 | 2.66256 |
| C | -2.44209 | -2.61644 | 1.14797 |
| C | -3.31741 | -0.42196 | -1.88499 |

| | | | |
|---|---|---|---|
| H | -2.25359 | -0.15554 | -1.98878 |
| C | -3.67841 | -1.30482 | -3.08141 |
| H | -3.07693 | -2.22579 | -3.09847 |
| H | -3.51617 | -0.77118 | -4.03188 |
| H | -4.73798 | -1.60465 | -3.03693 |
| C | -4.12390 | 0.87261 | -1.88800 |
| H | -5.20915 | 0.68145 | -1.88256 |
| H | -3.90438 | 1.45361 | -2.79721 |
| H | -3.88226 | 1.50321 | -1.02046 |
| C | -1.22759 | -3.23707 | 1.81259 |
| H | -0.44690 | -3.32692 | 1.04339 |
| C | -0.68238 | -2.30667 | 2.89803 |
| H | -0.37401 | -1.33308 | 2.48367 |
| H | 0.19500 | -2.74595 | 3.39939 |
| H | -1.44691 | -2.10176 | 3.66398 |
| C | -1.48501 | -4.63303 | 2.37050 |
| H | -2.17858 | -4.61821 | 3.22623 |
| H | -0.54397 | -5.08363 | 2.72289 |
| H | -1.91488 | -5.29634 | 1.60465 |
| C | -1.48731 | 2.55268 | 0.30285 |
| C | -1.82839 | 3.63360 | -0.56620 |
| C | -3.04698 | 4.30231 | -0.43415 |
| H | -3.27171 | 5.12139 | -1.12643 |
| C | -3.99465 | 3.94981 | 0.52624 |
| C | -3.67211 | 2.87990 | 1.36752 |
| H | -4.39620 | 2.56529 | 2.12697 |
| C | -2.46702 | 2.18670 | 1.28025 |
| C | -0.88813 | 4.02399 | -1.66684 |
| H | -1.37457 | 4.70183 | -2.38308 |
| H | -0.52595 | 3.13648 | -2.20808 |
| H | 0.01420 | 4.55811 | -1.30689 |
| C | -5.30848 | 4.66683 | 0.64788 |

| | | | |
|---|---|---|---|
| H | -5.43238 | 5.12882 | 1.64166 |
| H | -6.16027 | 3.98104 | 0.50654 |
| H | -5.39923 | 5.46592 | -0.10244 |
| C | -2.21437 | 1.03537 | 2.19965 |
| H | -3.00782 | 0.94763 | 2.95482 |
| H | -1.24764 | 1.12570 | 2.71905 |
| H | -2.20814 | 0.07122 | 1.65695 |

***x,y,z* coordinates for [In(NON$^{Ar}$)(NMes)]$^{-}$ ([6]$^{-}$)**

| | | | |
|---|---|---|---|
| In | -0.10012 | 0.25503 | -0.00440 |
| Si | -2.93961 | -1.63155 | 0.23812 |
| Si | -0.36632 | -2.51031 | 1.71662 |
| O | -1.85005 | -2.66838 | 0.95651 |
| N | -2.19236 | -0.07631 | 0.02214 |
| N | 0.68497 | -1.59064 | 0.71392 |
| N | 0.61184 | 2.08057 | -0.23356 |
| C | -3.01940 | 1.03741 | -0.25467 |
| C | -3.34272 | 1.39533 | -1.58909 |
| C | -4.20626 | 2.46901 | -1.82436 |
| H | -4.45020 | 2.73988 | -2.85542 |
| C | -4.74660 | 3.20679 | -0.77822 |
| H | -5.42429 | 4.04038 | -0.98081 |
| C | -4.39217 | 2.88942 | 0.52899 |
| H | -4.78449 | 3.49089 | 1.35330 |
| C | -3.53152 | 1.82677 | 0.80978 |
| C | -2.67884 | 0.70123 | -2.76051 |
| H | -2.23278 | -0.22159 | -2.36378 |
| C | -1.54087 | 1.57226 | -3.30043 |
| H | -1.93917 | 2.50182 | -3.73995 |
| H | -0.97638 | 1.04096 | -4.08502 |
| H | -0.83854 | 1.86049 | -2.50047 |
| C | -3.64931 | 0.30994 | -3.87075 |
| H | -4.48566 | -0.29350 | -3.48625 |
| H | -3.13095 | -0.27995 | -4.64381 |
| H | -4.07789 | 1.19419 | -4.37027 |
| C | -3.06342 | 1.58229 | 2.22856 |
| H | -2.68952 | 0.54939 | 2.25299 |
| C | -1.88220 | 2.50407 | 2.54443 |
| H | -1.08946 | 2.43711 | 1.78041 |
| H | -1.44119 | 2.26073 | 3.52575 |

| | | | |
|---|---|---|---|
| H | -2.21097 | 3.55627 | 2.57215 |
| C | -4.16251 | 1.70714 | 3.27755 |
| H | -4.52936 | 2.74256 | 3.36939 |
| H | -3.77967 | 1.41290 | 4.26806 |
| H | -5.02376 | 1.06438 | 3.03933 |
| C | -3.46114 | -2.47314 | -1.35871 |
| H | -2.60946 | -2.59014 | -2.04516 |
| H | -4.24304 | -1.89750 | -1.87722 |
| H | -3.86116 | -3.47543 | -1.13630 |
| C | -4.46291 | -1.51172 | 1.33234 |
| H | -4.99986 | -2.47357 | 1.34128 |
| H | -5.15137 | -0.73247 | 0.96917 |
| H | -4.18504 | -1.26595 | 2.36824 |
| C | 0.29797 | -4.23426 | 2.02317 |
| H | -0.39546 | -4.80544 | 2.65990 |
| H | 1.27994 | -4.19815 | 2.51952 |
| H | 0.42206 | -4.77660 | 1.07406 |
| C | -0.70827 | -1.62322 | 3.33865 |
| H | -1.03876 | -0.58982 | 3.14533 |
| H | 0.19125 | -1.56917 | 3.97018 |
| H | -1.50641 | -2.12911 | 3.90512 |
| C | 1.90304 | -1.99985 | 0.15214 |
| C | 1.92695 | -2.86371 | -0.97410 |
| C | 3.15374 | -3.21380 | -1.54458 |
| H | 3.17067 | -3.87570 | -2.41477 |
| C | 4.35096 | -2.71618 | -1.04271 |
| H | 5.30103 | -2.98971 | -1.50913 |
| C | 4.32767 | -1.85062 | 0.04661 |
| H | 5.26553 | -1.43883 | 0.42562 |
| C | 3.12719 | -1.48449 | 0.65710 |
| C | 0.63288 | -3.32274 | -1.61911 |
| H | -0.16415 | -3.22068 | -0.86896 |

| | | | |
|---|---|---|---|
| C | 0.26946 | -2.39884 | -2.78337 |
| H | 0.15699 | -1.35461 | -2.45167 |
| H | -0.67915 | -2.70195 | -3.25560 |
| H | 1.05651 | -2.40733 | -3.55448 |
| C | 0.65448 | -4.78081 | -2.06632 |
| H | 1.35695 | -4.95085 | -2.89855 |
| H | -0.34426 | -5.08847 | -2.41500 |
| H | 0.94796 | -5.44816 | -1.24123 |
| C | 3.11806 | -0.56077 | 1.85717 |
| H | 2.15657 | -0.02370 | 1.82900 |
| C | 3.13877 | -1.38329 | 3.14695 |
| H | 2.32380 | -2.12089 | 3.16362 |
| H | 3.03298 | -0.73564 | 4.03282 |
| H | 4.08937 | -1.93471 | 3.23821 |
| C | 4.22770 | 0.48342 | 1.85249 |
| H | 5.22294 | 0.02865 | 1.99309 |
| H | 4.07678 | 1.19470 | 2.67914 |
| H | 4.23466 | 1.06197 | 0.91742 |
| C | 1.91864 | 2.41284 | -0.38693 |
| C | 2.82224 | 1.85866 | -1.35239 |
| C | 4.12990 | 2.33708 | -1.45604 |
| H | 4.79037 | 1.87912 | -2.20220 |
| C | 4.63059 | 3.36459 | -0.65367 |
| C | 3.74889 | 3.91647 | 0.28539 |
| H | 4.10275 | 4.72179 | 0.94126 |
| C | 2.44086 | 3.47155 | 0.43352 |
| C | 2.36933 | 0.74827 | -2.24480 |
| H | 3.12520 | 0.51948 | -3.01055 |
| H | 1.42078 | 0.99559 | -2.74904 |
| H | 2.20325 | -0.19170 | -1.68563 |
| C | 6.03856 | 3.86852 | -0.79661 |
| H | 6.56114 | 3.91219 | 0.17456 |

| | | | |
|---|---|---|---|
| H | 6.07852 | 4.88758 | -1.22309 |
| H | 6.62897 | 3.21639 | -1.45887 |
| C | 1.53627 | 4.05815 | 1.47024 |
| H | 2.02361 | 4.88173 | 2.01527 |
| H | 1.22358 | 3.28706 | 2.19600 |
| H | 0.60032 | 4.42173 | 1.01602 |
